# Cryptophycin unit B analogues

**DOI:** 10.3762/bjoc.21.40

**Published:** 2025-03-07

**Authors:** Thomas Schachtsiek, Jona Voss, Maren Hamsen, Beate Neumann, Hans-Georg Stammler, Norbert Sewald

**Affiliations:** 1 Department of Chemistry, Organic and Bioorganic Chemistry, Bielefeld University, Universitätsstraße 25, 33615 Bielefeld, Germany; 2 Department of Chemistry, Inorganic and Structural Chemistry, Bielefeld University, Universitätsstraße 25, 33615 Bielefeld, Germany

**Keywords:** cancer treatment, cryptophycins, drug conjugates, payload, targeted delivery

## Abstract

Drug conjugates using toxic payloads are a promising approach for selectively combating cancer while sparing healthy tissue. The lack of highly cytotoxic and at the same time selective therapeutics against cancer is an ongoing challenge. Cryptophycins are a class of cyclic depsipeptides renowned for their high cytotoxicity in the picomolar range often combined with efficacy against multidrug-resistant tumour cell lines. However, cryptophycins failed as stand-alone drugs in cancer treatment, and their naturally occurring derivatives lack a covalent attachment handle. By making use of drug conjugates, toxic payloads such as cryptophycins can be selectively delivered to the target site. We present the synthesis of two conjugable cryptophycins with amino groups in unit B, representing potential payloads for drug conjugates particularly effective against multidrug-resistant cancers.

## Introduction

**Figure 1:**
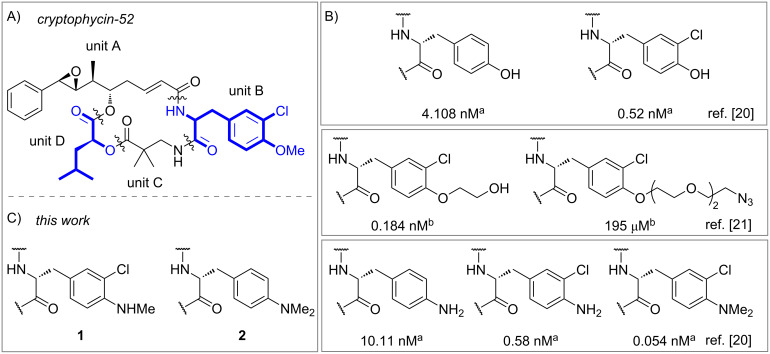
A: Structure of cryptophycin-52. B: Cryptophycin-52 derivatives modified with conjugation handles in unit B. IC_50_ values given against (a) CCRF-CEM [20] and (b) KB-3-1 [21] cell lines. C: Cryptophycin-52 derivatives synthesised in this work.

Cryptophycins emerged as highly potent cytotoxins for the use in targeted cancer therapy [1]. Originally discovered over three decades ago by isolation from cyanobacteria [2], their extraordinarily high cytotoxicity was apparent and attracted attention, not least because of their still high efficacy against multidrug-resistant (MDR) cells [3]. Cryptophycin-52, a synthetic development candidate by Eli Lilly based on the initially discovered cryptophycin-1, failed in clinical studies as a cytotoxic drug on its own due to severe side effects [4]. However, the embedment of cryptophycins as payloads in drug conjugates, increases specificity and minimises adverse effects [5–6]. Drug conjugates usually consist of three units, where the payload is connected to the homing device by a linker. For covalent attachment of the drug to the linker a suitable functional group is needed such as an amino, hydroxy, carboxy or sulfhydryl group [7]. Since the cryptophycins’ discovery, considerable efforts were made for the establishment of structure–activity relationship (SAR) studies between derivatisations of all four units (A to D) (Figure 1A) of cryptophycin-52 analogues with functional groups [8]. While many modifications of cryptophycin decrease the cytotoxicity drastically, some viable attachment points for conjugation were found, mainly including the *para*-phenyl position of unit A [9–11] or derivatisation of unit A’s epoxide moiety into a halohydrin (-glycinate) [5,12–17]. Cryptophycins modified at the α-position of unit C [18] and, most recently, various derivatives with modifications in unit D [19] were established as potent and conjugable payloads by our group. Modifications of unit B with conjugation handles are scarcely explored and mainly include the exchange of the *para*-methoxy group (Figure 1B). The sole exchange of the *para*-methoxy group of cryptophycin-52 (IC_50_ = 22 pM) by a hydroxy group reduces the cytotoxicity by approximately only one order of magnitude (IC_50_ = 0.52 nM) when tested on CCRF-CEM T lymphoblasts [20]. Loss of the *meta*-chloro substituent shows a similar trend. Functionalisation of the hydroxy group with ethylene glycol residues further decreases cytotoxicity, whereby this effect increases with increasing chain length [21]. The exchange by an amino group showed a similar trend, however, *N*,*N*-dimethylation of the amino group again increased cytotoxicity significantly [20]. Nevertheless, this cryptophycin might only be conjugated via an ammonium-based self-immolative linker [22]. We envisioned the synthesis of, firstly, *m*-chloro-*p*-(methylamino) derivative **1** to dissect the structure–activity relationship between the primary (IC_50_(CCRF-CEM) = 0.58 nM), secondary, and tertiary amine (IC_50_(CCRF-CEM) = 54 pM) derivatives [20]. Secondly, *p*-(dimethylamino) derivative **2** was synthesised to investigate the effect of *N*-alkylation on the non-chlorinated unit B derivatives.

## Results and Discussion

**Scheme 1:**
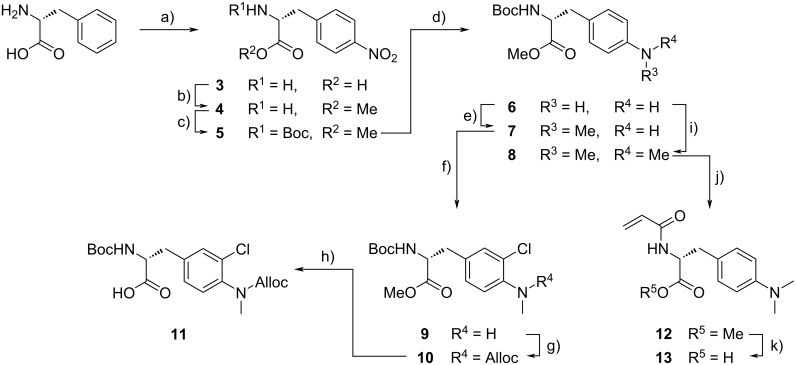
Synthesis of modified unit B derivatives. a) HNO_3_, H_2_SO_4_, 0 °C, 5 h, 48% (isolated as monohydrate); b) SOCl_2_, MeOH, 0 °C, 90 min, then reflux, 17 h, 95%; c) Boc_2_O, NEt_3_, MeCN, H_2_O, rt, 22 h, 88%; d) H_2_, Pd/C, MeOH, rt, 25 h, 98%; e) either: formalin, NaBH_3_CN, HOAc, MeOH, rt, 20 min, 37%, or: formalin, (NH_4_)HCO_2_, Pd/C, MeOH, rt, 20 min, 35%; f) *N*-chlorosuccinimide, MeCN, reflux, 16 h, 77%; g) AllocCl, NaHCO_3_, CHCl_3_, H_2_O, rt, 5 h, 89%; h) LiOH·H_2_O, MeOH, tetrahydrofuran, H_2_O, 0 °C to rt, 3 h, 99%; i) formalin, NaBH_3_CN, MeCN, rt, 55 min, 61%; j) 1. TFA, CH_2_Cl_2_, 0 °C, 1.5 h; 2. acryloyl chloride, NEt_3_, CH_2_Cl_2_, 0 °C to rt, 19 h, 63% over two steps; k) LiOH·H_2_O, MeOH, tetrahydrofuran, H_2_O, 0 °C, 3.5 h, 82% with unconverted **12** (18 mol %).

**Figure 2:**
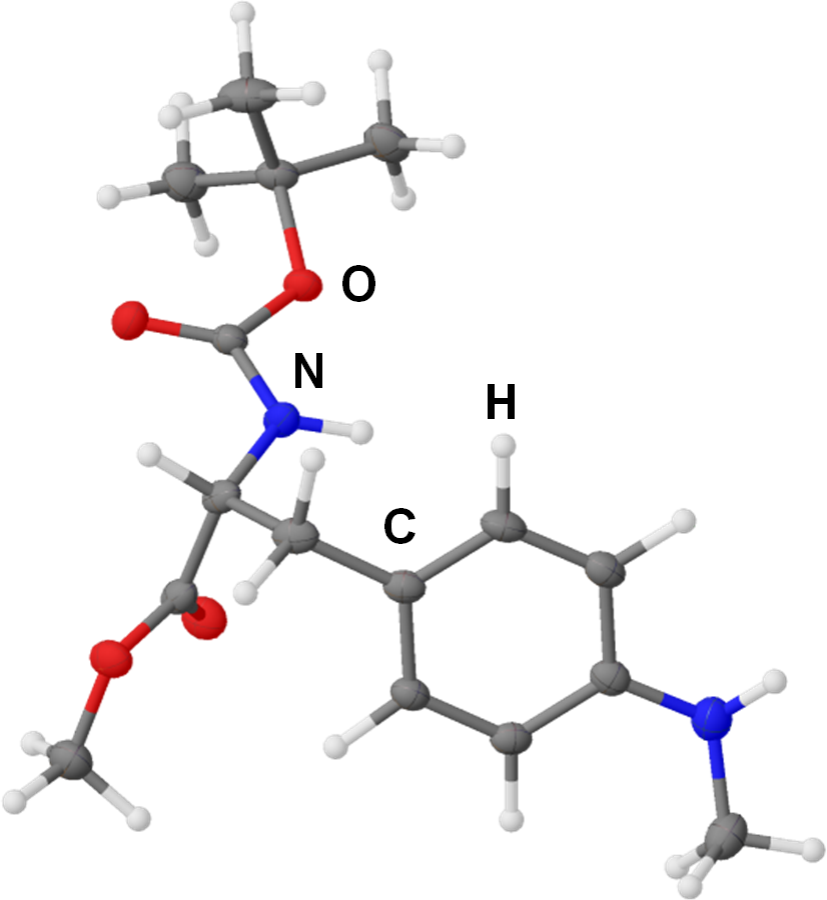
Molecular structure of Boc-ᴅ-Phe(4-NHMe)-OMe **7** as determined by single-crystal X-ray diffraction measurements. Thermal ellipsoids depicted at 50% probability.

For the synthesis of unit B derivatives with amino groups instead of the naturally occurring methoxy group ᴅ-phenylalanine served as the fundamental substrate (Scheme 1). Nitration [23] followed by methyl ester formation and *N-*Boc-protection [24] provided nitroarene **5** in 40% yield over three steps. Reduction of the nitro group was performed with Pd/C and hydrogen to obtain aniline **6** in 98% yield, which served as a precursor for mono- and dimethylated unit B derivatives **7** and **8**, respectively. While dimethylaniline **8** was obtained in good yield of 61% through reductive amination with excess formaldehyde and NaBH_3_CN as reductant, the selective installation of only one methyl group, providing monomethyl aniline **7**, proved to be more troublesome. Either reductive amination using the same protocol, but under strict control of equivalents and pH, or Leuckart–Wallach-like reaction with ammonium formate and Pd/C [25] provided monomethylaniline **7** in 37% and 35% yield, respectively. The absolute structure of monomethylaniline **7** was confirmed by single-crystal X-ray diffraction measurements (Figure 2). Compound **7** crystallised in the monoclinic space group *P*2_1_ with *R*_1_ = 0.0285 clearly showing the expected (*R*)-configuration with a Flack parameter of −0.07(6).

Starting from monomethylaniline derivative **7**, the synthesis of the final building block was finalised by chlorination [20], Alloc protection and saponification [21] to obtain free acid **11**. Exchange of the Boc protecting group of dimethylaniline **8** to an acryloyl substituent and subsequent saponification furnished dimethylamine building block **13**.

For the macrocycle assembly, especially the ring closure, we decided on two different routes. While the cryptophycin containing a dimethylamino motif did not require an additional protecting group, ring closure was performed through alkene cross metathesis, which has been accomplished reliably and with good yields for other cryptophycins [11,26–27]. However, for the synthesis of a cryptophycin with a monomethylated amino group in unit B a suitable protecting group, i.e., allyloxycarbonyl (Alloc), must be used. Since the presence of this allylic double bond would most likely interfere with a clean reaction outcome after alkene cross metathesis, we decided for a more classical ring-closure strategy through macrolactamisation [21].

**Scheme 2:**
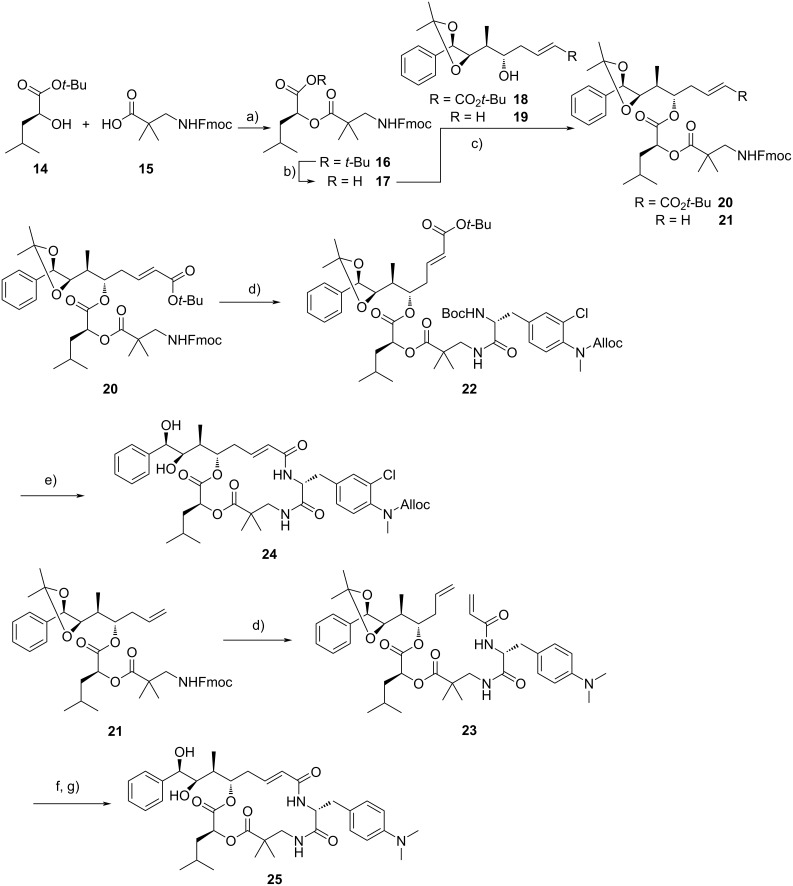
Synthesis of cryptophycin diols **24** and **25**. a) EDC·HCl, DMAP, NEt_3_, CH_2_Cl_2_, 0 °C to rt, 22 h, 60%; b) TFA, CH_2_Cl_2_, 0 °C, 4 h, 100%; c) 2,4,6-trichlorobenzoyl chloride, DMAP, NEt_3_, THF, 0 °C or 0 °C to rt, 3h, tetrahydrofuran, 77% (for **20**)/88% (for **21**); d) 1. piperidine, *N*,*N*-dimethylformamide, rt, 1.5 h; 2. unit B (**11** for **20** and **13** for **21**), HOAt, NEt_3_, EDC·HCl, CH_2_Cl_2_, 0 °C to rt, 18 h, 49% (for **22**)/38% (for **23**); e) 1. TFA, CH_2_Cl_2_, 0 °C to rt, 2.5 h; 2. HATU, HOAt, *N*,*N*-dimethylformamide 0 °C for 2 h and at rt for 20 min, 11%; f) Grubbs II catalyst, CH_2_Cl_2_, reflux, 3 h, 84%; g) TFA, H_2_O, CH_2_Cl_2_, 0 °C to rt, 5 h, 91%. EDC·HCl = 1-ethyl-3-(3-dimethylaminopropyl)carbodiimide hydrochloride; DMAP = 4-(dimethylamino)pyridine; TFA = trifluoroacetic acid; HOAt = 1-hydroxy-7-azabenzotriazole; HATU = 1-[bis(dimethylamino)methyliumyl]-1*H*-1,2,3-triazolo[4,5-*b*]pyridinium 3-oxide hexafluorophosphate.

The syntheses of required unit A [28], C [29–30], and D [31] building blocks were accomplished as described previously. *tert*-Butyl-protected leucic acid **14** and Fmoc-β-aminopivalic acid (**15**) were connected by Steglich esterification (Scheme 2) and after cleavage of the *tert*-butyl ester group coupled to either one of the two unit A precursors **18** and **19** by Yamaguchi esterification. CDA fragments **20** and **21** were connected to unit B derivatives **11** and **13** through amidation which provided *seco*-cryptophycins **22** and **23** in yields of 49% and 38%, respectively. Starting from *seco*-cryptophycin **22** acidolysis was followed by macrolactamisation to obtain diol **24**, albeit in poor yield of 11%. LC–MS analyses revealed that the low yield cannot be attributed to either incomplete acidolysis or incomplete conversion of the deprotected *seco*-cryptophycin during macrolactamisation. Rather, LC–MS analyses indicate the major presence of a fully deprotected and trifluoroacetylated *seco*-cryptophycin, most likely a TFA ester of one of the free hydroxy groups, a finding which might reason the comparably low yields (21–25%) of structurally similar unit B analogues reported earlier [21]. Contrary, diol **25** was obtained through Grubbs metathesis and subsequent acetonide cleavage in a superior yield of 76%.

**Scheme 3:**
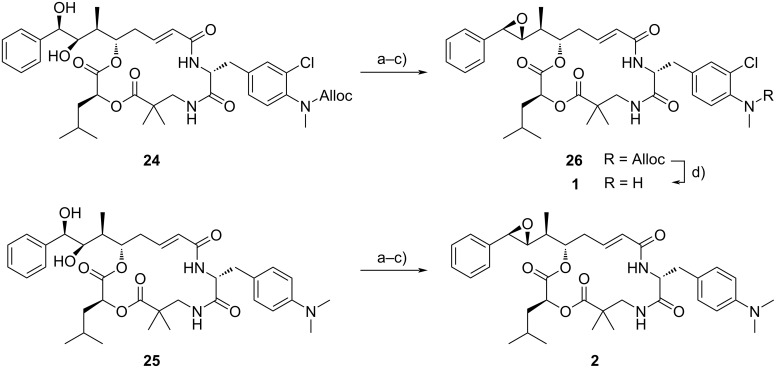
Three-step diol–epoxide transformation starting from diols **24** and **25**. a) (MeO)_3_CH, pyridinium *p*-toluenesulfonate, CH_2_Cl_2_, rt, 3 h; b) AcBr, CH_2_Cl_2_, rt, 3–4 h; c) K_2_CO_3_, MeOCH_2_CH_2_OMe, HOCH_2_CH_2_OH, rt, 5 min, 89% (for **26**)/14% (for **2**), over three steps each; d) Pd(PPh_3_)_4_, morpholine, CH_2_Cl_2_, rt, 90 min, 74%.

The finalising steps to obtain epoxides **26** and **2** (Scheme 3) were a diol–epoxide transformation [11,19,32], including firstly the formation of a cyclic orthoester, secondly the formation of a bromohydrin formate, and thirdly ring closure to obtain the epoxides **26** and **2** in 89% and 14% yield over three steps each, respectively.

While the synthesis of tertiary amine **2** was finished, final Alloc cleavage from **26** under Tsuji–Trost-like conditions [19] provided secondary amine **1** in 74% yield.

The cytotoxicity of Alloc-protected cryptophycin **26** and both free amines **1** and **2** were tested against the human cervix carcinoma cell line KB-3-1 and its MDR subclone KB-V1 (Table 1) [33–34].

**Table 1:** IC_50_ values of synthesised cryptophycins against KB-3-1 and KB-V1 cell lines.

	IC_50_ [nM]	

	KB-3-1	KB-V1

**26**	374	>1000
**1**	0.313	7.76
**2**	6.36	218

The *m*-chloro-*p*-(methylamino) derivative **1** showed high cytotoxicity with IC_50_ (KB-3-1) = 313 pM, which is perfectly between the values of the primary (IC_50_ (CCRF-CEM) = 580 pM) and tertiary (IC_50_ (CCRF-CEM) = 54 pM) amine derivatives (Figure 1B) [20,35], showing a strict increase in cytotoxicity with increasing degree of aniline methylation. The same trend was also observed for unit D derivatives modified with amino groups [19] and likely hints to favoured hydrophobic interactions around the binding pocket at unit B and D. In comparison, the cytotoxicity of Alloc-protected amine **26** decreased significantly (IC_50_ (KB-3-1) = 374 nM), which could be due to higher steric bulk as this is known to be less well tolerated; cf. unit B *p*-alkyloxy derivatives (Figure 1B) [21]. Tertiary amine **2** showed IC_50_ (KB-3-1) = 6.36 nM which is similar to its non-alkylated congener (IC_50_ (CCRF-CEM) = 10.11 nM) but about two orders of magnitude higher than its chlorinated derivative (IC_50_ (CCRF-CEM) = 54 pM) (Figure 1B) [20]. Notably, both free amines still show nanomolar activity against the MDR cell line KB-V1 (Table 1) with resistance factors *F*_R_ (being the ratio between IC_50_ (KB-V1) and IC_50_ (KB-3-1)) of 25 (**1**) and 34 (**2**), thus being comparably much more potent against KB-V1 cells than for example the unit D analogues (*F*_R_ ≈ 10^3^) [19].

## Conclusion

In summary, we synthesised two new conjugable cryptophycin analogues **1** and **2** with amino groups in unit B, by utilising either macrolactamisation or Grubbs metathesis for ring closure. These synthetic routes should generally allow access to diverse other unit B cryptophycin analogues. Both cryptophycins **1** and **2** showed high cytotoxicity with 313 pM (**1**) and 6.36 nM (**2**) and outstandingly low resistance factors. Furthermore, the new cryptophycin **1** confirms the correlation between degree of alkylation and cytotoxicity of *m*-chloro-*p*-amino unit B derivatives. Since MDR is responsible for over 90% of deaths in cancer patients and MDR-selective therapeutics are lacking [36], we expect that cryptophycins **1** and **2** will be considered as viable cytotoxins for the use in targeted tumour therapy, especially against MDR cancers.

## Supporting Information

The supplementary crystallographic data for this article can be obtained free of charge from the Cambridge Crystallographic Data Centre (CCDC) via https://www.ccdc.cam.ac.uk/structures under reference number CCDC 2400694 for Boc-ᴅ-Phe(4-NHMe)-OMe (**7**).

File 1 Experimental section, NMR spectra and cellular proliferation assays.

## Data Availability

All data that supports the findings of this study is available in the published article and/or the supporting information of this article.
