# Supplementary material for: Cryptophycin unit B analogues
Source: Beilstein J Org Chem. 2025 Mar 7;21:526–32. doi: 10.3762/bjoc.21.40 (PMC11897651; doi:10.3762/bjoc.21.40)
Supplement: File 1 — Experimental section, NMR spectra and cellular proliferation assays. [file Beilstein_J_Org_Chem-21-526-s001.pdf]

BEILSTEIN JOURNAL OF ORGANIC CHEMISTRY

Supporting Information

for

Cryptophycin unit B analogues

Thomas Schachtsiek, Jona Voss, Maren Hamsen, Beate Neumann, Hans-Georg Stammler and Norbert Sewald

Beilstein J. Org. Chem. **2025,** *21,* 526–532. doi:10.3762/bjoc.21.40

Experimental section, NMR spectra and cellular proliferation assays

License and Terms: This is a supporting information file under the terms of the Creative Commons Attribution License (https://creativecommons.org/licenses/by/4.0). Please note that the reuse, redistribution and reproduction in particular requires that the author(s) and source are credited and that individual graphics may be subject to special legal provisions.
The license is subject to the *Beilstein Journal of Organic Chemistry* terms and conditions: (https://www.beilstein-journals.org/bjoc/terms)

Table of contents

1 General methods and materials S2

2 General procedures S9

3 X-ray diffraction data S10

4 Experimental protocols S11

4.1 Unit B S11

4.1.1 H-D-Phe(4-NO_2)-OH **3**·H_2O S11
4.1.2 H-D-Phe(4-NO_2)-OMe (**4**) S11
4.1.3 Boc-D-Phe(4-NO_2)-OMe (**5**) S12
4.1.4 Boc-D-Phe(4-NH_2)-OMe (**6**) S12
4.1.5 Boc-D-Phe(4-NHMe)-OMe (**7**) S13
4.1.6 Boc-D-Phe(4-NMe_2)-OMe (**8**) S14
4.1.7 Boc-D-Phe(3-Cl)(4-NHMe)-OMe (**9**) S15
4.1.8 Boc-D-Phe(3-Cl)(4-NMeAlloc)-OMe (**10**) S15
4.1.9 Boc-D-Phe(3-Cl)(4-NMeAlloc)-OH (**11**) S16
4.1.10 Acryloyl-D-Phe(4-NMe_2)-OMe (**12**) S17
4.1.11 Acryloyl-D-Phe(4-NMe_2)-OH (**13**) S18

4.2 Fragments CDA S19

4.2.1 Fmoc-uC-uD-O*t*-Bu **16** S19
4.2.2 Fmoc-uC-uD-OH **17** S19
4.2.3 Fmoc-uC-uD-uA-CO_2*t*-Bu **20** S20
4.2.4 Fmoc-uC-uD-uA-H **21** S21

4.3 Synthesis of cryptophycin-uB[D-Phe(3-Cl)(4-NHMe)] **1** S22

4.3.1 uB[D-Phe(3-Cl)(4-NMeAlloc)]-modified *seco*-cryptophycin **22** S22
4.3.2 Modified cryptophycin-uA[diol]-uB[D-Phe(3-Cl)(4-NMeAlloc)] **24** S23
4.3.3 Cryptophycin-uB[D-Phe(3-Cl)(4-NMeAlloc)] **26** S24
4.3.4 Cryptophycin-uB[D-Phe(3-Cl)(4-NHMe)] **1** S25

4.4 Synthesis of cryptophycin-uB[D-Phe(4-NMe_2)] **2** S27

4.4.1 uB[D-Phe(4-NMe_2)]-modified *seco*-cryptophycin **23** S27
4.4.2 Modified cryptophycin-uA[diol]-uB[D-Phe(4-NMe_2)] **25** S28
4.4.3 Cryptophycin-uB[D-Phe(4-NMe_2)] **2** S30

5 References S32

6 Appendix S33

6.1 NMR spectra S33

6.2 Cellular proliferation assays S49

1 General methods and materials

Synthesis of units A, C and D

The required building blocks **14**, **15**, **18** and **19** (Figure S1) were synthesised according to the literature.

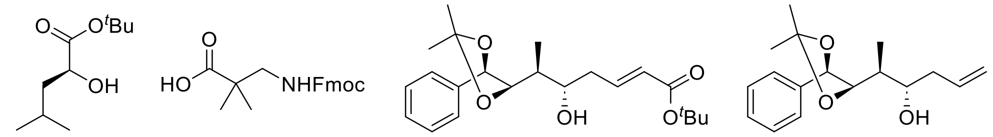

14 **15** **18** **19**

Figure S1: Molecular structures of building blocks **14**, **15**, **18** and **19**.

Unit A derivatives **18** and **19** were synthesised following the procedures described by Sewald and co-workers starting from phenylacetaldehyde.[1] Unit C derivative **15** was synthesised from β-chloropivalic acid as described previously by our group.[2,3] *tert*-Butyl-protected leucic acid **14** was synthesised after instructions from Kessler and co-workers starting from leucine.[4]

Reagents and solvents

Unless otherwise noted, all reagents and solvents were obtained from commercial sources and used without further purification. Dichloromethane (DCM), ethyl acetate (EtOAc), petroleum ether (PE), cyclohexane (CyH) and diethyl ether (Et_2O) for column chromatography or aqueous workups were purchased as technical grade quality and purified by distillation prior use. Dichloromethane was purified by distillation over K_2CO_3.

For inert reactions dry solvents were used. Therefore tetrahydrofuran (THF) was purchased in p.A. quality. THF was kept over KOH before dried with sodium/benzophenone under reflux and distilled freshly before use. Dichloromethane (DCM) was purchased in p.A. quality, pre-dried over $CaCl_2$ and dried over CaH_2 under reflux and distilled freshly before use. DMF was purchased in p.A. quality and dried over activated molecular sieves (4 Å). Methanol (MeOH) and acetonitrile (ACN) was purchased in p.A. quality and dried over activated molecular sieves (3 Å).

Inert reactions

Schlenk conditions: Reaction under inert conditions were carried out under exclusion of moisture and oxygen under an argon atmosphere in dried glassware. The argon gas was supplied from Linde (quality 4.0) and dried additionally through a column filled with phosphorus pentoxide (Sicapent®, Merck). When opening an inert reaction, a constant argon flow was used to prevent the reaction conditions from moisture and oxygen and the glassware was

closed again as fast as possible. If possible, a pierceable septum was used to add liquids via a needle equipped syringe to the reactions.

Solvents were removed on a rotary evaporator at 40 °C and appropriately reduced pressure. Solvent residues were removed at rt and 0.001–0.1 mbar.

Column chromatography

Unless otherwise noted, column chromatography means normal phase column chromatography, which was performed using silica gel 60 (40–63 µm, Macherey-Nagel). The eluents mixture is noted in the experimental procedure. The silica gel was slurried in the eluent solvent and poured into a suitable glassware equipped with a plug glass wool and a layer of sea sand to hold the silica gel in place. The slurry was left to sit for 5 to 60 min. For each column chromatography a freshly prepared column was used. Fractions were analysed using TLC and combined when they were identical. After purification by column chromatography solvents of product fraction was removed using rotary evaporator and high vacuum.

Automated column chromatography

A Büchi Reveleris X2 was used for automated column chromatography. The columns were selected in the quality and size recommended by the manufacturer. For loading, the crude products were adsorbed on silica gel and injected with a solid loader or as solutions dissolved in the starting eluent mixture. Detection was performed using a UV detector at the wavelengths (254 nm, 265 nm, 280 nm) and an ELSD light scattering sensor.

Preparative reversed-phase HPLC

Preparative RP-HPLC was performed on a Merck-Hitachi system (controller: D-7000, pump: L-7150, detector: L-7420). Fractions were collected based on UV absorption at 220 nm and analysed by HPLC or HPLC–MS. Identical fractions were combined and freeze-dried.

Eluent A: $H_2O/CH_3CN/TFA$ = 94.9/5/0.1

Eluent B: $H_2O/CH_3CN/TFA$ = 5/94.9/0.1

Thin-layer chromatography (TLC)

Thin layer chromatography was performed using silica gel 60 coated aluminium sheets with fluorescence indicator F254 (Merck). Samples are absorbed onto the TLC plate which is developed in a chamber equipped with suitable premixed eluents. The eluent mixture is given in the corresponding experimental procedure. Spots were identified with a wavelength of 254 nm or 366 nm.

Thin-layer chromatography–MS (TLC–MS)

TLC is developed according to the TLC method section above. The TLC spots are introduced using an in-house built TLC interface operated with a Hitachi L-6000 LC pump (isopropanol, flow rate 400 µL/min). The isopropanol flow is introduced into a ZQ2000 single quadrupole mass spectrometer (Waters, Manchester, UK) equipped with an ESI source, operating with a spray voltage of 3.5 kV. Nitrogen served both as the nebuliser gas and the dry gas and is generated by a nitrogen generator NGM 11. The mass axis was externally calibrated with ESI-L Tuning Mix (Agilent Technologies, Santa Clara, CA, USA) as calibration standard. Scan accumulation and data processing was performed with MassLynx 4.1 (Waters, Manchester, UK).

NMR spectroscopy

NMR spectra were recorded on a Bruker Avance III 500, Bruker Avance III HD 500 (500 MHz for ^{1}H and 126 MHz for ^{13}C each) or Bruker Avance III 600 (600 MHz for ^{1}H and 151 MHz for ^{13}C) spectrometer, if not otherwise stated at 25 °C, using residual protonated solvent signals as internal standards.[5]

$CHCl_3$	δ 7.26 ppm (^{1}H NMR) and δ 77.16 ppm (^{13}C NMR).
CHD_2OD	δ 3.31 ppm (^{1}H NMR) and δ 49.0 ppm (^{13}C NMR).
$D_2HCS(=O)CD_3$	δ 2.50 ppm (^{1}H NMR) and δ 39.5 ppm (^{13}C NMR).
DHO	δ 4.79 ppm (^{1}H NMR).

The yields of products containing solvent residues, by-products or impurities that could be structurally identified by ^{1}H NMR spectroscopy were corrected through integration of the ^{1}H NMR spectra. Signal assignment of NMR spectra (^{1}H, ^{13}C{^{1}H}) was supported by 1D- and 2D-NMR spectra (^{1}H,^{1}H-COSY, ^{1}H,^{1}H-TOCSY, ^{13}C-DEPT-135, ^{1}H,^{13}C-HMQC, ^{1}H,^{13}C-HMBC) and follows the nomenclature depicted below. Splitting patterns are abbreviated as follows: singlet (s), doublet (d), triplet (t), quartet (q), multiplet (m), broad (br). Superscript capital letters (“A”, “B”, …) denote chemically equivalent but magnetically inequivalent nuclei. NMR signal assignment as reported previously (see below).[6]

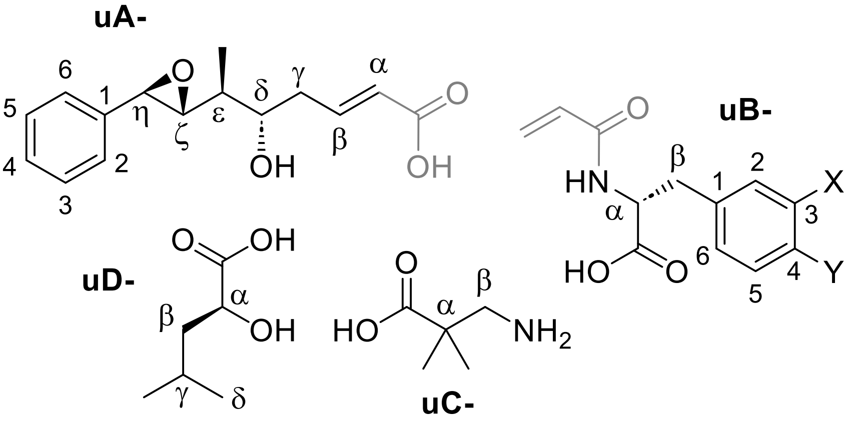

Redrawn from Sewald *et al.*[6] X, Y = functional groups.

Analytical reversed-phase HPLC (HPLC)

Analytical HPLC samples were measured at a CBM-20A chromatography system (Shimadzu) consisting of autosampler: Nexera XR SIL-20Axr, pump: Nexera XR LC-20ADXR, column oven: CTO-20A, and diode array detector SPD-M20A IVDD. As column a Phenomenex Luna C18 column (part number: 00D-4251-B0, pore size: 100 Å, particle size: 3 µm, length: 100 mm, internal diameter: 2.0 mm) at 40 °C column oven temperature. If possible, samples were injected with concentration of 0.5 mM and 3 µL injection volume. Unless otherwise noted the standard method was used:

Eluent A: H_2O/TFA = 99.9/0.1

Eluent B: CH_3CN/TFA = 99.9/0.1

Flowrate: 650 µl/min

Standard method:

0	min	95%	A	5%	B
5.5	min	5%	A	95%	B
6.0	min	5%	A	95%	B
6.1	min	95%	A	5%	B
9.0	min	95%	A	5%	B

Reversed-phase HPLC–MS (LC–MS)

HPLC–MS samples were recorded on an Agilent 1200 HPLC System (Agilent Technologies, Santa Clara, CA, USA) consisting of an autosampler, degasser, binary pump, column oven and diode array detector. For separation a Phenomenex Luna C18 column (part number: 00D-4251-B0, pore size: 100 Å, particle size: 3 µm, length: 100 mm, internal diameter: 2.0 mm) was

used at 40 °C column oven temperature. The HPLC was coupled directly to an Agilent 6220 ESI time-of-flight mass spectrometer (Agilent Technologies, Santa Clara, CA, USA) using a Dual-ESI source at 2.5 kV spray voltage. Nitrogen generated by a nitrogen generator NGM 11 served both as the nebuliser gas and the dry gas. Spectra were recorded in a range of 150 to 2500 *m*/*z* with a rate of 2 spectra per second and analysed with MassHunter Workstation Acquisition B.07.00 software.

The mass spectrometer was calibrated with external standard (ESI-L Tuning Mix, Agilent Technologies, Santa Clara, CA, USA) as calibration standard. If possible, samples were injected with concentration of 0.5 mM and 5 µL injection volume. Unless otherwise noted the standard-method was used:

Eluent A: $H_2O/CH_3CN/HCOOH$ = 94.9/5/0.1

Eluent B: $H_2O/CH_3CN/HCOOH$ = 5/94.9/0.1

Flowrate: 300 µl/min

Standard method:

0	min	100%	A	0%	B
10.0	min	2%	A	98%	B
11.0	min	2%	A	98%	B
11.5	min	100%	A	0%	B
15.0	min	100%	A	0%	B

Isocratic method:

0	min	100%	A	0%	B
3.0	min	2%	A	98%	B
11.0	min	2%	A	98%	B
11.5	min	100%	A	0%	B
15.0	min	100%	A	0%	B

HRMS

High resolution mass spectra were recorded on an Agilent 6220 accurate-mass TOF LC-MS or Synapt G2Si.

This protocol was adopted from and with permission of the mass department University Bielefeld:
ESI accurate mass measurements are acquired using an Agilent 6220 time-of-flight mass spectrometer (Agilent Technologies, Santa Clara, CA, USA) in extended dynamic range mode equipped with a dual-ESI source, operating with a spray voltage of 2.5 kV. Nitrogen served both as nebuliser gas and dry gas. Nitrogen was generated by a nitrogen generator NGM 11. Samples were dissolved in acetonitrile and introduced with a 1200 HPLC system consisting of an autosampler, degasser, binary pump, column oven and diode array detector (Agilent Technologies, Santa Clara, CA, USA) using a C18 Hypersil Gold column (length: 50 mm, diameter: 2.1 mm, particle size: 1.9 µm) with a short gradient (in 4 min from 0% B to 98% B, back to 0% B in 0.2 min, total run time 7.5 min) at a flow rate of 250 µL/min and column oven temperature of 40 °C. HPLC solvent A consists of 94.9% water, 5% acetonitrile and 0.1% formic acid, solvent B of 5% water, 94.9% acetonitrile and 0.1% formic acid. The mass axis was externally calibrated with ESI-L Low Concentration Tuning Mix (Agilent Technologies, Santa Clara, CA, USA) as calibration standard. The mass spectra are recorded in both profile and centroid mode with the MassHunter Workstation Acquisition B.04.00 software (Agilent Technologies, Santa Clara, CA, USA). MassHunter Qualitative Analysis B.07.00 software (Agilent Technologies, Santa Clara, CA, USA) was used for processing and averaging of several single spectra.

Synapt G2Si nano-ESI measurements are performed using a Q-IMS-TOF mass spectrometer Synapt G2Si (Waters GmbH, Manchester, UK) in resolution mode, interfaced to a nano-ESI. Nitrogen serves both as the nebuliser gas and the dry gas for nano-ESI. Samples were dissolved in solvent (e.g., H_2O, acetonitrile, methanol) and introduced by static nano-ESI using in-house pulled glass emitters. The mass axis was internally calibrated with the doubly protonated GluFib ion or the protonated LeuEnk ion as internal calibration standard. Scan accumulation and data processing was performed with MassLynx 4.1 (Waters GmbH, Manchester, UK) on a PC Workstation. The spectra shown here were generated by the accumulation and averaging of several single spectra. Determination of exact masses were performed using centroided data.

Cell assays

Cell assays were performed on a modified protocol published by Sewald et al.[6]

To ensure that the cytotoxicity results were not influenced by traces of impurities, the cryptophycin samples were, if necessary, purified again by RP-HPLC.

The KB-3-1 and KB-V1 cells were cultivated as a monolayer in Dulbecco's modified Eagle medium (DMEM, PAN Biotech, Cat. No. P04-03590) with glucose ($4.5\ g \cdot L^{-1}$), ʟ-glutamine, sodium bicarbonate ($3.7\ g \cdot L^{-1}$) sodium pyruvate and phenol red, supplemented with 7.5% (KB-3-1) and 12.5% (KB-V1) fetal bovine serum (FBS Supreme, South America origin, 0.2 µM sterile filtered, PAN Biotech, Cat. No. P30-3031). $50\ \mu g \cdot mL^{-1}$ penicillin-streptomycin is added for the KB-V1 cells. The cells were maintained at 37 °C and 5.3% CO_2/humidified air. KB-V1 cells were continuously selected during cultivation with vinblastine sulfate (150 nm). On the day before the test, the cells (70% confluence) were detached with trypsin/ethylenediaminetetraacetic acid (EDTA) solution (0.05%/0.02% in Dulbecco's phosphate buffered saline [DPBS]) and plated in sterile 96-well plates in a density of 4000 cells in 100 µL medium per well. The dilution series of the compounds were prepared from stock solutions in DMSO of concentrations of 1 mm or 10 mm. The stock solutions were diluted with culture medium (12.5% FBS [KB-V1]; 7.5% FBS [KB-3-1]) at least 50 times. Some culture medium was added to the wells to adjust the volume of the wells to the wanted dilution factor. The dilution prepared from stock solution was added to the wells. Each concentration was tested in typically at least twelve replicates. Dilution series were prepared by pipetting liquid from well to well. The control contained the same concentration of DMSO as the first dilution. After incubation for 72 h at 37 °C and 5.3% CO_2/humidified air, 30 µL of a 175 µM aqueous resazurin solution (116 mM NaCl, 26 mM $NaHCO_3$, pH 7.3) was added to each well. The cells were incubated at the same conditions for 6 h. Subsequently, the fluorescence was measured. The excitation was effected at a wavelength of 530 nm, whereas the emission was recorded at a wavelength of 588 nm. The IC_{50} values were calculated as a sigmoidal dose–response curve using GraphPad Prism 4.03. The IC_{50} values equal the drug concentrations, at which vitality is 50% and were calculated following formula 1:

$$y = y_{\mathrm{min}} + \frac{(y_{\mathrm{max}} - y_{\mathrm{min}})}{1 + 10^{\mathrm{lg}(IC_{50} - x)}} \tag{1}$$

$x = \mathrm{lg}(c[\text{cryptophycin}])$; y = vitality with boundaries y_{max} and y_{min}

2 General procedures

General procedure GP I – ortho ester formation[6]

Cryptophycin diol (1 equiv) was dissolved in dichloromethane (dry, 30–40 mL/mmol) and under argon protection atmosphere and ice bath cooling trimethyl orthoformate (10 mL/mmol) and pyridinium *p*-toluene sulfonate (2.5 equiv) were added and the reaction solution was stirred for 2 to 3 h. Filtration over silica (dichloromethane:ethyl acetate: 1:1), and subsequent drying in high vacuum yielded the product.

General procedure GP II – bromohydrin formation[6]

Cryptophycin ortho ester (1 equiv) was dissolved in dichloromethane (15 mL/mmol) and acetyl bromide solution (0.5 M in DCM, 2.5 equiv) was added and stirred for 3 to 6 hours at room temperature. The reaction solution was added to sodium hydrogen carbonate solution (half sat., 250 mL). The aqueous phase was extracted with dichloromethane (3 × 20 mL), the combined organic phases were dried over magnesium sulfate, and the solvent was removed under reduced pressure. The crude product was dried overnight under high vacuum.

General procedure GP III – preparation of a reaction solution for epoxide formation[6]

An emulsion of abs. ethylene glycol (2.5 mL), abs. DME (5.0 mL) and potassium carbonate (210 mg, 1.51 mmol) was freshly prepared over 3 Å molecular sieves (320 mg) and homogenised by vortex and ultrasound.

General procedure GP IV – epoxide formation[6]

Potassium carbonate emulsion made according to GP III, homogenised by constant (6.5 equiv) shaking was added to the bromide-formate intermediate (1 equiv) and the mixture was stirred for 6 minutes at rt and then diluted with abs. dichloromethane (100 mL/mmol).

The solution was added to cold potassium hydrogen sulfate solution (0.5 wt %, 100 mL/mmol) and the phases were immediately separated and dried over magnesium sulfate. The aqueous phase was extracted with dichloromethane (3 × 20 mL/mmol) and the solvent was removed under reduced pressure.

3 X-ray diffraction data

Suitable crystals of Boc-d-Phe(4-NHMe)-OMe **7** for structure determination by X-Ray diffraction were grown by carefully layering cyclohexane over a saturated solution of Boc-d-Phe(4-NHMe)-OMe **7** in ethyl acetate at room temperature. The crystal was selected, coated with paratone-N oil, mounted on a glass fiber, and transferred onto the goniometer of a Rigaku SuperNova diffractometer and analysed using Cu K_α radiation. The crystal was kept at 100.0(1) K during data collection.

	Boc-d-Phe(NHMe)-OMe **7**
Empirical formula	$C_{16}H_{24}N_2O_4$
M / g·mol^{-1}	308.380
Temperature / K	100.0(1)
Crystal system	monoclinic
Space group	$P2_1$
a / Å	10.17140(16)
b / Å	6.09626(10)
c / Å	14.1657(2)
β / °	109.4582(17)
Volume / Å^3	828.21(2)
Z	2
ρ_{calc} / g·cm^{-3}	1.237
μ / mm^{-1}	0.728
F(000)	333.2
Crystal size / mm^3	0.43 × 0.25 × 0.05
Radiation / Å	Cu K_α (λ = 1.54184)
2Θ range for data collection / °	6.6 to 151.7
Index ranges	-12 ≤ h ≤ 12, -7 ≤ k ≤ 6, -17 ≤ l ≤ 17
Reflections collected	12565
Independent reflections	3138 [R_{int} = 0.0327, R_{sigma} = 0.0213]
Reflections with $I > 2\sigma(I)$	3032
Data / restraints / parameters	3138/1/273
Goodness-of-fit on F^2	1.062
Final R indexes [$I > 2\sigma(I)$]	R_1 = 0.0285, wR_2 = 0.0646
Final R indexes [all data]	R_1 = 0.0307, wR_2 = 0.0673
Largest difference peak / hole / e·Å^{-3}	0.15/-0.11
Flack parameter	-0.07(6)
CCDC number	2400694

Using Olex2[7], the structure was solved with the SHELXT[8] structure solution program using Intrinsic Phasing and refined with the olex2.refine[9] refinement package using Gauss-Newton minimisation. Refinement using NoSpherA2[10], an implementation of NOn-SPHERical atom-form-factors in Olex2. Hydrogen atoms were refined isotropically.

4 Experimental protocols

4.1 Unit B

4.1.1 H-D-Phe(4-NO$_2$)-OH 3·H$_2$O

Synthesis based on Wang *et al.*[11] A mixture of HNO_3 (65 wt %, 11.8 mL) and H_2SO_4 (95 wt %, 9.3 mL) was added slowly to a solution H-D-Phe-OH (25.0 g, 0.15 mol, 1.0 equiv) in H_2SO_4 (95 wt %, 75 mL) at 0 °C. The reaction was stirred at 0 °C for 5 h. The mixture was poured onto ice and neutralised with aqueous ammonia solution (25 wt %, 240 mL) to pH 6. The precipitate was isolated and washed with a small amount of water and acetonitrile. Recrystallisation from water (350 mL) gave H-D-Phe(4-NO$_2$)-OH **3**·H$_2$O (16.3 g, 71.4 mmol, 48 %) as beige crystalline solid.

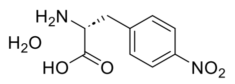

3 • H$_2$O

formula:	$C_9H_{10}N_2O_4 \bullet H_2O$
molar mass:	228.20 g/mol
yield:	16.3 g (71.4 mmol, 48%)

^{1}H-NMR (500 MHz, DMSO-d_6) δ /ppm = 8.15 (d, ^3J = 8.2 Hz, 2H, C^{Ar}**H**), 7.54 (d, ^3J = 8.3 Hz, 2H, C^{Ar}**H**), 3.47 (m, 1H, C^α**H**), 3.24 (m, 1H, C^β**H**^{A}H^B), 3.00 (dd, ^2J = 14.2 Hz, ^3J = 7.8 Hz, 1H, C^{β}H^A**H**^{B}).

The spectrum is in accordance with literature,[12] however the signals between 3.00 and 3.47 ppm are better resolved.

4.1.2 H-D-Phe(4-NO$_2$)-OMe (4)

Synthesis based on Arns *et al.*[13] H-D-Phe(4-NO$_2$)-OH **3**·H$_2$O (25.0 g, 110 mmol, 1.0 equiv) was suspended in MeOH (550 mL) and cooled to 0 °C. $SOCl_2$ (16 mL, 0.22 mol, 2.0 equiv) was added over 90 min and the mixture was refluxed for 17 h. The solvent was removed in vacuum and EtOAc (750 mL) and sat. aqueous $NaHCO_3$ (1000 mL) were added. The phases were separated, and the aqueous layer was further extracted with EtOAc (2 × 250 mL). The combined organic layers were washed with brine (500 mL) and dried over $MgSO_4$. Evaporation of the solvent gave H-D-Phe(4-NO$_2$)-OMe (**4**, 23.3 g, 104 mmol, 95%).

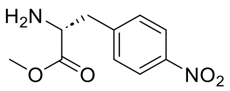

4

formula:	$C_{10}H_{12}N_2O_4$
molar mass:	224.22 g/mol
yield:	23.3 g (104 mmol, 95%)

^{1}H-NMR (500 MHz, $CDCl_3$): *δ* / ppm = 8.17 (d, ^3J = 8.2 Hz, 2 H, C^{Ar}**H**), 7.38 (d, ^3J = 8.2 Hz, 2H, C^{Ar}H), 3.76 (dd, ^3J = 7.9 Hz, ^3J = 5.5 Hz, 1H, C^α**H**), 3.72 (s, 3H, CO$_2$C**H**$_3$), 3.17 (dd, ^2J = 13.6 Hz, ^3J = 5.3 Hz, 1H, C^β**H**^{A}H^B), 2.97 (dd, ^2J = 13.6 Hz, 7.8 Hz, 1H, C^{β}H^A**H**^{B}).

The ^{1}H NMR spectrum shows all expected signals with matching multiplicities, however in all examined literature procedures towards the synthesis of L- or D-**4** no ^{1}H NMR spectrum in $CDCl_3$ was recorded.

4.1.3 Boc-D-Phe(4-NO$_2$)-OMe (5)

Synthesis based on Arns *et al.*[13] H-D-Phe(4-NO_2)-OMe (**4**, 13.9 g, 62.0 mmol, 1.0 equiv) was dissolved in a mixture of acetonitrile and H_2O (2:1, 426 mL). Et_3N (18.8 mL, 136 mmol, 2.2 equiv) and Boc_2O (18.9 g, 86.6 mmol, 1.4 equiv) were added and the mixture was stirred at rt for 22 h. The solvent was removed in vacuum and the residue was taken up in EtOAc (630 mL) and washed with sat. aqueous NH_4Cl solution (200 mL) and brine (200 mL). The organic layer was dried over $MgSO_4$. Evaporation of the solvent gave Boc-D-Phe(4-NO_2)-OMe (**5**, 17.7 g, 54.6 mmol, 88%).as a yellow solid.

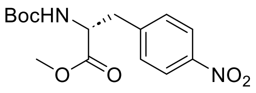

5

formula:	$C_{15}H_{20}N_2O_6$
molar mass:	324.33 g/mol
yield:	17.7 g (54.6 mmol, 88%).

TLC: R_f(PE/EtOAc, 2:1) = 0.67

^{1}H-NMR (500 MHz, $CDCl_3$): δ / ppm = 8.16 (d, ^3J = 8.7 Hz, 2H, C^{Ar}**H**), 7.32 (d, ^3J = 8.7 Hz, 2H, C^{Ar}**H**), 5.04 (d, ^3J = 8.0 Hz, 1H, N**H**), 4.64 (dd, ^2J = 14.0, ^3J = 6.0 Hz, 1H, C$^{\alpha}$**H**), 3.74 (s, 3H, CO_2C**H**$_3$), 3.27 (dd, ^2J = 13.6 Hz, ^3J = 5.9 Hz, 1H, C$^{\beta}$**H**^{A}H^B), 3.12 (dd, ^2J = 13.9 Hz, ^3J = 6.5 Hz, 1H, C$^{\beta}$**H**^{A}H^B), 1.41 (s, 9H, C(C**H**$_3$)$_3$).

The spectrum is in accordance with literature.[14]

4.1.4 Boc-D-Phe(4-NH$_2$)-OMe (6)

Methanol (300 mL) was added to Boc-D-Phe(4-NO_2)-OMe (**5**, 17.6 g, 0.054 mol, 1.0 equiv) and treated through ultrasonication. Pd/C (10 wt %, 0.58 g) was added and hydrogen was bubbled through the suspension. Complete conversion was detected by TLC after 25 h. The reaction mixture was filtered through a pad of celite and washed with methanol. The solvent was removed under reduced pressure to give Boc-D-Phe(4-NH_2)-OMe (**6**, 15.7 g, 0.053 mol, 98%) as an orange oil.

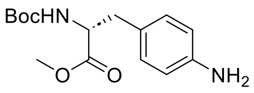

6

formula:	$C_{15}H_{22}N_2O_4$
molar mass:	294.35 g/mol
yield:	15.7 g (0.053 mol, 98%)

TLC: R_f(PE/EtOAc, 2:1) = 0.35

^{1}H-NMR (500 MHz, $CDCl_3$): δ / ppm = 6.90 (d, ^{3}J = 8.2 Hz, 2H, C^{Ar}**H**), 6.61 (d, ^{3}J = 8.3 Hz, 2H, C^{Ar}**H**), 4.94 (d, ^{3}J = 8.2 Hz, 1H, N**H**), 4.50 (ddd, ^{3}J = 6.6 Hz, ^{3}J = 6.5 Hz, ^{3}J = 6.5 Hz, 1H, C^{α}**H**), 3.70 (s, 3H, OC**H**$_3$), 3.02 - 2.92 (m, 2H, C^{β}**H**$_2$), 1.42 (s, 9H, C(C**H**$_3$)$_3$).

^{13}C-NMR (500 MHz, $CDCl_3$): δ / ppm = 172.7 (**C**O_2-CH_3), 155.3 (N**C**O_2), 145.4 (**C**^{Ar}-NH_2), 130.3 (**C**$^{Ar\text{-}2}$; **C**$^{Ar\text{-}6}$), 125.8 (**C**$^{Ar\text{-}1}$), 115.4 (**C**$^{Ar\text{-}3}$; **C**$^{Ar\text{-}5}$), 79.9 (**C**(CH_3)$_3$), 54.7 (**C**$^{\alpha}$), 52.3 (CO_2**C**H_3), 37.6 (**C**$^{\beta}$), 28.5 (C(**C**H_3)$_3$).

The NMR spectra show all expected signals with matching multiplicities, however in all examined literature procedures towards the synthesis of L- or D-**6** no ^{1}H NMR spectrum in $CDCl_3$ was recorded.

4.1.5 Boc-D-Phe(4-NHMe)-OMe (7)

Method A:

Boc-D-Phe(4-NH_2)-OMe (**6**, 1.00 g, 3.40 mmol, 1.0 equiv) was dissolved in methanol (10 mL) and formalin (37 wt %, 0.26 mL, 3.4 mmol, 1.0 equiv) was added. The mixture was stirred at rt for 10 min. $NaBH_3CN$ (322 mg, 5.12 mmol, 1.5 equiv) in methanol (20 mL) was added to the suspension. The mixture was stirred at rt for 20 min, while the pH was adjusted to 7 by addition of acetic acid continuously. The suspension was filtered through a pad of celite and washed with methanol. The solvent was removed under reduced pressure and the residue was taken up in EtOAc (100 mL) and washed with sat. aqueous $NaHCO_3$ solution (120 mL). The aqueous layer was extracted with EtOAc (3 × 100 mL). The combined organic layers were dried over $MgSO_4$, and the solvent was removed under reduced pressure to give a crude product (1.1 g), which was divided into 4 parts and purified using automated flash-chromatography (gradient PE/EtOAc, 0 to 100 %, SiO_2-aminopropyl column, 12 g) Boc-D-Phe(4-NHMe)-OMe (**7**, 386 mg, 1.25 mmol, 37%) was obtained as pale-brown solid.

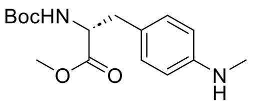

7

formula: $C_{16}H_{24}N_2O_4$

molar mass: 308.38 g/mol

yield: 386 mg (1.25 mmol, 37%).

Method B:

Synthesis based on Rhee *et al.*[15] Pd/C (10 wt %, 199 mg, 190 µmol, 0.1 equiv) was suspended in MeOH (12 mL) and a solution of ammonium formate (580 mg, 9.2 mmol, 5.4 equiv) in H_2O (1.3 mL) was added and stirred at rt for 1 min. Boc-D-Phe(4-NH_2)-OMe (**6**, 0.53 g, 1.7 mmol, 1.0 equiv) was added as solid, followed by by formalin (37 wt %, 130 µL, 1.7 mmol, 1.0 equiv). The reaction was stirred for 20 min at rt, filtrated over a pad of celite (2 cm × 1.5 cm) and eluted with MeOH (25 mL). The solvent was evaporated in vacuum. The residue

was dissolved in DCM (25 mL) and washed with brine (10 mL). The organic layer was dried over $MgSO_4$ and evaporated in vacuum. Automated column chromatography (25 g Büchi-Ecoflex silica) gave Boc-D-Phe(4-NHMe)-OMe (**7**, 187 mg, 0.61 mmol, 35%) as pale-brown solid**.**

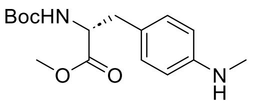

7

formula:	$C_{16}H_{24}N_2O_4$
molar mass:	308.38 g/mol
yield:	187 mg (0.61 mmol, 35%).

TLC: R_f(PE/EtOAc, 2:1) = 0.53

^{1}H-NMR (500 MHz, $CDCl_3$) δ / ppm = 6.93 (d, ^3J = 8.1 Hz, 2H, C^{ar-2}-**H**, C^{ar-6}-**H**), 6.54 (d, ^3J = 8.4 Hz, 2H, C^{ar-3}-**H**, C^{ar-5}-**H**), 4.94 (d, ^3J = 7.4 Hz, 1H, N**H**), 4.50 (ddd, ^3J = 6.2 Hz, ^3J = 6.0 Hz, ^3J = 6.0 Hz, 1H, C^{α}**H**), 3.71 (s, 3H, CO_2CH_3), 3.03 – 2.93 (m, 2H, $C^{\beta}H_2$), 2.82 (s, 3H, C^{Ar}-NH(CH_3)), 1.42 (s, 9H, Boc-CH_3).

The ^{1}H NMR spectrum shows all expected resonances with matching multiplicities, however some signals, e.g. of the NCH_3 group, are slightly shifted in comparison to literature reports of L-**7**,[16] likely due to a different degree of protonation.

4.1.6 Boc-D-Phe(4-NMe$_2$)-OMe (8)

Boc-D-Phe(4-NH_2)-OMe (**6**, 2.00 g, 6.79 mmol, 1.0 equiv) was dissolved in acetonitrile (25 mL) and $NaBH_3CN$ (700 mg, 10.9 mmol, 1.6 equiv) and formalin (37 wt %, 2.5 mL, 34.0 mmol, 5.0 equiv) were added. The reaction was stirred at rt for 55 min, while the pH was kept neutral by constant addition of glacial AcOH. The mixture was filtrated over a pad of celite and eluted with acetonitrile. Purification via column chromatography gave Boc-D-Phe(4-NMe_2)-OMe (**8**, 1.33 g, 4.13 mmol, 61%) as orange oil.

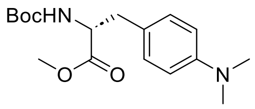

8

formula:	$C_{17}H_{26}N_2O_4$
molar mass:	322.41 g/mol
yield:	1.33 g (4.13 mmol, 61%).

TLC: R_f(PE/EtOAc, 2:1) = 0.42

R_f(PE/EtOAc, 5:1) = 0.19

**HPLC-MS** (ESI +):	*m/z* (found):	323.2158,	t_R = 6.8 min
	m/z (calc.):	323.1965	= $[M+H]^+$ = $(C_{17}H_{27}N_2O_4)^+$

^{1}H-NMR (500 MHz, $CDCl_3$) δ /ppm = 6.98 (d, ^3J = 8.2 Hz, 2H, C^{ar-2}**H**, C^{ar-6}**H**), 6.67 (d, ^3J = 8.3 Hz, 2H, C^{ar-3}**H**, C^{ar-5}**H**), 4.94 (d, ^3J = 8.3 Hz, 1H, C^{α}N**H**), 4.51 (ddd, ^3J = 5.7 Hz, ^3J =

5.7 Hz, ^3J = 5.6 Hz, 1H, C^{α}**H**), 3.72 (s, 3H, CO_2C**H_3**) 3.01 – 2.97 (m, 2H, C^{β}**H_2**), 2.92 (s, 6H, Ar-N(C**H_3**)$_2$), 1.42 (s, 9H, Boc-C**H_3**).

^{13}C-NMR (126 MHz, $CDCl_3$) δ /ppm = 172.8 (**C**O_2CH_3), 155.3 (N**C**O_2), 149.8 (**C**$^{ar\text{-}4}$-NH_2), 130.1 (ar-**C**^{ar,2} and **C**$^{ar\text{-}6}$), 123.6 (**C**$^{ar\text{-}1}$), 112.9 (**C**$^{ar\text{-}3}$ and **C**$^{ar\text{-}5}$), 79.9 (**C**$(CH_3)_3$), 54.8 (**C**$^{\alpha}$), 52.3 (CO_2**C**H_3), 40.8 (N(**C**H_3)$_2$), 38.1 (**C**$^{\beta}$), 28.5 (C(**C**H_3)$_3$).

4.1.7 Boc-D-Phe(3-Cl)(4-NHMe)-OMe (9)

Synthesis based on Patel *et al.*[17] Boc-D-Phe(4-NHMe)-OMe (**7**, 509 mg, 1.65 mmol, 1 equiv) was dissolved in acetonitrile (70 mL) under argon-atmosphere. *N*-Chlorosuccinimide (270 mg, 2.02 mmol, 1.2 equiv) was added and the mixture was refluxed overnight. Water (30 mL) was added, and the acetonitrile was removed under reduced pressure. Brine (40 mL), water (20 mL) and EtOAc were added, and the phases were separated. The aqueous layer was further extracted with EtOAc (3 × 100 mL). The combined organic layers were washed with brine (100 mL) and dried over $MgSO_4$. The solvent was removed under reduced pressure and the crude product was purified by column-chromatography (PE/EtOAc, 4:1, 20 cm × 4 cm) to give Boc-D-Phe(3-Cl)(4-NHMe)-OMe (**9**, 438 mg, 1.28 mmol, 77%) as an off-white solid.

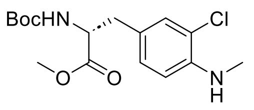

9

formula:	$C_{16}H_{23}ClN_2O_4$
molar mass:	342.82 g/mol
yield:	438 mg (1.28 mmol, 77%).

TLC: R_f (PE/EtOAc, 4:1) = 0.26

R_f (PE/EtOAc, 2:1) = 0.45

^{1}H-NMR (500 MHz, $CDCl_3$) δ / ppm = 7.01 (br s , 1H, $C^{ar\text{-}2}$**H**), 6.91 (d, ^3J = 8.2 Hz, 1H, $C^{ar\text{-}6}$**H**), 6.57 (d, 1H, ^3J = 8.2 Hz, $C^{ar\text{-}5}$**H**), 4.96 (d, ^3J = 8.3 Hz, 1H, C^{α}N**H**), 4.50 (ddd, ^3J = 6.3 Hz, ^3J = 6.0 Hz, ^3J = 6.0 Hz, 1H, C^{α}**H**), 3.72 (s, 3H, CO_2C**H_3**), 2.99 (dd, ^2J = 12.2 Hz, ^3J = 7.6 Hz, 1H, C^{β}**H**^{A}H^B), 2.93 (dd, ^2J = 14.1 Hz, ^3J = 5.7 Hz, 1H, $C^{\beta}H^A$**H**^{B}), 2.88 (s, 3H, NHC**H_3**), 1.43 (s, 9H, C(C**H_3**)$_3$).

^{13}C-NMR (126 MHz, $CDCl_3$) δ / ppm = 172.3 (**C**O_2CH_3), 155.2 (N**C**$O_2C(CH_3)_3$), 144.0 (**C**^{ar,4}), 129.9 (**C**^{ar,2}), 128.8 (**C**^{ar,6}), 124.7 (**C**^{ar,1}), 119.2 (**C**^{ar,3}), 110.9 (**C**^{ar,5}), 80.0 (**C**$(CH_3)_3$), 54.9 (**C**$^{\alpha}$), 52.3 (CO_2**C**H_3), 37.3 (**C**$^{\beta}$), 30.6 (Ar-NH**C**H_3), 28.4 (C(**C**H_3)$_3$).

4.1.8 Boc-D-Phe(3-Cl)(4-NMeAlloc)-OMe (10)

Boc-D-Phe(3-Cl)(4-NHMe)-OMe (**9**, 545 mg, 1.59 mmol, 1.0 equiv) was dissolved in chloroform (36 mL) and 1 wt % aqueous $NaHCO_3$ solution (18 mL) was added. AllocCl (339 µL, 3.18 mmol, 2.0 equiv) was injected into the organic layer by cannula. The mixture was stirred at rt for 2 h and additional AllocCl (339 µL, 3.18 mmol, 2.0 equiv) was injected into the

organic phase by cannula. The mixture was stirred at rt for 3 h and water (50 mL) and dichloromethane (50 mL) were added. The phases were separated, and the aqueous phase was further extracted with dichloromethane (2 × 50 mL). The combined organic phases were dried over $MgSO_4$, and the solvent was removed under reduced pressure. Purification by column-chromatography (PE/EtOAc, 2:1) to give Boc-D-Phe(3-Cl)(4-NMeAlloc)-OMe (**10**, 603 mg, 1.41 mmol, 89%) as an off-white solid.

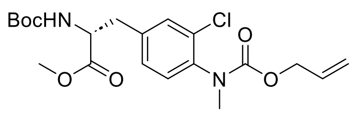

10

formula:	$C_{20}H_{27}ClN_2O_6$
molar mass:	426.89 g/mol
yield:	603 mg (1.41 mmol, 89%).

TLC: R_f (PE/EtOAc, 2:1) = 0.32

^{1}H-NMR (600 MHz, DMSO-d_6, 363 K) δ / ppm = 7.41 (d, ^4J = 1.9 Hz, 1H, C^{ar-2}-**H**), 7.32 (d, ^3J = 7.9 Hz, 1H, C^{ar-5}-**H**), 7.24 (dd, 1H, ^3J = 8.0 Hz, ^4J = 2.0 Hz, C^{ar-6}-**H**), 6.92 (br s, 1H, N**H**), 5.85 (m, 1H, C**H**=CH_2), 5.23-5.08 (m, 2H, Alloc CH=C**H**$_2$), 4.52 (s (broad), 2H, C**H**$_2$-CH=CH_2), 4.28 (m, 1H, C^{α}**H**), 3.65 (s, 3H, CO_2C**H**$_3$), 3.12 (s, 3H, ar-NHC**H**$_3$), 3.07 (dd, ^2J = 14.1 Hz, ^3J = 5.2 Hz, 1H. C^{β}**H**^{A}H^B), 2.92 (dd, ^2J = 14.1 Hz, ^3J = 9.8 Hz, 1H, $C^{\beta}H^A$**H**^{B}), 1.34 (s, 9H, C(C**H**$_3$)$_3$).

^{13}C-NMR (151 MHz, DMSO-d_6, 363 K) δ / ppm = 171.5 (**C**O_2CH_3), 154.6 (N**C**$O_2C(CH_3)_3$), 153.9 (Alloc, **C**(=O)), 138.7 (**C**$^{ar-1}$), 138.1 (**C**$^{ar-4}$), 132.7 (**C**H=CH_2), 130.8 (**C**$^{ar-3}$), 130.0 (**C**$^{ar-2}$), 128.8 (**C**$^{ar-5}$), 128.5 (**C**$^{ar-6}$), 116.3 (CH=**C**H_2), 78.1 (**C**$(CH_3)_3$), 65.1 (**C**H_2CH=CH_2), 54.3 (**C**$^{\alpha}$), 51.2 (CO_2**C**H_3), 36.4 (ar-NH**C**H_3), 35.6 (**C**$^{\beta}$), 27.7 (C(**C**H_3)$_3$).

4.1.9 Boc-D-Phe(3-Cl)(4-NMeAlloc)-OH (11)

Boc-D-Phe(3-Cl)(4-NMeAlloc)-OMe (**10**, 572 mg, 1.34 mmol, 1.0 equiv) was dissolved in THF (6.7 mL) and methanol (6.7 mL) and was chilled to 0 °C. A solution of lithium hydroxide monohydrate (84 mg, 2.0 mmol, 1.5 equiv) in water (6.7 mL) was added at 0 °C and the mixture was stirred for 3 h while slowly warming to rt. EtOAc (50 mL) and 2.5 wt % aqueous $KHSO_4$ solution (20 mL) were added and the phases were separated. The aqueous layer was further extracted with EtOAc (2 × 50 mL). The combined organic layers were washed with brine (30 mL) and dried over $MgSO_4$. Evaporation of the solvent under reduced pressure gave Boc-D-Phe(3-Cl)(4-NMeAlloc)-OH (**11**, 547 mg, 1.32 mmol, 99%), which was used in the next reaction without further purification.

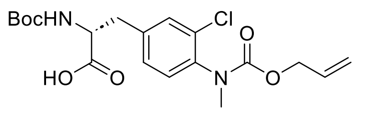

11

formula: $C_{19}H_{25}ClN_2O_6$

molar mass: 412.87 g/mol

yield: 547 mg (1.32 mmol, 99%).

HPLC-MS (ESI +): *m/z* (found) 357.0856, t_R = 8.7 min.
m/z (calc.) 357.0848 = [M-*t*Bu+H]$^+$ ($C_{15}H_{18}ClN_2O_6^+$).

4.1.10 Acryloyl-D-Phe(4-NMe$_2$)-OMe (12)

Boc-D-Phe(4-NMe$_2$)-OMe (**8**, 990 mg, 3.07 mmol, 1.0 equiv) was dissolved in DCM (9 mL) and cooled to 0 °C. TFA (6 mL) was added, and the solution was stirred at 0 °C for 1.5 h. The mixture was diluted with DCM (20 mL) and sat. aqueous $NaHCO_3$ solution was added until the aqueous layer showed a pH of 8. The phases were separated, and the aqueous layer was extracted with DCM (3 × 25 mL). The combined organic layers were washed with sat. aqueous $NaHCO_3$ solution (50 mL), dried over $MgSO_4$ and evaporated in vacuum. Under inert conditions the residue was dissolved in abs. DCM (15 mL) and cooled to 0 °C. NEt_3 (0.64 mL, 4.6 mmol, 1.5 equiv) and acryloyl chloride (0.26 mL, 3.1 mmol, 1.0 equiv were added dropwise at 0 °C and the reaction was stirred in darkness for 19 h at 0 °C while slowly warming up to rt. The solvent was evaporated. Purification by column chromatography gave acryloyl-D-Phe(4-NMe$_2$)-OMe (**12**, 534 mg, 1.93 mmol, 63%) as colourless crystalline solid.

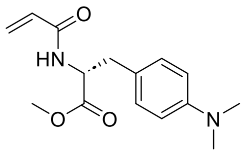

12

formula: $C_{15}H_{20}N_2O_3$

molar mass: 276.34 g/mol

yield: 534 mg (1.93 mmol, 63%).

^{1}H-NMR (600 MHz, $CDCl_3$) δ / ppm = 6.94 (d, ^3J = 8.6 Hz, 2H, C^{ar-2}**H**, C^{ar-6}**H**), 6.65 (d, ^3J = 8.4 Hz, 2H, C^{ar-3}**H**, C^{ar-5}**H**), 6.29 (dd, ^3J = 17.0 Hz, ^2J = 1.3 Hz, 1H, CH=C**H**$^{\mathbf{A}}$H^B), 6.09 (dd, ^3J = 17.0 Hz, ^3J = 10.3 Hz, 1H, C**H**=CH^{A}H^B), 5.98 (br d, ^3J = 7.1 Hz, 1H, C$^\alpha$N**H**), 5.66 (dd, ^3J = 10.3 Hz, ^2J = 1.3 Hz, 1H, CH=CH$^{\mathbf{A}}$**H**^{B}), 4.91 (ddd, ^3J = 7.7 Hz, ^3J = 5.4 Hz, ^3J = 5.4 Hz, 1H, C$^\alpha$**H**), 3.75 (s, 3H, CO_2C**H**$_3$), 3.08 (d, ^3J = 5.4 Hz, 2H, C$^\beta$**H**$_2$), 2.92 (s, 3H, C^{Ar}-NH(C**H**$_3$)$_2$).

^{13}C-NMR (151 MHz, $CDCl_3$) δ / ppm = 172.3 (**C**O$_2$CH$_3$), 165.0 (N**C**(=O)), 149.9 (**C**^{ar,4}), 130.6 (**C**H=CH$_2$), 130.1 (**C**^{ar,2} and **C**^{ar,6}), 127.2 (CH=**C**H$_2$), 123.1 (**C**$^{ar-1}$), 112.9 (**C**^{ar,3} and **C**^{ar,5}), 53.5 (**C**$^\alpha$), 52.5 (CO$_2$**C**H$_3$), 40.7 (Ar-NH(**C**H$_3$)$_2$), 36.9 (**C**$^\beta$).

HPLC-MS (ESI +): *m/z* (found) 277.1752, t_R = 3.8 min.
m/z (calc.) 277.1547 = [M+H]$^+$ ($C_{15}H_{21}N_2O_3^+$).

4.1.11 Acryloyl-D-Phe(4-NMe$_2$)-OH (13)

Acryloyl-D-Phe(4-NMe$_2$)-OMe (**12**, 506 mg, 1.83 mmol, 1.0 equiv) was dissolved in THF (9.5 mL) and methanol (9.5 mL) and was chilled to 0 °C. A solution of lithium hydroxide monohydrate (80 mg, 1.9 mmol, 1.0 equiv) in water (9.5 mL) was added at 0 °C and the mixture was stirred at 0 °C for 3.5 h. The pH was adjusted to 3 to 4 by addition of hydrochloric acid (1 M). Brine (10 mL) was added, and the mixture was extracted with EtOAc/iPrOH (9:1, 5 × 50 mL). The aqueous layer was again adjusted to pH 4 using $NaHCO_3$, hydrochloric acid (1 M) and H_2O (50 mL) and extracted with EtOAc/iPrOH (9:1, 3 × 50 mL). The combined organic layers were washed with H_2O (100 mL) and dried over $MgSO_4$. Evaporation of the solvent under reduced pressure gave acryloyl-D-Phe(4-NMe$_2$)-OH (**13**) as a mixture with its starting material **12** and iPrOH (591 mg, molar ratio: **13**:**12** = 82:18, corresponding to 393 mg, 1.50 mmol, 82% of **13**) as an orange-brown oil which was used in the next step without further purification.

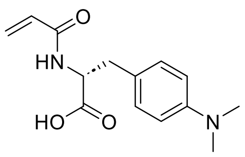

13

formula:	$C_{14}H_{18}N_2O_3$
molar mass:	262.31 g/mol
yield:	391 mg, 1.50 mmol, 82%

Data of **13**:

**HPLC-MS** (ESI+):	*m/z* (found)	263.1585, t_R = 2.5 min.
	m/z (calc.)	263.1390 = $[M+H]^+$ ($C_{14}H_{19}N_2O_3^+$)

Data of **13**, NMR contains approx. 18 mol % starting material **12**.

^{1}H-NMR (500 MHz, CD_3OD) δ / ppm = 7.08 (d, ^3J = 8.2 Hz, 2H, C^{ar-2}**H**, C^{ar-6}**H**), 6.65 (d, ^3J = 8.2 Hz, 2H, C^{ar-3}**H**, C^{ar-5}**H**), 6.27 (dd, ^3J = 17.0 Hz, ^3J = 10.1 Hz, 1H, C**H**=CH^{A}H^B), 6.17 (d, ^3J = 17.0 Hz, 1H, CH=C**H^A**H^B), 5.64 (dd, ^3J = 10.0 Hz, ^2J = 1.3 Hz, 1H, CH=CH^A**H^B**), 4.66 (dd, ^3J = 8.6 Hz, ^3J = 5.6 Hz, 1H, C^{α}**H**), 3.12 (d, ^2J = 14.1 Hz, ^3J = 5.2 Hz, 1H, C^{β}**H^A**H^B), 2.91 (m, 1H, C^{β}**H^A**H^B), 2.88 (s, 3H, C^{Ar}-NH(C**H$_3$**)$_2$).

4.2 Fragments CDA

4.2.1 Fmoc-uC-uD-O*t*-Bu 16

Unit D **14**[4] (0.57 g, 3.03 mmol, 1.0 equiv), Fmoc-uC-OH (**15**)[2,3] (1.53 g, 4.51 mmol, 1.5 equiv) and NEt_3 (0.63 mL, 4.6 mmol, 1.5 equiv) were dissolved in DCM (12.5 mL) and DMAP (68 mg, 0.55 mmol, 0.2 equiv) was added at 0 °C. EDC·HCl (1.15 g, 6.01 mmol, 2.0 equiv) was added slowly at 0 °C over the course 8 min and stirred for 22 h while warming up to rt. The reaction mixture was diluted with Et_2O/EtOAc (1:1, 115 mL) and H_2O (28.5 mL). The phases were separated, and the aqueous layer was extracted with Et_2O/EtOAc (1:1, 3 × 12 mL). The combined organic layers were washed with aqueous $KHSO_4$ solution (5 wt %, 17 mL), saturated aqueous $NaHCO_3$ solution (17 mL) and brine (17 mL), dried over $MgSO_4$ and evaporated. Column chromatography (PE/EtOAc, 8:1) gave Fmoc-uC-uD-O*t*-Bu **16** (922 mg, 1.81 mmol, 60%) as pale-yellow oil.

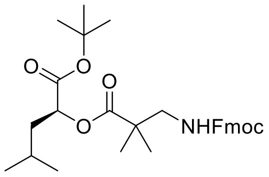

16

formula:	$C_{30}H_{39}NO_6$
molar mass:	509.64 g/mol
yield:	922 mg (1.81 mmol, 60%)

TLC: R_f (PE/EtOAc 8:1) = 0.57.

^{1}H NMR (500 MHz, $CDCl_3$): δ /ppm = 7.76 (d, ^3J = 7.5 Hz, 2H, Fmoc-C^{ar}**H**), 7.65 (dd, ^3J = 11.1 Hz, ^3J = 7.6 Hz, 2H, Fmoc-C^{ar}**H**), 7.39 (dd, ^3J = 7.5 Hz, ^3J = 7.5 Hz, 2H, Fmoc-C^{ar}**H**), 7.32 – 7.28 (m, 2H, Fmoc-C^{ar}**H**), 6.28 (t, ^3J = 6.8 Hz, 1H, N**H**), 5.01 (dd, ^3J = 9.7 Hz, ^3J = 3.7 Hz, 1H, uD-C^{α}**H**), 4.38 (m, 1H, Fmoc, CHC$\mathbf{H}^AH^B$), 4.30 (dd, ^3J = 10.6 Hz, ^3J = 7.4 Hz, 1H, Fmoc, CHC$H^A\mathbf{H}^B$), 4.22 (dd, ^3J = 7.6 Hz, ^3J = 7.6 Hz, 1H, Fmoc, C**H**CH^AH^B), 3.42 (d, ^3J = 6.6 Hz, 2H, uC-$C^{\beta}\mathbf{H_2}$), 1.88 – 1.73 (m, 2H, uD-$C^{\beta}\mathbf{H}^AH^B$ and uD-C^{γ}**H**), 1.66 (m, 1H, uD-$C^{\beta}H^A\mathbf{H}^B$), 1.51 (s, 9H, $C(C\mathbf{H_3})_3$), 1.26 (s, 3H, uC-$C^{\alpha}(C\mathbf{H_3})^A(CH_3)^B$), 1.24 (s, 3H, uC-$C^{\alpha}(CH_3)^A(C\mathbf{H_3})^B$), 0.98 (d, ^3J = 6.3 Hz, 3H, uD-$C^{\gamma}(C\mathbf{H_3})^A(CH_3)^B$), 0.95 (d, ^3J = 6.3 Hz, 3H, uD-$C^{\gamma}(C\mathbf{H_3})^A(CH_3)^B$).

4.2.2 Fmoc-uC-uD-OH 17

Fmoc-uC-uD-O*t*-Bu **16** (1.15 g, 2.26 mmol, 1.0 equiv) was dissolved in DCM (15 mL). TFA (10 mL) was added dropwise at 0 °C and the solution was stirred at 0 °C for 4 h. The solvents were evaporated. Co-evaporation of the residue with toluene (3 × 6 mL) gave crude Fmoc-uC-uD-OH **17** (1.07 g, quant.) as a colourless oil, which was used in the next reaction without further purification.

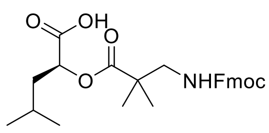

17

formula: $C_{26}H_{31}NO_6$

molar mass: 453.54 g/mol

yield: 1.07 g (quant.)

4.2.3 Fmoc-uC-uD-uA-CO₂*t*-Bu 20

Synthesis based on Figueras *et al.*[18] Under inert conditions unit A **18** (662 mg, 1.75 mmol, 1.0 equiv) and crude Fmoc-uC-uD-OH **17** (738 mg, 1.75 mmol, 1.0 equiv) was dissolved in dry THF (18 mL). Subsequently DMAP (54 mg, 0.44 mmol, 0.25 equiv), NEt_3 (0.49 mL, 3.5 mmol, 2.0 equiv) and 2,4,6-trichlorobenzoyl chloride (0.55 mL, 3.5 mmol, 2.0 equiv) were added slowly at 0 °C. The reaction mixture was stirred for 3 h while warming up to rt. An aqueous solution of citric acid (10 wt %, 50 mL) was added. The mixture was extracted with EtOAc (3 × 50 mL). The combined organic layers were washed with saturated aqueous $NaHCO_3$ solution (50 mL) and brine (50 mL). The organic layer was dried over $MgSO_4$, and the solvent was evaporated under reduced pressure. Column chromatography (PE/EtOAc, 5:1) gave Fmoc-uC-uD-uA-CO_2*t*-Bu **20** (1.09 g, 1.34 mmol, 77%) as colourless oil.

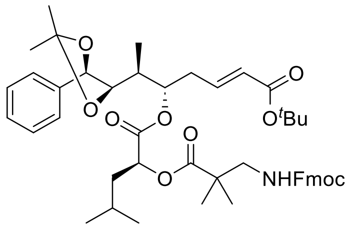

20

formula: $C_{48}H_{61}NO_{10}$

molar mass: 812.01 g/mol

yield: 1.09 g (1.34 mmol, 77%)

TLC: R_f (PE/EtOAc 5:1) = 0.35.

¹H NMR (500 MHz, $CDCl_3$, main rotamer): δ/ppm = 7.76 (d, ^3J = 7.6 Hz, 2H, Fmoc-C^{ar}**H**), 7.64 (dd, ^3J = 12.3 Hz, ^3J = 7.5 Hz, 1H, Fmoc-C^{ar}**H**), 7.39 (t, ^3J = 7.5 Hz, 2H, Fmoc-C^{ar}**H**), 7.37 – 7.27 (m, 7H, Fmoc-C^{ar}**H** and uA-C^{ar}**H**), 6.58 (ddd, ^3J = 15.1 Hz, ^3J = 7.3 Hz, ^3J =7.3 Hz, 1H, uA-C^{β}**H**), 5.97 (t, ^3J = 6.7 Hz, 1H, uC-N**H**), 5.57 (dd, ^3J = 15.7 Hz, ^4J = 1.4 Hz, 1H, uA-C^{α}**H**), 5.04 (ddd, ^3J = 6.8 Hz, ^3J = 6.8 Hz, ^3J = 4.6 Hz, 1H, uA-C^{δ}**H**),5.00 (dd, ^3J = 10.1 Hz, ^3J = 3.5 Hz, 1H, uD-C^{α}**H**), 4.70 (d, ^3J = 8.8 Hz, 1H, uA-C^{η}**H**), 4.36 (dd, ^2J = 10.3 Hz, ^3J = 7.6 Hz, 1H, Fmoc, CHC**H**^{A}H^B), 4.33 (dd, ^2J = 10.0 Hz, ^3J = 7.0 Hz, 1H, Fmoc, CHCH^A**H**^{B}), 4.23 (dd, ^3J = 7.5 Hz, ^3J = 7.5 Hz, 1H, Fmoc, C**H**CH^{A}H^B), 3.83 (dd, ^3J = 8.8 Hz, ^3J = 2.5 Hz, 1H, uA-C^{ζ}**H**), 3.36 (d, ^3J = 6.5 Hz, 2H, uC-C^{β}**H₂**), 2.51 – 2.21 (m, 2H, uA-C^{γ}**H**^{A}**H**^{B}), 1.94 (qdd, ^3J = 6.6 Hz, ^3J = 6.6 Hz, ^3J = 2.0 Hz, 1H, uA-C^{ε}**H**), 1.82 – 1.68 (m, 2H), uD-C^{γ}**H** and uD-C^{β}**H**^{A}H^B), 1.62 – 1.54 (m, 1H, uD-C^{β}H^A**H**^{B}), 1.52 (s, 3H, uA-C(C**H**₃)^A(CH₃)^B), 1.46/1.45 (2s, 12H, uA-C(CH₃)^A(C**H**₃)^B and uA-CO_2C(C**H**₃)₃), 1.22 (s, 3H, uC-C^{α}(C**H**₃)^A(CH₃)^B), 1.20 (s, 3H, uC-C^{α}(CH₃)^A(C**H**₃)^B), 1.09 (d, ^3J = 6.9 Hz, 1H, uA-C^{ε}C**H**₃), 0.95 (d, ^3J = 6.4 Hz, 1H, uD-C^{γ}(C**H**₃)^A(CH₃)^B), 0.90 (d, ^3J = 6.5 Hz, 1H, uD-C^{γ}(CH₃)^A(C**H**₃)^B).

4.2.4 Fmoc-uC-uD-uA-H 21

Synthesis based on Figueras *et al.*[18] Under inert conditions unit A **19** (647 mg, 2.34 mmol, 1.0 equiv) and Fmoc-uC-uD-OH **17** (1.22 g, 2.69 mmol, 1.2 equiv) were dissolved in dry THF (25 mL). Subsequently DMAP (72 mg, 0.59 mmol, 0.25 equiv), NEt_3 (0.65 mL, 4.7 mmol, 2.0 equiv) and 2,4,6-trichlorobenzoyl chloride (0.73 mL, 4.7 mmol, 2.0 equiv) were added slowly at 0 °C. The reaction mixture was stirred for 3 h at 0 °C. An aqueous solution of citric acid (10 wt %, 60 mL) was added. The mixture was extracted with EtOAc (3 × 60 mL). The combined organic layers were washed with saturated aqueous $NaHCO_3$ solution (60 mL) and brine (60 mL). The organic layer was dried over $MgSO_4$, and the solvent was evaporated under reduced pressure. Column chromatography (PE/EtOAc, 9:1) gave Fmoc-uC-uD-uA-H **21** (1.47 g, 2.06 mmol, 88%) as a pale-yellow oil.

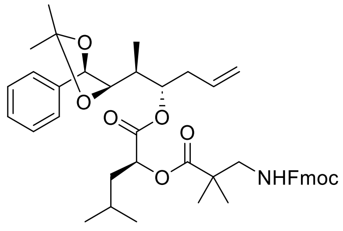

21

formula:	$C_{43}H_{53}NO_8$
Molar mass:	711.90 g/mol
yield:	1.47 g (2.06 mmol, 88%)

TLC: R_f (PE /EtOAc, 9:1) = 0.21.

^{1}H NMR (500 MHz, $CDCl_3$) δ / ppm = 7.76 (d, ^3J = 7.5 Hz, 2H, Fmoc-C^{ar}**H**), 7.64 (dd, ^3J = 11.9 Hz, ^3J = 7.5 Hz, 2H, Fmoc-C^{ar}**H**), 7.42–7.27 (m, 9H, Fmoc-C^{ar}**H** and uA-C^{ar}**H**), 6.16 (s, 1H, uC-N**H**), 5.53 (dddd, ^3J = 17.1 Hz, ^3J = 10.2 Hz, ^3J = 7.0 Hz, ^3J = 7.0 Hz, uA-C**H**=CH$_2$), 5.06–4.99 (m, 2H, uD-C$^\alpha$-**H**, uA-C$^\delta$**H**), 4.96 (dd, ^3J = 10.3 Hz, ^2J = 1.6 Hz, 1H, uA-CH=C**H**^{A}H^B), 4.88 (dd, ^3J = 17.1 Hz, ^2J = 1.8 Hz, 1H, uA-CH=CH^A**H**^{B}), 4.71 (d, ^3J = 8.8 Hz, 1H, uA-C$^\eta$**H**), 4.38 (dd, ^2J = 10.4 Hz, ^3J = 7.7 Hz, 1H, Fmoc, CHC**H**^{A}H^B), 4.32 (dd, ^3J = 10.5 Hz, ^3J = 7.4 Hz, 1H, Fmoc, CHCH^A**H**^{B}), 4.23 (dd, ^3J = 7.5 Hz, ^3J = 7.5 Hz, 1H, Fmoc, C**H**CH^{A}H^B), 3.86 (dd, ^3J = 8.8 Hz, ^3J = 2.5 Hz, 1H, uA-C$^\zeta$**H**), 3.39 (d, ^3J = 6.6 Hz, 2H, uC-C$^\beta$**H$_2$**), 2.33 – 2.19 (m, 2H, uA-C**H**^{A}**H**^{B}-CH=CH$_2$), 1.93 (m, 1H, uA-C$^\varepsilon$**H**), 1.82 – 1.69 (m, 2H, uD-C$^\beta$**H**^{A}**H**^{B}), 1.61 (m, 1H, uD-C$^\gamma$**H**), 1.53 (s, 3H, uA-C(C**H$_3$**)^A(CH$_3$)^B), 1.46 (s, 3H, uA-C(CH$_3$)^A(C**H$_3$**)^B), 1.23 (s, 3H, uC-C(C**H$_3$**)^A(CH$_3$)^B), 1.21 (s, 3H, uC-C(CH$_3$)^A(C**H$_3$**)^B), 1.10 (d, ^3J = 7.0 Hz, 3H, uA-C$^\varepsilon$C**H$_3$**), 0.96 (d, ^3J = 6.5 Hz, 1H, uD-C$^\gamma$(C**H$_3$**)^A(CH$_3$)^B), 0.90 (d, ^3J = 6.4 Hz, 3H, uD-C$^\gamma$(CH$_3$)^A(C**H$_3$**)^B).

4.3 Synthesis of cryptophycin-uB[D-Phe(3-Cl)(4-NHMe)] 1

4.3.1 uB[D-Phe(3-Cl)(4-NMeAlloc)]-modified *seco*-cryptophycin 22

Fmoc-uC-uD-uA-CO_2*t*-Bu **20** (929 mg, 1.14 mmol, 1.0 equiv) was dissolved in DMF (8 mL) and piperidine (0.57 mL, 5.7 mmol, 5.0 equiv) was added dropwise. The mixture was stirred at rt for 1.5 h and the solvents were removed in high vacuum. The residue was dissolved in abs. DCM (4 mL) and was added to a solution of unit B **11** (609 mg, 1.48 mmol, 1.3 equiv) and NEt_3 (508 µL, 3.66 mmol, 3.2 equiv) in dry DCM (4 mL) at 0 °C. HOAt (248 mg, 1.82 mmol, 1.6 equiv) and EDC·HCl (351 mg, 1.83 mmol, 1.6 equiv) were added and the solution was stirred at rt for 18 h. The reaction mixture was diluted with EtOAc (150 mL) and water (150 mL) and the phases were separated. The aqueous layer was further extracted with EtOAc (150 mL), and the combined organic layers were washed with aqueous $KHSO_4$ solution (5 wt %, 100 mL) and saturated aqueous $NaHCO_3$ solution (100 mL) and dried over $MgSO_4$. The solvent was removed under reduced pressure and the residue was purified via column-chromatography (PE/EtOAc, 2:1, 2.5 cm × 25 cm) to yield *seco*-cryptophycin **22** (547 mg, 556 µmol, 49%) as colourless solid.

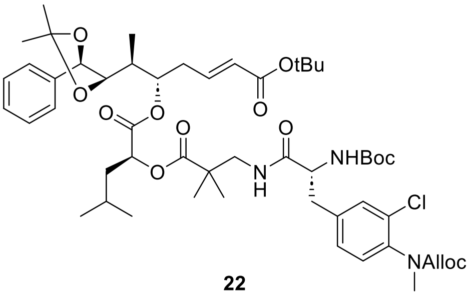

22

formula:	$C_{52}H_{74}ClN_3O_{13}$
molar mass:	984.62 g/mol
yield:	547 mg (556 µmol, 49%)

TLC: R_f (PE/EtOAc, 2:1) = 0.29

HPLC-MS (ESI +): *m/z* (found): 984.4753, 928.4753, 884.4280, 828.3660
t_R = 8.9 min (isocratic method)

m/z (calc.): 984.4983 = $[M+H]^+$ = $(C_{52}H_{75}ClN_3O_{13})^+$
928.4357 = $[M\text{-}t\text{Bu}+H]^+$ = $(C_{48}H_{67}ClN_3O_{13})^+$
884.4459 = $[M\text{-Boc}+H]^+$ = $(C_{47}H_{67}ClN_3O_{11})^+$
828.3833 = $[M\text{-}CO_2+H]^+$ = $(C_{43}H_{59}ClN_3O_{11})^+$

^{1}H NMR (600 MHz, DMSO-d_6, 363 K) δ / ppm = 7.41 – 7.31 (m, 6 H, uA-Ph, uCN**H**), 7.41 (d, ^4J = 2.0 Hz, 1H, uB-C^2**H**), 7.30 (d, ^3J = 8.1 Hz, 1H, uB-C^5**H**), 7.24 (dd, ^3J = 8.1 Hz, ^4J = 1.9 Hz, 1H, uB-C^6**H**), 6.57 (s (broad), 1H, uB-N**H**Boc), 6.50 (m, 1H, uA-C$^\beta$**H**), 5.83 (m, 1H, uB-C**H**=CH_2), 5.61 (d, ^3J = 15.6 Hz, 1H, uA-C$^\alpha$**H**), 5.20 – 5.07 (m, 2H, uB-CH=C**H**$_2$), 4.91 – 4.86 (m, 2H, uD-C$^\alpha$**H**, uA-C$^\delta$**H**), 4.73 (d, ^3J = 8.7 Hz, 1H, uA-C$^\eta$**H**), 4.51 (s (broad), 2H, uB-C**H**$_2$CH=CH_2), 4.25 (ddd, ^3J = 9.2 Hz, ^3J = 9.1 Hz, ^3J = 4.8 Hz, 1H, uB-C$^\alpha$**H**), 3.84 (dd, ^3J = 8.7 Hz, ^3J = 2.8 Hz, 1H, uA-C$^\zeta$**H**), 3.31 (dd, ^2J = 13.4 Hz, ^3J = 6.6 Hz, 1H, uC-C$^\beta$**H**^{A}H^B),

3.23 (dd, ^2J = 13.3 Hz, ^3J = 6.0 Hz, 1H, uC-C^{β}H^A**H**^{B}), 3.12 (s, 3H, uB-NC**H**$_3$), 3.00 (dd, ^2J = 14.4 Hz, ^3J = 4.7 Hz, 1H, uB-C^β**H**^{A}H^B), 2.81 (dd, ^2J = 14.0 Hz, ^3J = 9.8 Hz, 1H, uB-C^{β}H^A**H**^{B}), 2.43 – 2.31 (m, 2H, uA-C^γ**H**$_2$), 1.91 (qdd, ^3J = 6.8 Hz, ^3J = 6.8 Hz, ^3J = 2.7 Hz, 1H, uA-C^ε**H**), 1.72 – 1.64 (m, 2H, uD-C^β**H**^{A}H^B, uD-C^γ**H**), 1.57 (m, 1H, uD-C^{β}H^A**H**^{B}), 1.47 (s, 3H, uA-C(C**H**$_3$)^A(CH$_3$)^B), 1.42 (s, 3H, uA-C(CH$_3$)^A(C**H**$_3$)^B), 1.32 (s, 9H, uB-C(C**H**$_3$)$_3$), 1.08 (s, 3H, uC-C(C**H**$_3$)^A(CH$_3$)^B), 1.07 (s, 3H, uC-C(CH$_3$)^A(C**H**$_3$)^B), 1.06 (d, ^3J = 6.9 Hz, 1H, uA-C^{ε}C**H**$_3$), 0.89 (d, ^3J = 6.3 Hz, 3H, uD-C^{δ,A}**H**$_3$), 0.87 (d, ^3J = 6.4 Hz, 3H, uD-C^{δ,B}**H**$_3$).

^{13}C NMR (151 MHz, DMSO-*d*$_6$, 363 K) δ / ppm = 174.7 (uC-**C**=ON-uD), 171.0 (uB-**C**=ONH-uC), 168.8 (uD-**C**=ONH-uA), 164.0 (uA-**C**O$_2$C(CH$_3$)$_3$), 154.5 (uB-N**C**O$_2$C(CH$_3$)$_3$), 153.9 (uB-N**C**O$_2$CH$_2$CH=CH$_2$), 141.6 uA-**C**^{β}), 139.1 (uB-**C**^{1}), 137.9 (uB-**C**^{4}), 137.4 (uA-**C**^{ar,1}), 132.7 (uB-NCO$_2$CH$_2$**C**H=CH$_2$), 130.7 (uB-**C**^{3}-Cl), 129.9 (uB-**C**^{6}), 128.6 (uB-**C**^{5}), 128.5 (uB-**C**^{2}), 127.9 (uA-**C**^{ar}), 127.7 (uA-**C**^{ar}), 126.3 (uA-**C**^{ar}), 125.0 (uA-**C**^{α}), 116.2 (uB-NCO$_2$CH$_2$CH=**C**H$_2$), 107.8 (uA-**C**(CH$_3$)$_2$), 81.0 (uA-**C**^{ζ}), 79.3 (uA-**C**^{η}), 79.1 (uA-CO$_2$**C**(CH$_3$)$_3$), 77.9 (uB-NCO$_2$**C**(CH$_3$)$_3$), 74.7 (uA-**C**^{δ}), 70.4 (uD-**C**^{α}), 65.0 (uB-NCO$_2$**C**H$_2$CH=CH$_2$), 55.2 (uB-**C**^{α}), 45.6 (uC-**C**H$_2$), 42.5 (uC-**C**(CH$_3$)$_2$), 38.9 (uD-**C**^{β}), 36.6 (uB-**C**^{β}), 36.4 (uB-N**C**H$_3$), 35.3 (uA-**C**^{ε}), 32.5 (uA-**C**^{γ}), 27.6 (uB-NCO$_2$C(**C**H$_3$)$_3$), 27.4 (uA-CO$_2$C(**C**H$_3$)$_3$), 26.5 (uA-C(**C**H$_3$)^A(CH$_3$)^B), 26.5 (uA-C(CH$_3$)^A(**C**H$_3$)^B), 23.8 (uD-**C**^{γ}), 22.2 (uD-**C**^{δ,A}), 22.0 (uC-C(**C**H$_3$)^A(CH$_3$)^B), 21.9 (uC-C(CH$_3$)^A(**C**H$_3$)^B), 21.1(uD-**C**^{δ,B}), 8.6 (uA-C^ε**C**H$_3$).

4.3.2 Modified cryptophycin-uA[diol]-uB[D-Phe(3-Cl)(4-NMeAlloc)] 24

Fully protected *seco*-cryptophycin **22** (468 mg, 475 µmol, 1.0 equiv) was dissolved in DCM (5.2 mL) and water (1.1 mL). The mixture was cooled to 0 °C, TFA (5.2 mL) was added, and it was stirred for 2.5 h while slowly warming to rt. The solvent was removed, and the residue was dried in HV for two weeks over KOH. The crude deprotected cryptophycin intermediate was dissolved in DMF (25 mL). HATU (235 mg, 618 µmol, 1.3 equiv) was dissolved in DMF (25 mL). Both solutions were combined slowly with a double-tipped syringe pump (0.023 mL/min, each) to a solution of HOAt (16 mg, 0.120 mmol, 0.25 equiv) in DMF (25 mL) at 0 °C. After complete addition, the mixture was stirred at 0 °C for 2 h and at rt for 20 min. The solvent was removed, and the residue was dissolved in EtOAc (150 mL) and washed with saturated aqueous $NaHCO_3$ solution (150 mL) and brine (150 mL). The organic layer was dried over $MgSO_4$, and the solvents were removed under reduced pressure. The crude product was purified via column-chromatography (PE/EtOAc, 1:5, 3.5 cm × 18 cm) to give cryptophycin diol **24** (40.2 mg, 52.2 µmol, 11%) as colourless solid.

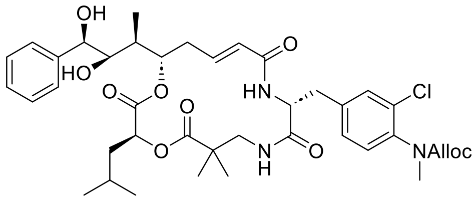

24

formula: $C_{40}H_{52}ClN_3O_{10}$

molar mass: 770.32 g/mol

yield: 40.2 mg (52.2 µmol, 11%)

TLC: R_f (PE/EtOAc, 1:5) = 0.17

HPLC-MS (ESI +): *m/z* (found) 770.3416, t_R = 9.6 min.
m/z (calc.) 770.3414 = $[M+H]^+$ = ($C_{40}H_{53}ClN_3O_{10}^+$).

^{1}H NMR (500 MHz, $CDCl_3$, *main rotamer*) δ / ppm = 7.39 – 7.27 (m, 6H, uB-C^2-**H**, uA-C^{ar}-**H**), 7.22 (m, 1H, uC-N**H**), 7.14 (m, 1H, uB-C^6**H**), 7.10 (m, 1H, uB-C^5**H**), 6.71 (m, 1H, uA-C^{β}**H**), 5.99 (m, 1H, uB-N**H**), 5.77 (m, 1H, uB-C**H**=CH_2), 5.73 (d, ^3J = 15.5 Hz, 1H, uA-C^{α}**H**), 5.12 – 5.01 (m, 3H, uA-C^{δ}**H**, uB-CH=C**H_2**), 4.88 (m, 1H, uD-C^{α}**H**), 4.75 (ddd, ^3J = 8.2 Hz, ^3J = 8.2 Hz, ^3J = 4.9 Hz, 1H, uB-C^{α}**H**), 4.66 (d, ^3J = 5.4 Hz, 1H, uB-C**H**^{A}H^{B}CH=CH_2), 4.56 (d, ^3J = 8.4 Hz, 1H, uA-C^{η}**H**), 4.50 (m, 1H, uB-CH^A**H**^{B}CH=CH_2), 3.78 (d, ^3J = 8.4 Hz, 1H, uA-C^{ζ}**H**), 3.32 (m, 1H, uC-C**H**^{A}H^B), 3.21 (m, 1H, uC-CH^A**H**^{B}), 3.18 (s, 3H, uB-NHC**H_3**), 3.10 – 2.89 (m, 2H, uB-C^{β}**H_2**), 2.46 (d (broad), ^2J = 15.2 Hz, 1H, uA-C^{γ}**H**^{A}H^B), 2.22 (dd, ^2J = 12.4 Hz, ^3J = 11.8 Hz, 1H, uA-C^{γ}H^A**H**^{B}), 1.79 (m, 1H, uD-C^{β}**H**^{A}H^B), 1.65 (m, 1H, uD-C^{γ}**H**), 1.46 (ddd, ^3J = 10.3 Hz, ^3J = 8.2 Hz, ^3J = 3.8 Hz, 1H, uD-C^{β}H^A**H**^{B}), 1.23 (s (broad), 3H, uC-C(C**H_3**)^A(CH_3)^B), 1.16 (s, 3H, uC-C(CH_3)^A(C**H_3**)^B), 1.00 (d, ^3J = 7.0 Hz, 3H, uA-C^{ε}C**H_3**), 0.94 (d, ^3J = 6.7 Hz, 3H, uD-$C^{\delta,A}$**H_3**), 0.87 (d, ^3J = 6.6 Hz, 3H, $C^{\delta,B}$**H_3**).

4.3.3 Cryptophycin-uB[D-Phe(3-Cl)(4-NMeAlloc)] 26

Cryptophycin-uA[diol]-uB[D-Phe-(3-Cl)(4-NMeAlloc)] **24** (32.2 mg, 41.8 µmol, 1.0 equiv) was converted to its ortho ester using general procedure GP I with a reaction time of 3 h. The crude product (39.5 mg) was used in the next step without further purification.

Crude ortho ester (41.8 µmol, 1.0 equiv) was converted to its bromohydrin formate using general procedure GP II with a reaction time of 4 h. The crude product (47.0 mg) was used in the next step without further purification.

Crude bromohydrin formate (41.8 µmol, 1.0 equiv) was converted to its epoxide **26** using general procedure GP IV. Purification via column chromatography (PE/EtOAc, 1:2) gave cryptophycin-uB-[D-Phe(3-Cl)(4-NMeAlloc)] **26** (21.2 mg, 28.2 µmol, 89% over 3 steps) as colourless solid.

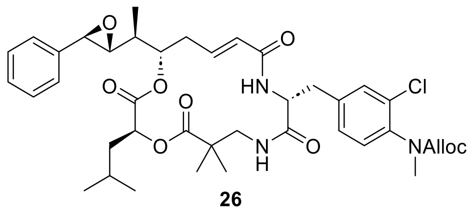

26

formula: $C_{40}H_{50}ClN_3O_9$

molar mass: 752.30 g/mol

yield: 21.2 mg, (28.2 µmol, 89% over 3 steps)

TLC: R_f (PE /EtOAc, 1:3) = 0.40

HPLC-MS (ESI +): *m/z* (found) 752.3523, t_R = 11.2 min.

m/z (calc.) 752.3308 ($C_{40}H_{51}ClN_3O_9^+$)

^{1}H NMR (600 MHz, $CDCl_3$, *main rotamer*) *δ* / ppm = 7.40 – 7.27 (m, 3H, uA-C^{ar}**H**), 7.28 – 7.22 (m, 4H, uA-C^{ar}**H**, uC-N**H**, uB-$C^{ar,2}$**H**), 7.16 (m, 1H, uB-$C^{ar,6}$**H**), 7.10 (m, 1H, uB-$C^{ar,5}$**H**), 6.75 (m, 1H, uA-$C^{β}$**H**), 5.82 – 5.69 (m, 3H, uA-$C^{α}$**H**, uB-N**H**, uB-CH_2C**H**=CH_2), 5.19 (m, 1H, uA-$C^{δ}$**H**), 5.10 – 5.02 (m, 2H, uB-CH=C**H**$_2$) 4.85 (m, 1H, uD-$C^{α}$**H**), 4.78 (ddd, ^3J = 8.2 Hz, ^3J = 8.1 Hz, ^3J = 4.8 Hz, 1H, uB-$C^{α}$**H**), 4.66 (d, ^3J 4.8 Hz, 1H, uB-C**H**^{A}H^{B}CH=CH_2), 4.54 (m, 1H, uB-CH^A**H**^{B}CH=CH_2), 3.69 (s (broad), 1H, uA-$C^{η}$**H**), 3.40 (m, 1H, uC-$C^{β}$**H**^{A}H^B), 3.20 (m, 1H, uC-$C^{β}$H^A**H**^{B}), 3.19 (s, 3H, uB-NC**H**$_3$), 2.93 (dd, ^3J = 7.5 Hz, ^3J = 2.1 Hz, 1H, uA-$C^{ζ}$**H**), 2.58 (dddd, ^3J = 14.7 Hz, ^3J = 4.4 Hz, ^3J = 2.0 Hz, ^3J = 2.0 Hz, 1H, uA-$C^{γ}$**H**^{A}H^B), 2.45 (m, 1H, uA-$C^{γ}$H^A**H**^{B}), 1.80 (m, 3H, uA-$C^{ε}$**H**), 1.75 – 1.64 (m, 2H, uD-$C^{β}$**H**^{A}H^B, uD-$C^{γ}$**H**), 1.32 (m, 1H, uD-$C^{β}$H^A**H**^{B}), 1.22 (s (broad), 3H, uC-C(C**H**$_3$)^A(CH_3)^B), 1.15 (s, 3H, uC-C(CH_3)^A(C**H**$_3$)^B), 1.14 (d, ^3J = 5.0 Hz, 3H, uA-$C^{ε}$C**H**$_3$), 0.85 (d, ^3J = 6.6 Hz, 3H, uD-$C^{δ,A}$**H**$_3$), 0.83 (d, ^3J = 6.6 Hz, 3H, $C^{δ,B}$**H**$_3$).

^{13}C NMR (151 MHz, $CDCl_3$, *main rotamer*): *δ* / ppm = 178.0 (uC-**C**(=O)), 170.6 (uD-**C**(=O)), 170.2 (uB-**C**(=O)), 165.2 (uA-**C**(=O)), 155.4 (uB-alloc, **C**(=O)), 142.1 (uA-**C**^{β}), 139.2 (uB-**C**$^{ar,4'}$), 138.1 (uB-**C**$^{ar,1'}$), 136.9 (uA-**C**^{ar,ipso}), 133.0 (uB-**C**$^{ar,3'}$), 132.8 (uB-alloc, **C**H=CH_2), 130.9 (uB-**C**$^{ar,2'}$), 130.9 (uA-**C**^{ar}), 129.7 (uB-**C**$^{ar,5'}$), 128.9 (uA-**C**^{ar}), 128.7 (uB-**C**$^{ar,6'}$), 125.7 (uA-**C**^{ar}), 124.7 (uA-**C**^{α}), 117.0 (uB-Alloc, CH=**C**H$_2$), 76.1 (uA-**C**^{δ}), 71.3 (uD-**C**^{α}), 66.4 (uB-Alloc, O**C**H$_2$CH=CH_2), 63.2 (uA-**C**^{ζ}), 59.2 (uA-**C**^{η}), 54.4 (uB-**C**^{α}), 46.7 (uC-**C**^{β}), 42.9 (uC-**C**^{α}), 40.8 (uA-**C**^{ε}), 39.5 (uD-**C**^{β}), 37.1 (uB-C^{ar}N**C**H$_3$), 37.1 (uA-**C**^{γ}), 35.9 (uB-**C**^{β}), 24.7 (uD-**C**^{γ}), 23.0 (uC-$C^{α}$(**C**H$_3$)^A(CH_3)^B), 23.0 (uD-$C^{γ}$(**C**H$_3$)^A(CH_3)^B), 22.9 (uC-$C^{α}$(CH_3)^A(**C**H$_3$)^B), 21.4 (uD-$C^{γ}$(CH_3)^A(**C**H$_3$)^B), 13.7 (uA-$C^{ε}$**C**H$_3$).

4.3.4 Cryptophycin-uB[D-Phe(3-Cl)(4-NHMe)] 1

Alloc-protected cryptophycin **26** (16.0 mg, 21.3 µmol, 1.0 equiv) and $Pd(PPh_3)_4$ (6.1 mg, 5.3 µmol, 25 mol %) were dissolved in degassed DCM (1 mL) and morpholine (7.4 µL, 8.4 µmol, 4 equiv) was added. The reaction solution was stirred at rt for 90 min. The solution was filtered over silica and eluted with DCM/MeOH (19:1, 40 mL). The solvent was removed under reduced pressure and the product was further purified via column chromatography

(DCM/MeOH, 19:1, 1.5 cm × 20 cm) and RP-HPLC (65% eluent B, isocratic) to yield cryptophycin **1** (10.5 mg, 15.7 µmol, 75%) as voluminous colourless solid.

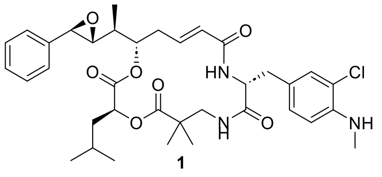

formula:	$C_{36}H_{46}ClN_3O_7$
molar mass:	668.23 g/mol
yield:	10.5 mg, (15.7 µmol, 74%)

**HPLC-MS** (ESI +):	*m/z* (found)	668.3099, t_R = 11.3 min.	
	m/z (calc.)	668.3097	$=[M+H]^+ = (C_{36}H_{47}ClN_3O_7^+)$

^{1}H NMR (600 MHz, $CDCl_3$) δ / ppm = 7.45 – 7.30 (m, 4H, 3 x uA-C^{ar}-**H**, uC-N**H**), 7.24 (dd, ^3J = 7.7 Hz, ^4J = 1.6 Hz, 2H, uA-^{Car,meta}**H**), 7.06 (d, ^4J = 2.0 Hz, 1H, uB-$C^{ar,2'}$**H**), 6.96 (dd, ^3J = 8.3 Hz, ^4J = 2.0 Hz, 1H, uB-$C^{ar,6}$**H**), 6.79 (ddd, ^3J = 15.0 Hz, ^3J = 10.9 Hz, ^3J = 4.0 Hz, 1H, uA-C^{β}**H**), 6.58 (d, ^3J = 8.2 Hz, 1H, uB-$C^{ar,5}$**H**), 5.71 (dd, ^3J = 15.1 Hz, ^4J = 1.9 Hz, 1H, uA-C^{α}**H**), 5.59 (m, 1H, uB-N**H**), 5.21 (ddd, ^3J = 11.4 Hz, ^3J = 5.2 Hz, ^3J = 2.0 Hz, 1H, uA-C^{δ}**H**), 4.82 (dd, ^3J = 10.3 Hz, ^3J = 3.5 Hz, 1H, uD-C^{α}**H**), 4.71 (m, 1H, uB-C^{α}**H**), 3.68 (d, ^3J = 2.0 Hz, 1H, uA-C^{η}**H**), 3.45 (dd, ^3J = 13.6 Hz, ^3J = 8.9 Hz, 1H, uC-C^{β}**H**^{A}H^B), 3.08 (dd, ^3J = 13.6 Hz, ^3J = 3.4 Hz, 1H, uC-C^{β}H^A**H**^{B}), 3.05 – 3.00 (m, 2H, uB-C^{β}**H**$_2$), 2.92 (dd, ^3J = 7.6 Hz, ^3J = 2.0 Hz, 1H, uA-C^{ζ}**H**), 2.89 (s, 3H, uB-C^{ar}NHC**H**$_3$), 2.59 (dddd, ^2J = 14.6 Hz, ^3J = 4.2 Hz, ^3J = 2.1 Hz, ^4J = 2.1 Hz, 1H, uA-C^{γ}**H**^{A}H^B), 2.45 (ddd, ^2J = 14.6 Hz, ^3J = 11.2 Hz, ^3J = 11.2 Hz, 1H, uA-C^{γ}H^A**H**^{B}), 1.79 (dq, ^3J = 7.1 Hz, ^3J = 7.1 Hz, ^3J = 7.1 Hz, ^3J = 5.0 Hz, ^3J = 2.1 Hz, 1H, uA-C^{ε}**H**), 1.71 (ddd, ^2J = 13.7 Hz, ^3J = 10.2 Hz, ^3J = 4.8 Hz, 1H, uD-C^{β}**H**^{A}H^B), 1.66 (m, 1H, uD-C^{γ}**H**), 1.31 (ddd, ^2J = 13.8 Hz, ^3J = 8.9 Hz, ^3J = 3.5 Hz, 1H, uD-C^{β}H^A**H**^{B}), 1.22 (s, 3H, (uC-C^{α}(C**H**$_3$)^A(CH$_3$)^B), 1.17 (s, 3H, (uC-C^{α}(CH$_3$)^A(C**H**$_3$)^B), 1.14 (d, ^3J = 6.8 Hz, 1H, uA-C^{ε}C**H**$_3$), 0.84 (d, ^3J = 6.6 Hz, 3H, uD-C^{γ}(C**H**$_3$)^A(CH$_3$)^B), 0.82 (d, ^3J = 6.5 Hz, 3H, uD-C^{γ}(CH$_3$)^A(C**H**$_3$)^B).

^{13}C NMR (151 MHz, $CDCl_3$) δ / ppm = 178.3 (uC-**C**(=O)), 171.5 (uB-**C**(=O)), 170.6 (uD-**C**(=O)), 165.3 (uA-**C**(=O)), 144.1 (uB-**C**$^{ar,4'}$), 142.6 (uA-**C**$^{\beta}$), 136.9 (uA-**C**$^{ar,1'}$), 129.7 (uB-**C**$^{ar,2'}$), 128.9 (uA-**C**^{ar}), 128.7 (uB-**C**$^{ar,6'}$), 128.7(uA-**C**^{ar}), 125.7 (uA-**C**^{ar}), 124.5 (uB-**C**$^{ar,1'}$), 124.5 (uA-**C**$^{\alpha}$), 119.6 (uB-**C**$^{ar,3'}$-Cl), 111.4 (uB-**C**$^{ar,4'}$), 76.0 (uA-**C**$^{\delta}$), 71.4 (uD-**C**$^{\alpha}$), 63.2 (uA-**C**$^{\zeta}$), 59.3 (uA-**C**$^{\eta}$), 54.6 (uB-**C**$^{\alpha}$), 46.7 (uC-**C**$^{\beta}$), 42.8 (uC-**C**$^{\alpha}$), 40.8 (uA-**C**$^{\varepsilon}$), 39.5 (uD-**C**$^{\beta}$), 37.1 (uA-**C**$^{\gamma}$), 35.3 (uB-**C**$^{\beta}$), 30.7 (uB-C^{ar}NH**C**H$_3$), 24.7 (uD-**C**$^{\gamma}$), 23.0 (uC-C$^{\alpha}$(**C**H$_3$)^A(CH$_3$)^B), 23.0 (uD-C^{γ}(**C**H$_3$)^A(CH$_3$)^B), 22.9 (uC-C^{α}(CH$_3$)^A(**C**H$_3$)^B), 21.3 (uD-C^{γ}(CH$_3$)^A(**C**H$_3$)^B), 13.8 (uA-C^{ε}**C**H$_3$).

4.4 Synthesis of cryptophycin-uB[D-Phe(4-NMe$_2$)] 2

4.4.1 uB[D-Phe(4-NMe$_2$)]-modified *seco*-cryptophycin 23

Under inert conditions Fmoc-uC-uD-uA-H **21** (1.35 g, 1.90 mmol, 1.0 equiv) was dissolved in dry DMF (12 mL). Piperidine (0.95 mL, 9.6 mmol, 5.0 equiv) was added dropwise and the reaction was stirred at rt for 1.5 h. The volatile components were removed under HV and used in the for the next step without further purification.

Under inert conditions a part of crude H-uC-uD-uA intermediate (617 mg, corresponding to 1.1 mmol, 1.1 equiv) and crude unit B **13** (338 mg, mixture which contains 0.96 mmol of **13**, 1.0 equiv) were dissolved in dry DCM (10 mL). NEt_3 (0.42 mL, 3.0 mmol, 3.2 equiv) and HOAt (207 mg, 1.52 mmol, 1.6 equiv) were added at 0 °C and stirred for 10 min. EDC·HCl (294 mg, 1.52 mmol, 1.6 equiv) was added portionwise over 20 min at 0 °C. The reaction mixture was stirred for 18 h while warming up to rt. H_2O (100 mL) and EtOAc (150 mL) were added, and the phases were separated. The aqueous layer was extracted with EtOAc (3 × 100 mL). The combined organic layers were dried over $MgSO_4$, and the solvent was evaporated. Purification via column chromatography (PE/EtOAc, 2:1 to 1:2) and column chromatography (DCM/MeOH, 95:5) gave uB-D-Phe(4-NMe$_2$)-modified *seco*-cryptophycin **23** (178 mg, 0.21 mmol, 22%) and an impure product fraction, which was further purified using automated RP-column chromatography (Büchi-Ecoflex C18, 12 g, eluent A: H_2O/ACN, 95:5; eluent B: H_2O/ACN, 95:5; gradient: 20% B to 100% B in 19 column volumes) to give *seco*-cryptophycin **23** (92 mg, 0.13 mmol, 13%) as a colourless solid (total yield: 270 mg, 368 µmol, 38%).

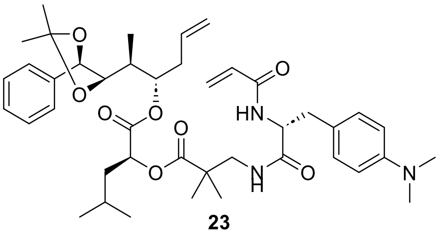

23

formula:	$C_{42}H_{59}N_3O_8$
molar mass:	733.95 g/mol
yield:	270 mg (368 µmol, 38%)

TLC: R$_f$ (PE/EtOAc, 1:1) = 0.19
R$_f$ (DCM/MeOH, 95:5) = 0.40

^{1}H NMR (600 MHz, $CDCl_3$,) δ / ppm = 7.39 – 7.34 (m, 4H, uA-C^{ar}**H**), 7.31 (m, 1H, uA-C^{ar}**H**), 7.02 (d, ^3J = 8.3 Hz, 2H, uB-C$^{ar,2'}$**H**, uB-C$^{ar,6'}$**H**), 6.95 (dd, ^3J = 8.0 Hz, ^3J = 4.8 Hz, 1H, uC-N**H**), 6.64 (d, ^3J = 8.2 Hz, 2H, uB-C$^{ar,3'}$**H**, uB-C$^{ar,5'}$**H**), 6.30 (m, 1H, uB-N**H**), 6.24 (dd, ^3J = 17.0 Hz, ^2J = 1.4 Hz, 1H, uB-CH=C**H**^{A}H^B), 6.06 (dd, ^3J = 17.0, ^3J = 10.3 Hz, 1H, uB-C**H**=CH^{A}H^B), 5.58 (dd, ^3J = 10.3 Hz, ^2J = 1.4 Hz, 1H, uB-CH=CH^A**H**^{B}), 5.52 (dddd, ^3J = 17.2 Hz, ^3J = 10.2 Hz, ^3J = 7.1 Hz, ^3J = 7.1 Hz, 1H, uA-C$^{\beta}$**H**), 4.98 – 4.92 (m, 3H, uA-C$^{\alpha}$**H**^{A}H^B, uA-C$^{\delta}$**H**, uD-C$^{\alpha}$**H**), 4.87 (dd, ^3J = 17.1 Hz, ^2J = 1.7 Hz, 1H, uA-C$^{\alpha}$H^A**H**^{B}), 4.72 (ddd, ^3J = 7.6 Hz, ^3J = 6.4 Hz, ^3J = 6.4 Hz, 1H, uB-C$^{\alpha}$**H**), 4.70 (d, ^3J = 8.8 Hz, 1H, uA-C$^{\eta}$**H**), 3.85 (dd, ^3J = 8.8 Hz, ^3J = 2.3 Hz, 1H, uA-C$^{\zeta}$**H**),

3.54 (dd, ^2J = 13.4 Hz, ^3J = 8.1 Hz, 1H, uC-C$^{\beta}$**H**^{A}H^B), 3.24 (dd, ^2J = 13.4 Hz, ^3J = 4.8 Hz, 1H, uC-C$^{\beta}$H^A**H**^{B}), 3.05 (dd, ^2J = 14.0 Hz, ^3J = 6.4 Hz, 1H, uB-C$^{\beta}$**H**^{A}H^B), 3.00 (dd, ^2J = 14.1 Hz, ^3J = 6.2 Hz, 1H, uB-C$^{\beta}$H^A**H**^{B}), 2.89 (s, 6H, uB-C^{ar}N(C**H**$_3$)$_2$), 2.32 – 2.15 (m, 2H, uA-C$^{\gamma}$**H**^{A}**H**^{B}), 1.92 (qdd, ^3J = 7.0 Hz, ^3J = 7.0 Hz, ^3J = 2.2 Hz, 1H, uA-C$^{\varepsilon}$**H**), 1.73 – 1.63 (m, 2H, uD-C$^{\beta}$**H**^{A}H^B, uD-C$^{\gamma}$**H**), 1.55 (m, 1H, uD-C$^{\beta}$H^A**H**^{B}), 1.51 (s, 3H, uA-C(C**H**$_3$)^A(CH$_3$)^B), 1.46 (s, 3H, uA-C(CH$_3$)^A(C**H**$_3$)^B), 1.15 (s, 3H, uC-C$^{\alpha}$(C**H**$_3$)^A(CH$_3$)^B), 1.13 (s, 3H, uC-C$^{\alpha}$(C**H**$_3$)^A(CH$_3$)^B), 1.10 (d, ^3J = 7.0 Hz, 3H, uA-C$^{\varepsilon}$C**H**$_3$), 0.92 (d, ^3J = 6.4 Hz, 3H, uD-C$^{\gamma}$(C**H**$_3$)^A(CH$_3$)^B), 0.86 (d, ^3J = 6.4 Hz, 3H, uD-C$^{\gamma}$(CH$_3$)^A(C**H**$_3$)^B).

^{13}C NMR (151 MHz, CDCl$_3$) δ / ppm = 175.8 (uC-**C**O$_2$), 171.4 (uB-C$^{\alpha}$**C**(=O)), 171.3 (uD-**C**O$_2$), 165.0 (uB-**C**(=O)CH=CH$_2$), 149.6, (uB-**C**$^{ar,4'}$), 137.8 (uA-C^{ar,ipso}), 132.6 (uA-**C**$^{\beta}$), 130.9 (uB-**C**H=CH$_2$), 130.1 ((uB-**C**$^{ar,2'}$, uB-**C**$^{ar,6'}$), 128.9 (uA-**C**^{ar}), 128.6 (uA-**C**^{ar,p}), 126.9 (uA-**C**^{ar}), 126.6 (uB-CH=**C**H$_2$), 124.4 (uB-**C**$^{ar,1'}$), 118.6 (uA-**C**$^{\alpha}$), 112.9 (uB-**C**$^{ar,3'}$, uB-**C**$^{ar,5'}$), 109.0 (uA-**C**(CH$_3$)^A(CH$_3$)^B), 82.0 (uA-**C**$^{\zeta}$), 80.5 (uA-**C**$^{\eta}$), 76.8 (uD-**C**$^{\alpha}$), 70.9 (uA-**C**$^{\delta}$), 54.7 (uB-**C**$^{\alpha}$), 47.4 (uC-**C**$^{\beta}$), 43.8 (uC-**C**$^{\alpha}$), 40.8 (uB-C^{ar}N(**C**H$_3$)$_2$), 39.5 (uD-**C**$^{\beta}$), 37.8 (uB- **C**$^{\beta}$), 35.7 (uA-**C**$^{\gamma}$), 35.3 (uA-**C**$^{\varepsilon}$), 27.4 (uA-C(**C**H$_3$)^A(CH$_3$)^B), 27.2 (uA-C(CH$_3$)^A(**C**H$_3$)^B), 24.9 (uD-**C**$^{\beta}$), 23.4 (uC-C$^{\alpha}$(CH$_3$)^A(**C**H$_3$)^B), 23.3 (uD-C$^{\gamma}$(**C**H$_3$)^A(CH$_3$)^B), 22.4 (uC-C$^{\alpha}$(CH$_3$)^A(**C**H$_3$)^B), 21.4 (uD-C$^{\gamma}$(CH$_3$)^A(**C**H$_3$)^B, 10.0 (uA-C$^{\varepsilon}$**C**H$_3$).

4.4.2 Modified cryptophycin-uA[diol]-uB[D-Phe(4-NMe$_2$)] 25

Under inert conditions *seco*-cryptophycin **23** (270 mg, 368 µmol, 1.0 equiv) was dissolved in dry DCM (20 mL) and Grubbs-II catalyst (17 mg, 20 µmol, 5 mol %) was added. The reaction was refluxed for 3 h. The solvent was removed under reduced pressure. Purification of the residue by column chromatography (PE/EtOAc, 1:2) gave uB[D-Phe(4-NMe$_2$)]-modified cryptophycin acetonide (218 mg, 309 µmol, 84%) as colourless solid.

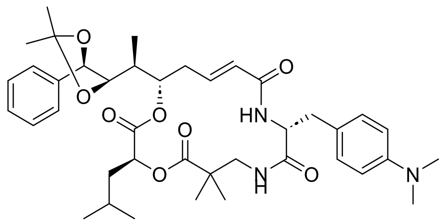

formula:	$C_{40}H_{55}N_3O_8$
molar mass:	705.89 g/mol
yield:	218 mg (309 µmol, 84%)

TLC: R$_f$ (PE/EtOAc, 1:2) = 0.26

HPLC-MS (ESI +): *m/z* (found) 706.4214, t_R = 11.4 min.

m/z (calc.) 706. 4062 = [M+H]$^+$ = ($C_{40}H_{56}N_3O_8^+$).

^{1}H NMR (500 MHz, CDCl$_3$) δ / ppm = 7.39–7.28 (m, 5H, uA-C^{Ar}**H**), 7.20 (dd, ^3J = 9.3 Hz, ^3J = 3.0 Hz, 1H, uC-N**H**), 7.02 (d, ^3J = 8.3 Hz, 2H, uB-C$^{ar2'}$**H,** uB-C$^{ar6'}$**H**), 6.67–6.56 (m, 3H, uA-C$^{\beta}$**H**, uB-C$^{ar3'}$**H**, uB-C$^{ar5'}$**H**), 5.63 (dd, ^3J = 15.2 Hz, ^3J = 1.9 Hz, 1H, uA-C$^{\alpha}$**H**), 5.56 (m, 1H, uB-N**H**), 5.05 (ddd, ^3J = 11.5 Hz, ^3J = 7.0 Hz, ^3J = 2.1 Hz, 1H, uA-C$^{\delta}$**H**), 4.78 (dd, ^3J = 10.3 Hz, ^3J = 3.8

Hz, 1H, uD-C$^{\alpha}$**H**), 4.69 (d, ^{3}J = 8.8 Hz, 1H, uA-C$^{\eta}$**H**), 4.65 (dd, ^{3}J = 7.2 Hz, ^{3}J = 6.3 Hz, 1H, uB-C$^{\alpha}$**H**), 3.78 (dd, ^{3}J = 8.8 Hz, ^{3}J = 2.3 Hz, 1H, uA-C$^{\zeta}$**H**), 3.46 (dd, ^{2}J = 13.5 Hz, ^{3}J = 9.2 Hz, 1H, uC-C$^{\beta}$**H**^{A}H^{B}), 3.06 – 2.98 (m, 3H, uB-C$^{\beta}$**H**^{A}H^{B}, uB-C$^{\beta}$H^{A}**H**^{B}, uC-C$^{\beta}$H^{A}**H**^{B}), 2.91 (s, 6H, uB-N(C**H**$_3$)$_2$), 2.44 (dddd, ^{2}J = 14.8 Hz, ^{3}J = 4.4 Hz, ^{3}J = 2.3 Hz, ^{4}J = 2.3 Hz, 1H, uA-C$^{\gamma}$**H**$_A$), 2.22 (ddd, ^{2}J = 14.6, ^{3}J = 11.2 Hz, ^{3}J = 11.2 Hz, 1H, uA-C$^{\gamma}$**H**$_B$), 1.82 (qdd, ^{3}J = 6.9 Hz, ^{3}J = 6.9 Hz, ^{3}J = 2.3 Hz, 1H, uA-C$^{\varepsilon}$**H**), 1.74 (ddd, ^{2}J = 13.9, ^{3}J = 10.3 Hz, ^{3}J = 5.2 Hz, 1H, uD-C$^{\beta}$**H**$_A$), 1.65 (m, 1H, uD-C$^{\gamma}$**H**), 1.49 (s, 3H, uA-C(C**H**$_3$)^{A}(CH$_3$)^{B}), 1.45 (s, 3H, uA-C(C**H**$_3$)^{A}(C**H**$_3$)^{B}), 1.34 (ddd, ^{2}J = 14.0, ^{3}J = 8.7 Hz, ^{3}J = 3.8 Hz, 1H, uD-C$^{\beta}$H^{A}**H**^{B}), 1.21 (s, 3H, uC-C$^{\alpha}$(C**H**$_3$)^{A}(CH$_3$)^{B}), 1.15 (s, 3H, uC-C$^{\alpha}$(CH$_3$)^{A}(C**H**$_3$)^{B}), 1.11 (d, ^{3}J = 6.9 Hz, 3H, uA-C$^{\varepsilon}$C**H**$_3$), 0.91 (d, ^{3}J = 6.7 Hz, 3H, uD-C$^{\gamma}$(C**H**$_3$)^{A}(CH$_3$)^{B}), 0.81 (d, ^{3}J = 6.6 Hz, 3H, uD-C$^{\gamma}$(CH$_3$)^{A}(C**H**$_3$)^{B}).

^{13}C NMR (126 MHz, CDCl$_3$) δ / ppm = 178.2 (uC-**C**(=O)O), 171.0 (uB-**C**(=O)NH), 170.2 (uD-**C**(=O)O), 165.1 (uA-**C**(=O)NH), 149.8 (uB-**C**$^{ar,4'}$), 142.2 (uA-**C**$^{\beta}$), 137.7 (uA-**C**^{ar,ipso}), 129.9 (uB-**C**$^{ar,2'}$, uB-**C**$^{ar,6'}$), 128.9 (uA-**C**^{ar}), 128.7 (uA-**C**^{ar}), 128.7 (uA-**C**^{ar}), 126.7 (2x uA-**C**^{ar}), 124.5 (uA-**C**$^{\alpha}$), 123.8 (uB-**C**$^{ar,1'}$), 109.3 (uA-**C**(CH$_3$)$_2$), 82.5 (uA-**C**$^{\zeta}$), 80.3 (uA-**C**$^{\eta}$), 75.7 (uA-**C**$^{\delta}$), 71.2 (uD-**C**$^{\alpha}$), 54.7 (uB-**C**$^{\alpha}$), 46.5 (uC-**C**$^{\beta}$), 42.9 (uC-**C**$^{\alpha}$), 40.9 (uB-N(**C**H$_3$)$_2$), 39.6 (uD-**C**$^{\beta}$), , 37.0 (uA-**C**$^{\varepsilon}$), 35.9 (uA-**C**$^{\gamma}$), 35.6 (uB-**C**$^{\beta}$), 27.3 (uA-C(**C**H$_3$)^{A}(CH$_3$)^{B}), 27.2 (uA-C(CH$_3$)^{A}(**C**H$_3$)^{B}), 24.8 (uD-**C**$^{\gamma}$), 23.0 (uD-**C**$^{\gamma}$(CH$_3$)^{A}(**C**H$_3$)^{B}), 23.0 (uC-C(**C**H$_3$)^{A}), 22.8 (uC-C(CH$_3$)^{A}(**C**H$_3$)^{B}), 21.5 (uD-**C**$^{\gamma}$(CH$_3$)^{A}(**C**H$_3$)^{B}), 9.8 (uA-C$^{\varepsilon}$**C**H$_3$).

uB-D-Phe(4-NMe$_2$)-modified cryptophycin acetonide (212 mg, 309 µmol, 1.0 equiv) was dissolved in DCM (2.2 mL). H_2O (11 drops) and TFA (2.2 mL) were added at 0 °C and the reaction was stirred for 5 h at rt. The solvent was removed under vacuum and the residue was dissolved in sat. $NaHCO_3$ (100 mL) and EtOAc (75 mL). The phases were separated, and the aqueous layer was extracted with EtOAc (3 × 50 mL). The combined organic layers were dried over $MgSO_4$, and the solvent was evaporated. Drying in HV over night gave uB[D-Phe(4-NMe$_2$)]-modified cryptophycin diol **25** (187 mg, 281 µmol, 91%) as colourless solid, which was used in the next step without further purification.

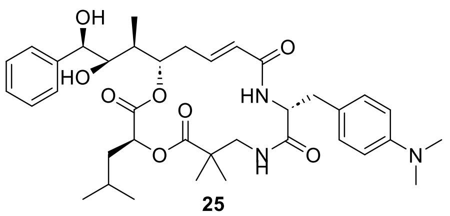

25

formula:	$C_{37}H_{51}N_3O_8$
molar mass:	665.8 g/mol
yield:	187 mg (281 µmol, 91%)

**HPLC-MS** (ESI +):	*m/z* (found)	666.3934, t_R = 7.3 min.	
	m/z (calc.)	666.3749	= [M+H]$^+$ = ($C_{37}H_{52}N_3O_8^+$).

4.4.3 Cryptophycin-uB[D-Phe(4-NMe2)] 2

Cryptophycin-uA[diol]-uB[D-Phe(4-NMe2)] **25** (187 mg, 280 µmol, 1.0 equiv) was converted to its ortho ester using general procedure GP I with a reaction time of 3 h. For the silica column (EtOAc/DCM, 1:1, 400 mL) was used. The crude product (134 mg, 68%) was used in the next step without further purification.

Crude ortho ester was converted (in two batches) to its bromohydrin formate using general procedure GP II. The first batch (30 mg, 42.3 µmol, 1.0 equiv) gave the bromohydrin formate (22 mg, 29.0 mmol, 69%). The second batch (99 mg, 139 µmol, 1.0 equiv) was converted with AcBr solution (0.5 M, 0.66 mL, 2.5 equiv) for 3 h and AcBr solution (1.0 M, 0.35 mL, 2.5 equiv) for 3.7 h and gave the bromohydrin formate (88.1 mg, 116 µmol, 83%). Both batches were combined and used in the next step without further purification.

Crude bromohydrin formate (145 µmol, 1.0 equiv) was converted to its epoxide **2** using general procedure GP IV. Purification via column chromatography (PE/EtOAc, 1:3) gave cryptophycin-uB[D-Phe(4-NMe2)] **2** (26 mg, 40 µmol, 14% over 3 steps) as colourless solid.

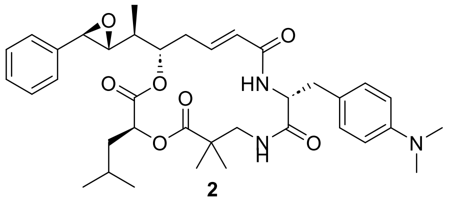

2

formula:	$C_{37}H_{49}N_3O_7$
molar mass:	647.8 g/mol
yield:	26 mg (40 µmol, 14% over 3 steps)

TLC: R_f (PE /EtOAc, 1:3) = 0.22

HPLC-MS (ESI +): *m/z* (found) 648.3714, t_R = 9.0 min.
m/z (calc.) 648.3643 = $[M+H]^+$ = ($C_{37}H_{50}N_3O_7^+$).

^{1}H NMR (600 MHz, $CDCl_3$) δ / ppm = 7.39 – 7.31 (m, 3H, uA-C^{ar}**H**), 7.24 (dd, ^3J = 7.8 Hz, ^4J = 1.2 Hz, 2H, uA-C^{ar}**H**), 7.21 (dd, ^3J = 9.0 Hz, ^3J = 2.0 Hz, 1H, uC-N**H**), 7.02 (d, ^3J = 8.0 Hz, 2H, uB-C$^{ar,2'}$**H**, uB-C$^{ar,6'}$**H**), 6.77 (ddd, ^3J = 14.9 Hz, ^3J = 10.9 Hz, ^3J = 3.9 Hz, 1H, uA-C$^{\beta}$**H**), 6.65 (s, 2H, uB-C$^{ar,3'}$**H**, uB-C$^{ar,5'}$**H**), 5.68 (dd, ^3J = 15.0 Hz, ^4J = 1.9 Hz, 1H, uA-C$^{\alpha}$**H**), 5.49 (s (broad), 1H, uB-N**H**), 5.22 (ddd, ^3J = 11.5 Hz, ^3J = 5.2 Hz, ^3J = 2.0 Hz, 1H, uA-C$^{\delta}$**H**), 4.83 (dd, ^3J = 10.3 Hz, ^3J = 3.5 Hz, 1H, uD-C$^{\alpha}$**H**), 4.69 (ddd, ^3J = 6.4 Hz, ^3J = 6.4 Hz, ^3J = 6.4 Hz, 1H, uB-C$^{\alpha}$**H**), 3.67 (d, ^3J = 1.9 Hz, 1H, uA-C$^{\eta}$**H**), 3.49 (dd, ^2J = 13.5 Hz, ^3J = 9.3 Hz, 1H, uC-C$^{\beta}$**H**^{A}H^B), 3.08 (dd, ^2J = 14.4 Hz, ^3J = 6.8 Hz, 1H, uB-C$^{\beta}$**H**^{A}H^B), 3.05 – 2.98 (m, 2H, uB-C$^{\beta}$H^A**H**^{B}, uC-C$^{\beta}$H^A**H**^{B}), 2.92 (s, 6H, uB-C^{ar}N(C**H**$_3$)$_2$), 2.91 (m, 1H, uA-C$^{\zeta}$**H**), 2.57 (dddd, ^2J = 14.7 Hz, ^3J = 4.1 Hz, ^3J = 2.1 Hz, ^4J = 2.1 Hz, 1H, uA-C$^{\gamma}$**H**^{A}H^B), 2.44 (ddd, ^2J = 14.6 Hz, ^3J = 11.2 Hz, ^3J = 11.2 Hz, 1H, uA-C$^{\gamma}$H^A**H**^{B}), 1.77 (ddd, ^3J = 7.2 Hz, ^3J = 7.2 Hz, ^3J = 5.2 Hz, 1H, uA-C$^{\varepsilon}$**H**), 1.72 (m, 1H, uD-C$^{\beta}$**H**^{A}H^B), 1.65 (m, 1H, uD-C$^{\gamma}$**H**), 1.28 (m, 1H, uD-C$^{\beta}$H^A**H**^{B}), 1.22 (s, 3H, uC-

$C^{\alpha}(C\mathbf{H}_3)^A(CH_3)^B$), 1.16 (s, 3H, uC-$C^{\alpha}(CH_3)^A(C\mathbf{H}_3)^B$), 1.14 (d, ^3J = 6.9 Hz, 3H, uA-$C^{\varepsilon}C\mathbf{H}_3$), 0.84 (d, ^3J = 6.6 Hz, 3H, uD-$C^{\gamma}(C\mathbf{H}_3)^A(CH_3)^B$), 0.81 (d, ^3J = 6.5 Hz, 3H uD-$C^{\gamma}(CH_3)^A(C\mathbf{H}_3)^B$).

^{13}C NMR (151 MHz, $CDCl_3$) δ / ppm = 178.4 (uC-**C**(=O)), 170.9 170.2 (uB-**C**(=O)), 170.6 (uD-**C**(=O)), 164.8 (uA-**C**(=O)), 149.9 (uB-$\mathbf{C}^{ar,4'}$), 141.8 (uA-$\mathbf{C}^{\beta}$), 136.9 (uA-$\mathbf{C}^{ar,ipso}$), 130.0 (uB-$\mathbf{C}^{ar,2'}$, uB-$\mathbf{C}^{ar,6'}$), 128.9 (uA-$\mathbf{C}^{ar}$), 128.7 (uA-$\mathbf{C}^{ar}$), 125.7 (uA-$\mathbf{C}^{ar}$), 124.8 (uA-$\mathbf{C}^{\alpha}$), 123.6 (uB-$\mathbf{C}^{ar,1'}$), 113.1 (uB-$\mathbf{C}^{ar,3'}$, uB-$\mathbf{C}^{ar,5'}$), 75.9 (uA-$\mathbf{C}^{\delta}$), 71.3(uD-$\mathbf{C}^{\alpha}$), 63.2 (uA-$\mathbf{C}^{\zeta}$), 59.3 (uA-$\mathbf{C}^{\eta}$), 54.7 (uB-$\mathbf{C}^{\alpha}$), 46.4 (uC-$\mathbf{C}^{\beta}$), 42.9 (uC-$\mathbf{C}^{\alpha}$), 40.9 (uA-$\mathbf{C}^{\varepsilon}$), 40.8 (uB-C^{ar}-N($\mathbf{C}H_3)_2$), 39.5 (uD-$\mathbf{C}^{\beta}$), 37.0 (uA-$\mathbf{C}^{\gamma}$), 35.6 (uB-$\mathbf{C}^{\beta}$), 24.7 (uD-$\mathbf{C}^{\gamma}$), 23.0 (uC-$C^{\alpha}(\mathbf{C}H_3)^A(CH_3)^B$), 23.0 (uC-$C^{\alpha}(CH_3)^A(\mathbf{C}H_3)^B$), 22.9 (uD-$C^{\gamma}(\mathbf{C}H_3)^A(CH_3)^B$), 21.3 (uD-$C^{\gamma}(CH_3)^A(\mathbf{C}H_3)^B$), 13.8. (uA-$C^{\varepsilon}\mathbf{C}H_3$).

5 References

[1] S. Eissler, M. Nahrwold, B. Neumann, H.-G. Stammler, N. Sewald, *Org. Lett.* **2007**, *9*, 817.

[2] B. Sammet, T. Bogner, M. Nahrwold, C. Weiss, N. Sewald, *J. Org. Chem.* **2010**, *75*, 6953.

[3] M. Nahrwold, T. Bogner, S. Eissler, S. Verma, N. Sewald, *Org. Lett.* **2010**, *12*, 1064.

[4] T. Weide, L. Arve, H. Prinz, H. Waldmann, H. Kessler, *Bioorg. Med. Chem. Lett.* **2006**, *16*, 59.

[5] G. R. Fulmer, A. J. M. Miller, N. H. Sherden, H. E. Gottlieb, A. Nudelman, B. M. Stoltz, J. E. Bercaw, K. I. Goldberg, *Organometallics* **2010**, *29*, 2176.

[6] C. Dessin, T. Schachtsiek, J. Voss, A.-C. Abel, B. Neumann, H.-G. Stammler, A. E. Prota, N. Sewald, *Angew. Chem. Int. Ed.* **2024**, e202416210.

[7] O. V. Dolomanov, L. J. Bourhis, R. J. Gildea, J. A. K. Howard, H. Puschmann, *J Appl Cryst* **2009**, *42*, 339.

[8] G. M. Sheldrick, *Acta Cryst A* **2015**, *71*, 3.

[9] L. J. Bourhis, O. V. Dolomanov, R. J. Gildea, J. A. K. Howard, H. Puschmann, *Acta Cryst A* **2015**, *71*, 59.

[10] F. Kleemiss, O. V. Dolomanov, M. Bodensteiner, N. Peyerimhoff, L. Midgley, L. J. Bourhis, A. Genoni, L. A. Malaspina, D. Jayatilaka, J. L. Spencer, F. White, B. Grundkötter-Stock, S. Steinhauer, D. Lentz, H. Puschmann, S. Grabowsky, *Chem. Sci.* **2020**, *12*, 1675.

[11] L. Wang, W. Hisano, Y. Murai, M. Sakurai, Y. Muto, H. Ikemoto, M. Okamoto, T. Murotani, R. Isoda, D. Kim, Y. Sakihama, I. R. Sitepu, Y. Hashidoko, Y. Hatanaka, M. Hashimoto, *Molecules* **2013**, *18*, 8393.

[12] V. V. Tolstyakov, E. S. Tolstobrova, O. S. Zarubina, E. A. Popova, A. V. Protas, S. S. Chuprun, R. E. Trifonov, *Russ. J. Org. Chem.* **2016**, *52*, 1681.

[13] S. P. Arns, J. B. Jaquith, D. J. Baudelet, E. R. Simonson, R. T. Liggins, N. S. Kumar, T. H. H. Hsieh, WO2017/136943, **2017**.

[14] J. Juodaityte, N. Sewald, *J. Biotechnol.* **2004**, *112*, 127.

[15] E. Byun, B. Hong, K. A. de Castro, M. Lim, H. Rhee, *J. Org. Chem.* **2007**, *72*, 9815.

[16] G. Li, Z. Qin, A. T. Radosevich, *J. Am. Chem. Soc.* **2020**, *142*, 16205.

[17] V. F. Patel, S. L. Andis, J. H. Kennedy, J. E. Ray, R. M. Schultz, *J. Med. Chem.* **1999**, *42*, 2588.

[18] E. Figueras, A. Borbély, M. Ismail, M. Frese, N. Sewald, *Beilstein J. Org. Chem.* **2018**, *14*, 1281.

6 Appendix

6.1 NMR spectra

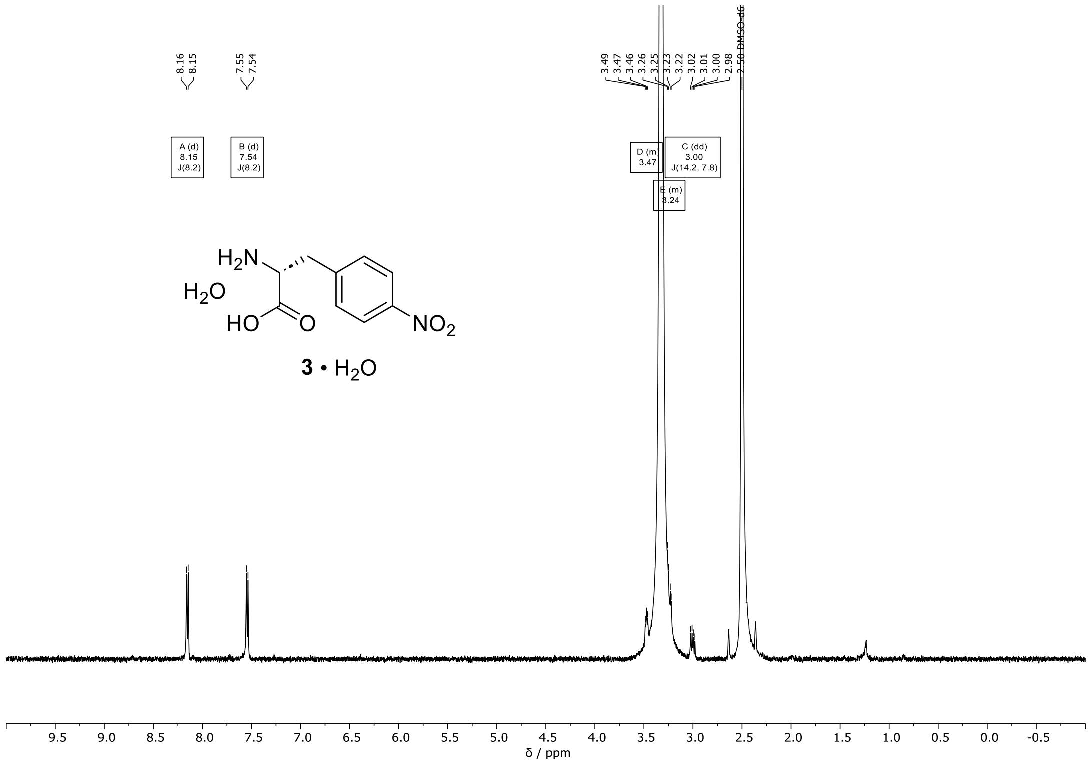

NMR spectrum 1: ^{1}H NMR spectrum (500 MHz, DMSO-d_6) of H-D-Phe(4-NO_2)-OH **3**· H_2O.

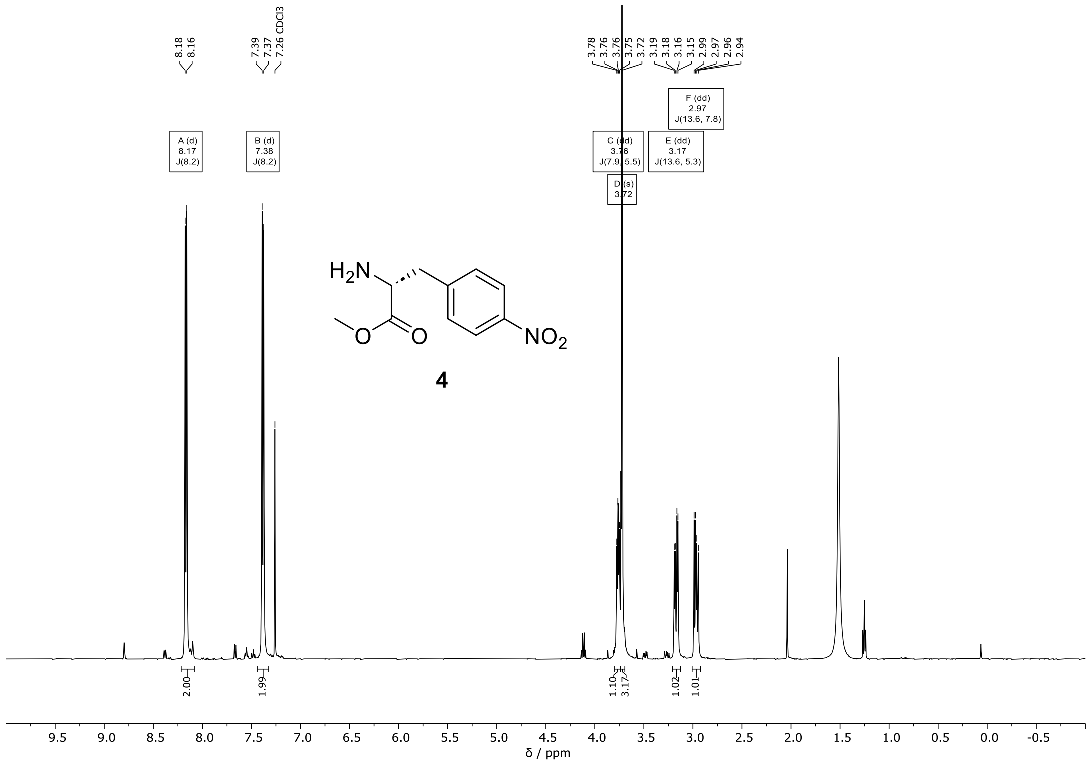

NMR spectrum 2: ^{1}H NMR spectrum (500 MHz, $CDCl_3$) of H-D-Phe(4-NO_2)-OMe (**4**).

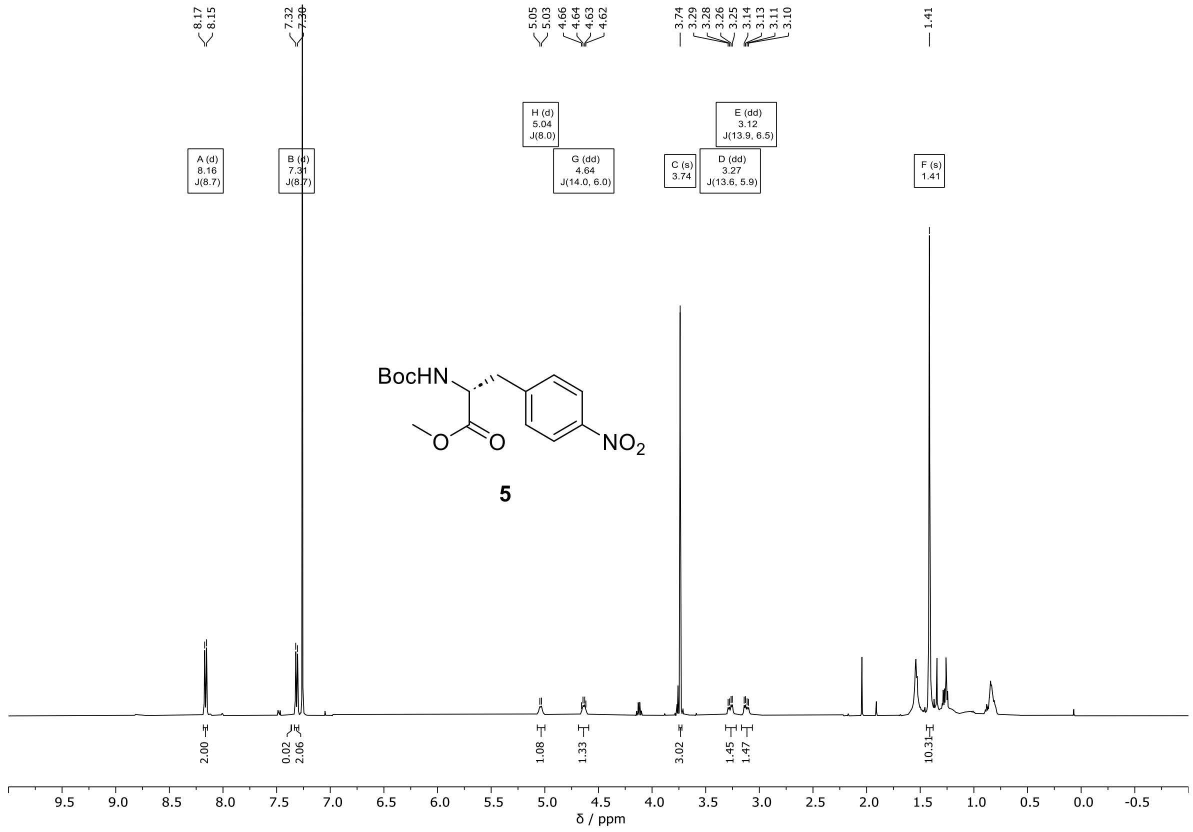

NMR spectrum 3: ^{1}H NMR spectrum (500 MHz, $CDCl_3$) of Boc-D-Phe(4-NO_2)-OMe (**5**).

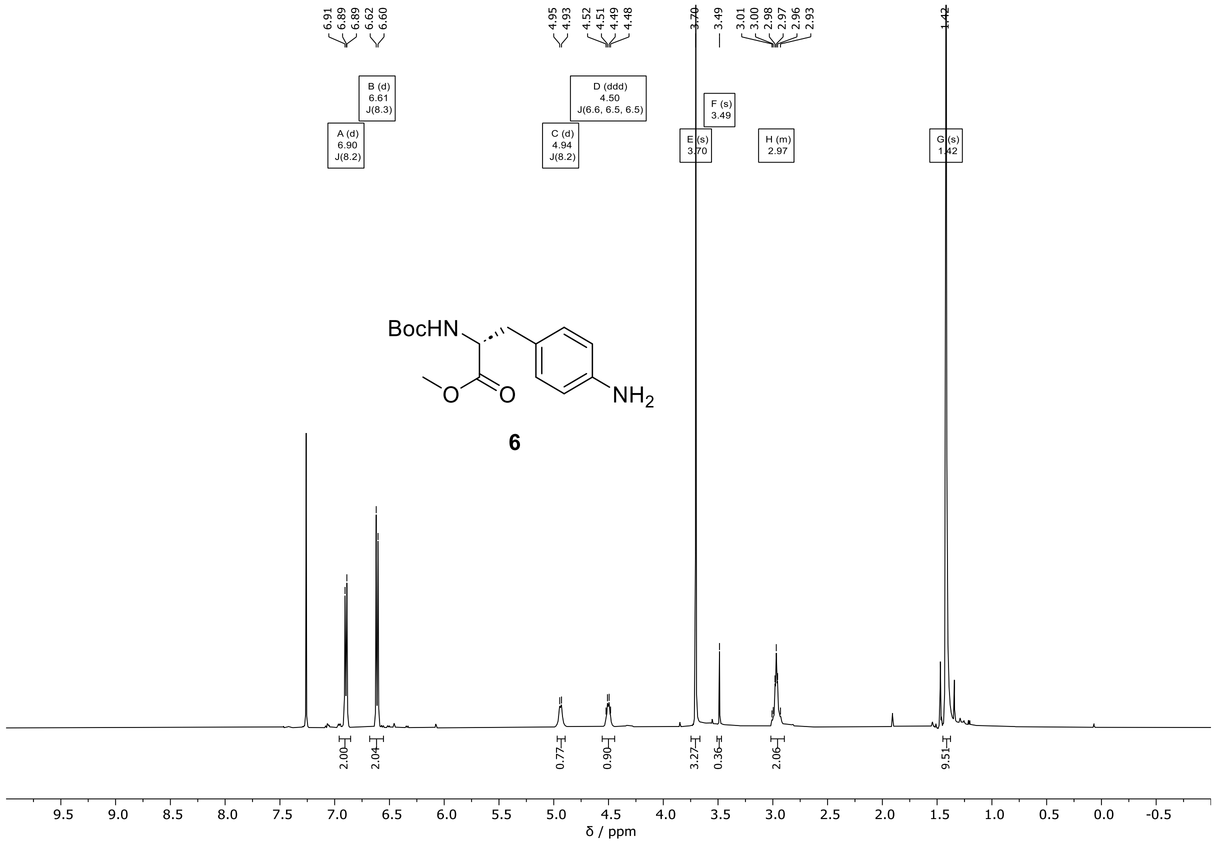

NMR spectrum 4: ^{1}H NMR spectrum (500 MHz, $CDCl_3$) of Boc-D-Phe(4-NH_2)-OMe (**6**).

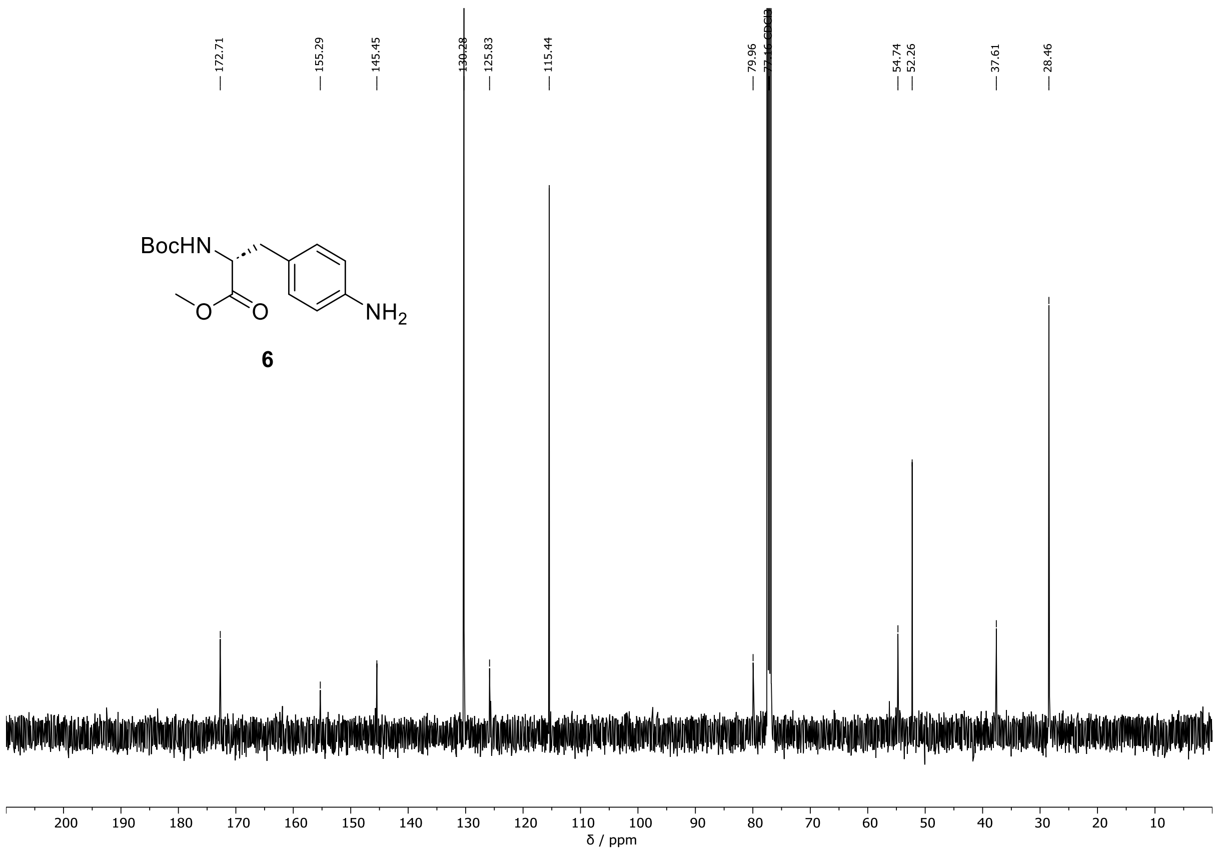

NMR spectrum 5: $^{13}C\{^1H\}$ NMR spectrum (125 MHz {500 MHz}, $CDCl_3$) of Boc-D-Phe(4-NH$_2$)-OMe (**6**).

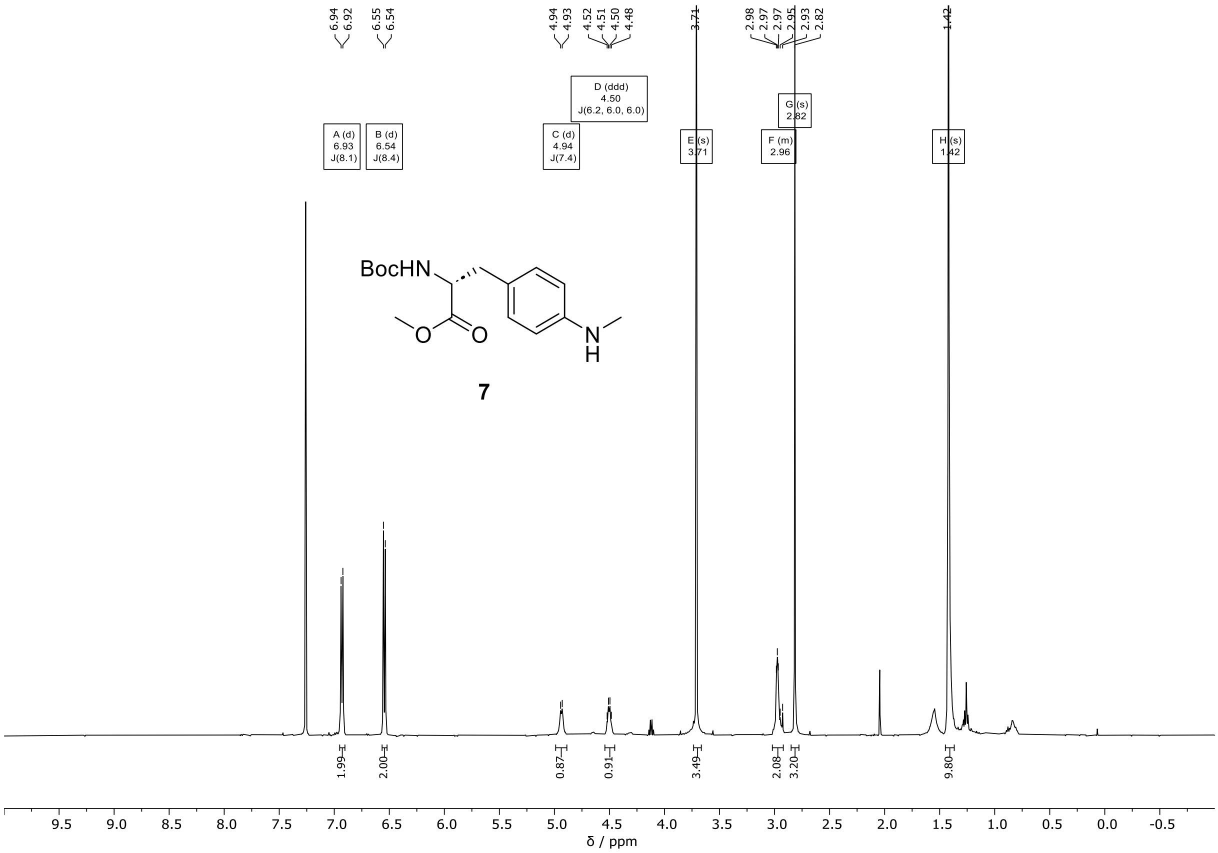

NMR spectrum 6: ^1H NMR spectrum (500 MHz, $CDCl_3$) of Boc-D-Phe(4-NHMe)-OMe (**7**).

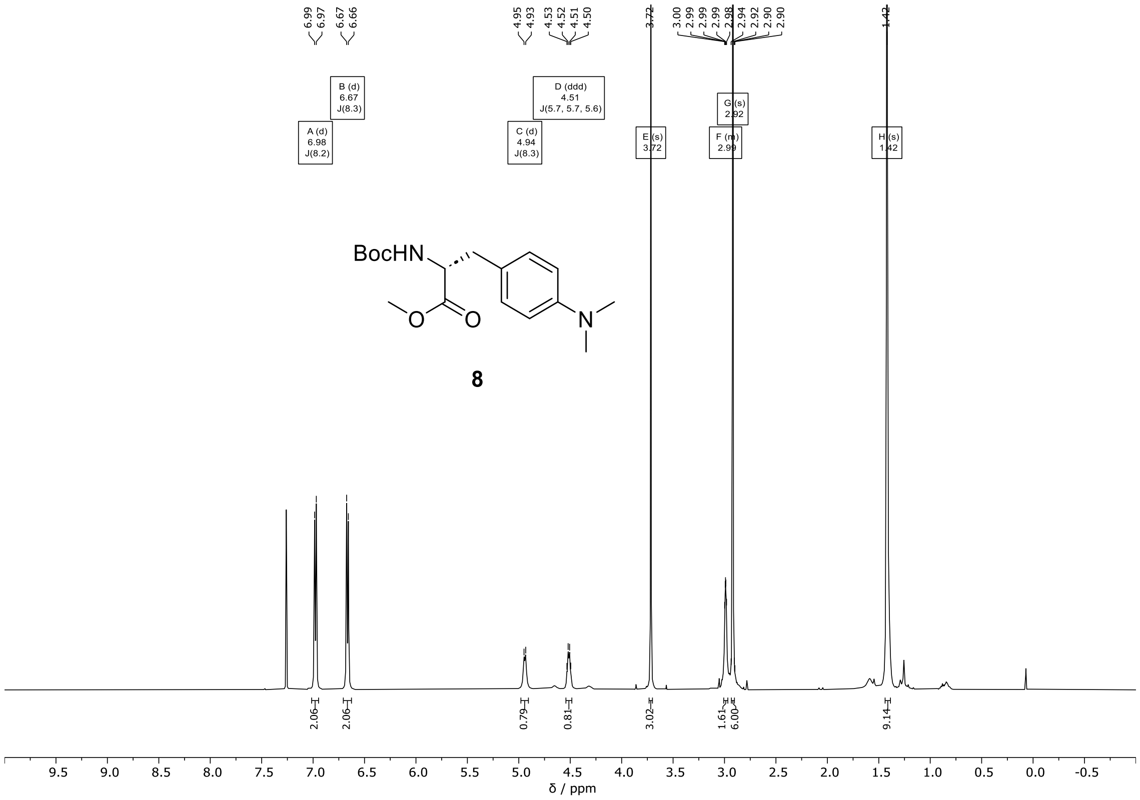

NMR spectrum 7: ^{1}H NMR spectrum (500 MHz, $CDCl_3$) of Boc-D-Phe(4-NMe_2)-OMe (**8**).

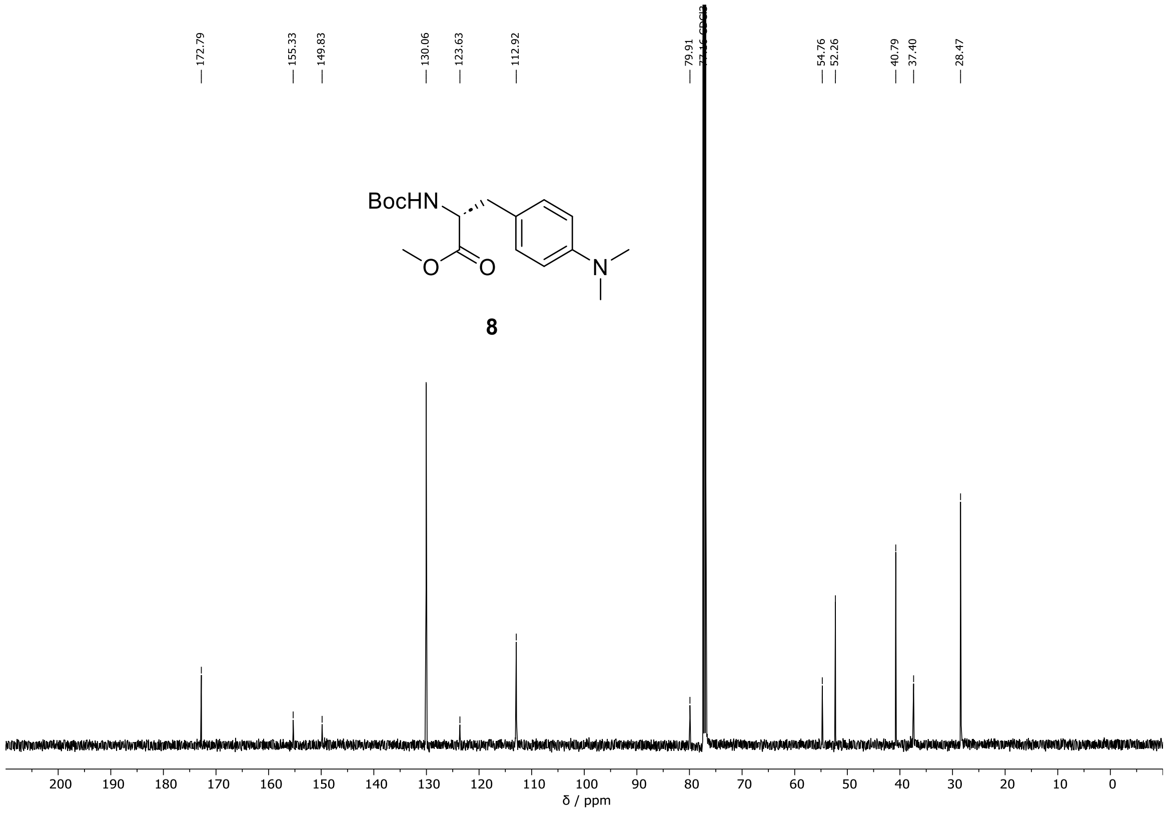

NMR spectrum 8: $^{13}C\{^{1}H\}$ NMR spectrum (125 MHz {500 MHz}, $CDCl_3$) of Boc-D-Phe(4-NMe_2)-OMe (**8**).

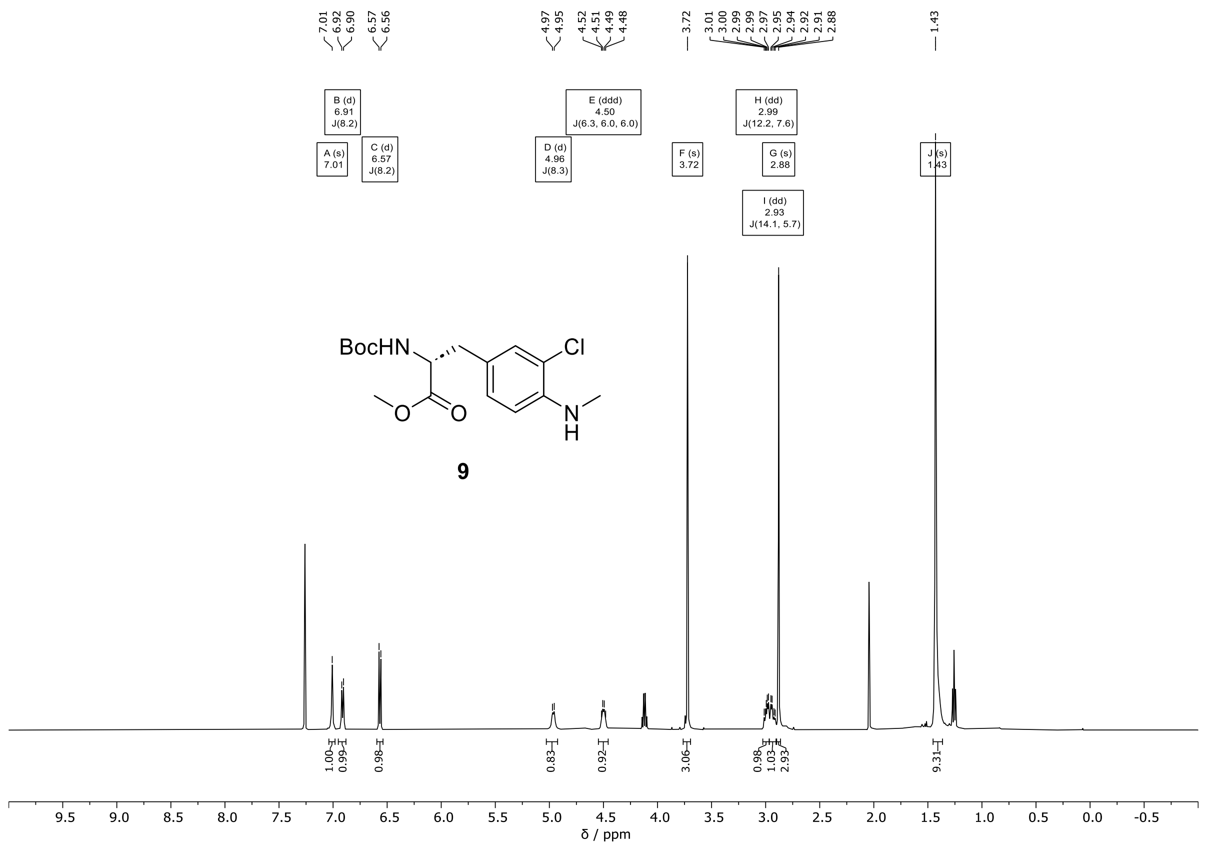

NMR spectrum 9: ^{1}H NMR spectrum (500 MHz, $CDCl_3$) of Boc-D-Phe(3-Cl)(4-NHMe)-OMe (**9**).

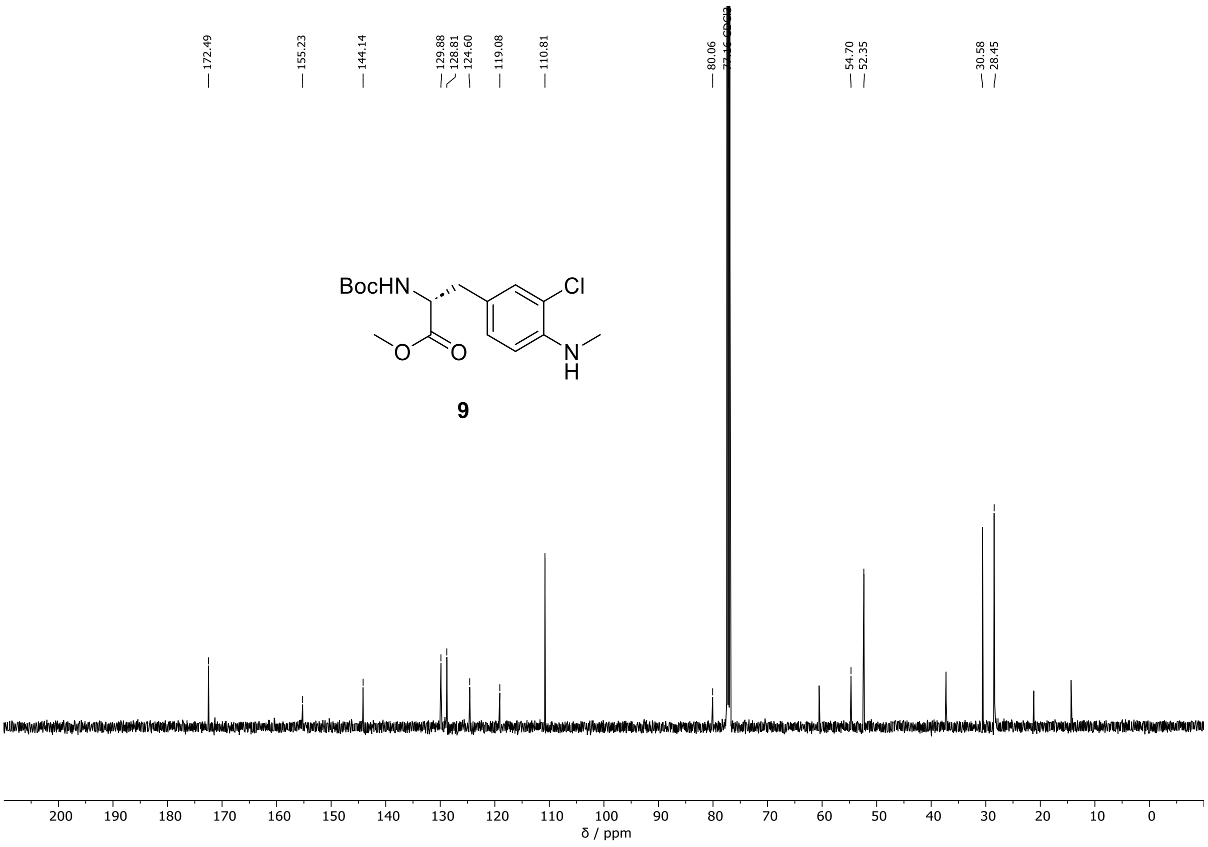

NMR spectrum 10: $^{13}C\{^{1}H\}$ NMR spectrum (125 MHz {500 MHz}, $CDCl_3$) of Boc-D-Phe(3-Cl)(4-NHMe)-OMe (**9**).

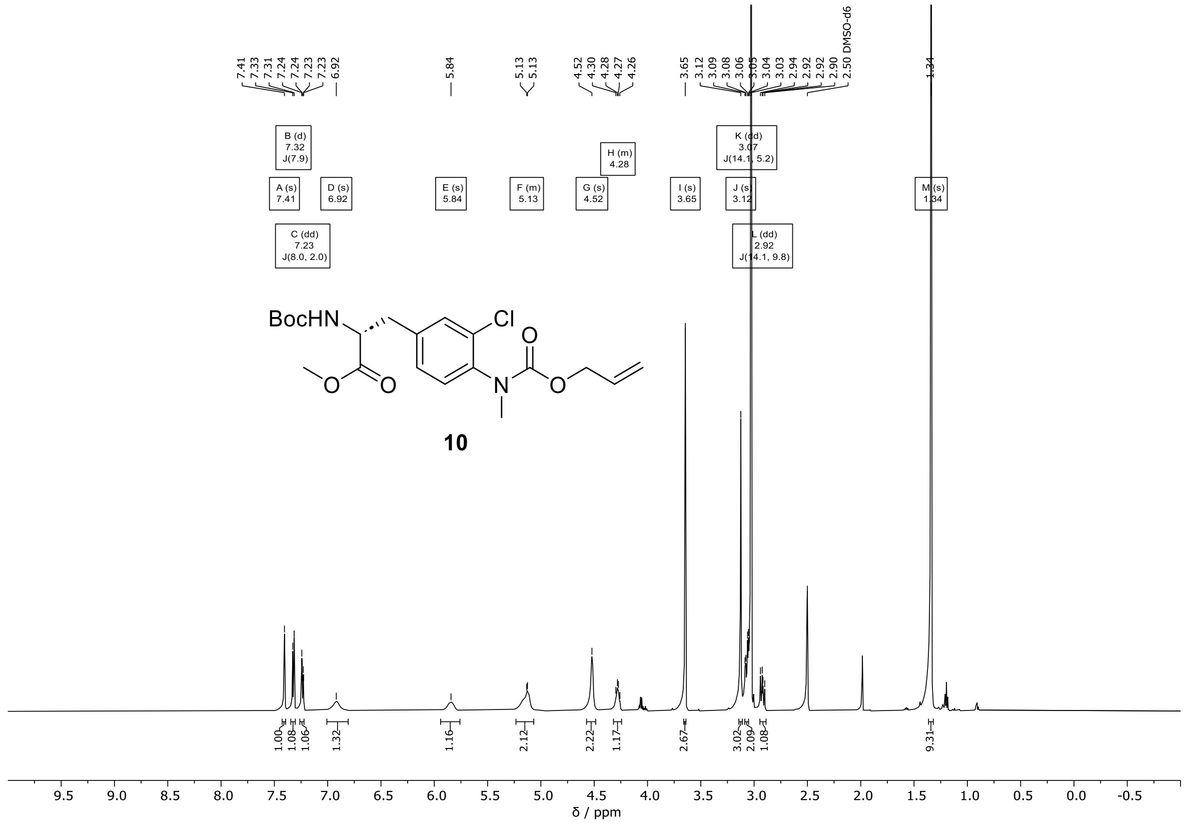

NMR spectrum 11: ^{1}H NMR spectrum (500 MHz, DMSO-d_6, 90 °C) of Boc-D-Phe(3-Cl)(4-NMeAlloc)-OMe (**10**).

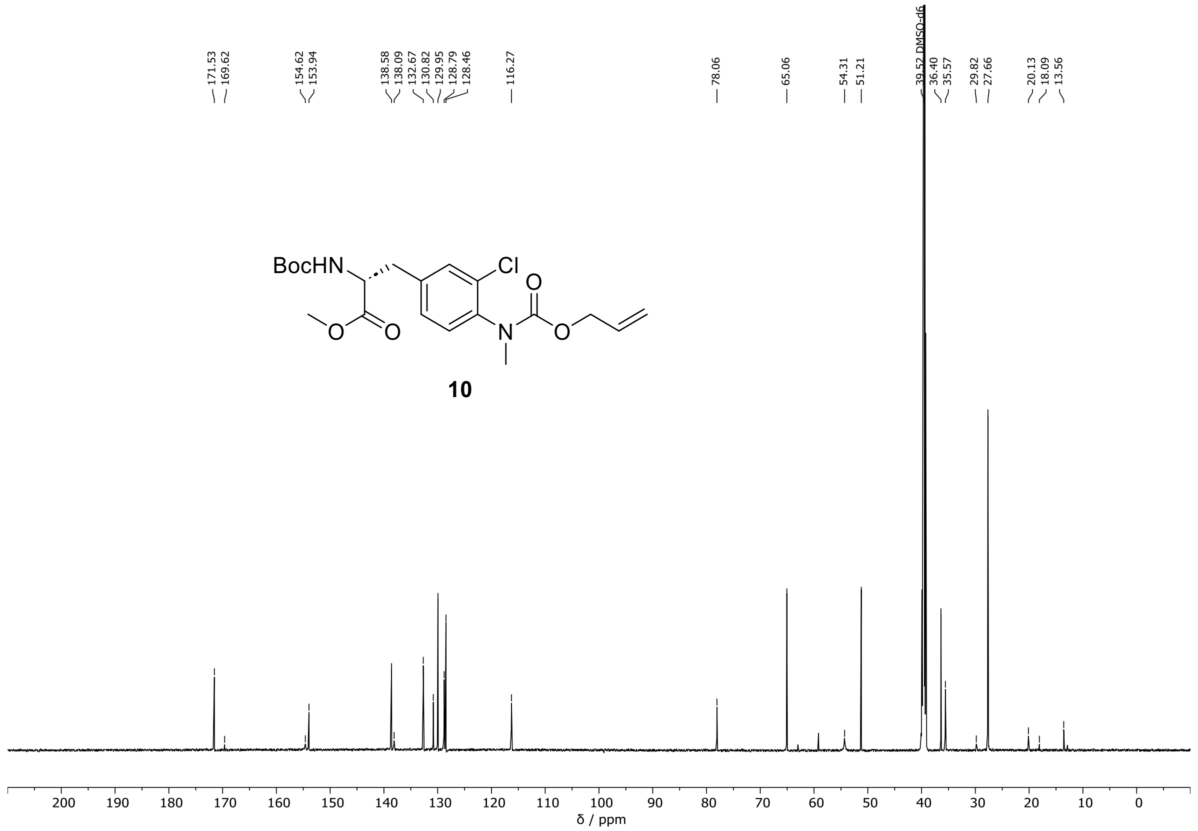

NMR spectrum 12: ^{13}C{^{1}H} NMR spectrum (125 MHz {500 MHz}, DMSO-d_6, 90 °C) of Boc-D-Phe(3-Cl)(4-NMeAlloc)-OMe (**10**).

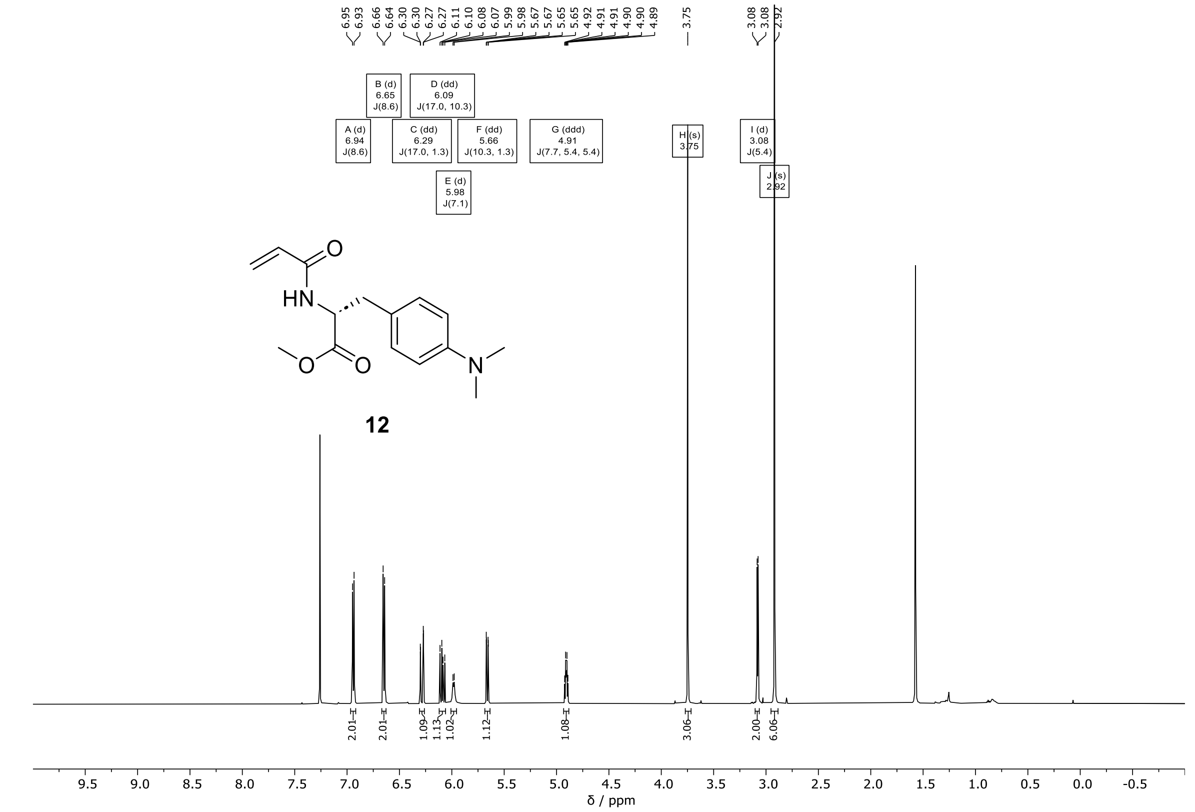

NMR spectrum 13: ^{1}H NMR spectrum (500 MHz, $CDCl_3$) of acryloyl-D-Phe(4-NMe_2)-OMe (**12**).

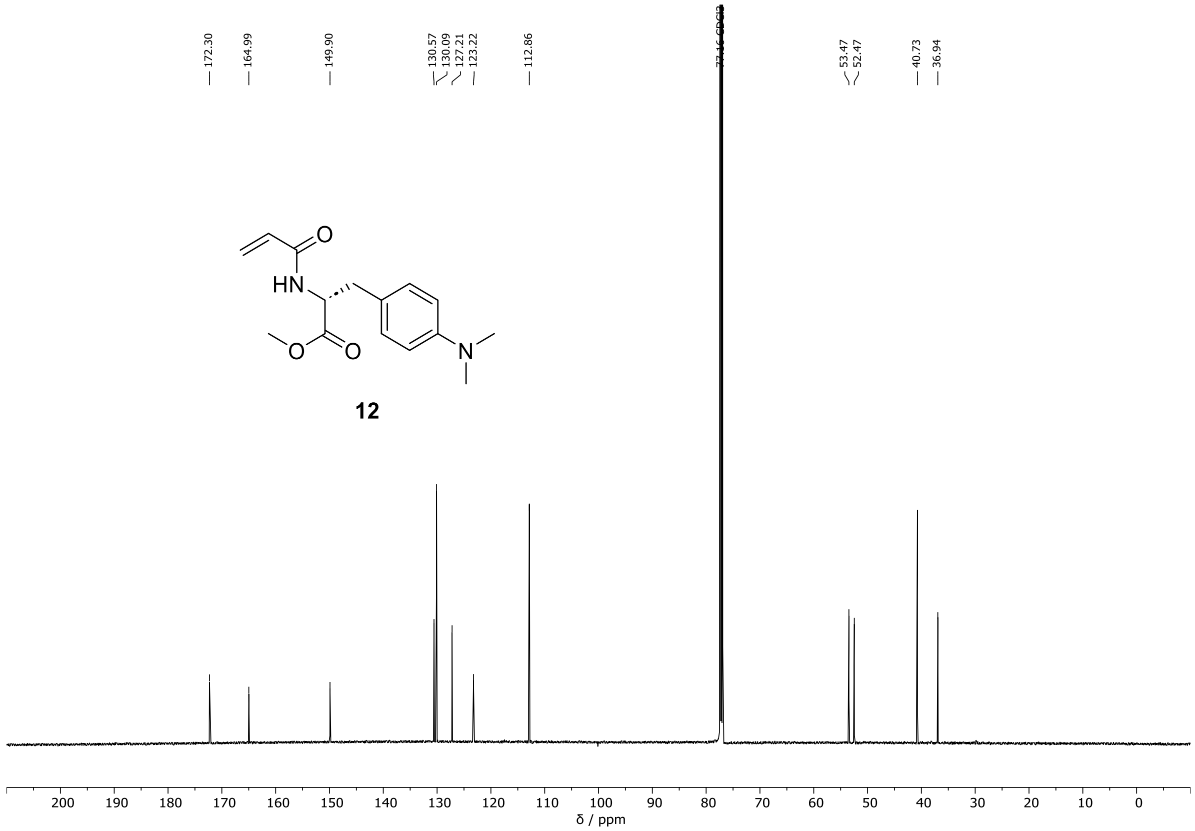

NMR spectrum 14: $^{13}C\{^{1}H\}$ NMR spectrum (125 MHz {500 MHz}, $CDCl_3$) of acryloyl-D-Phe(4-NMe_2)-OMe (**12**).

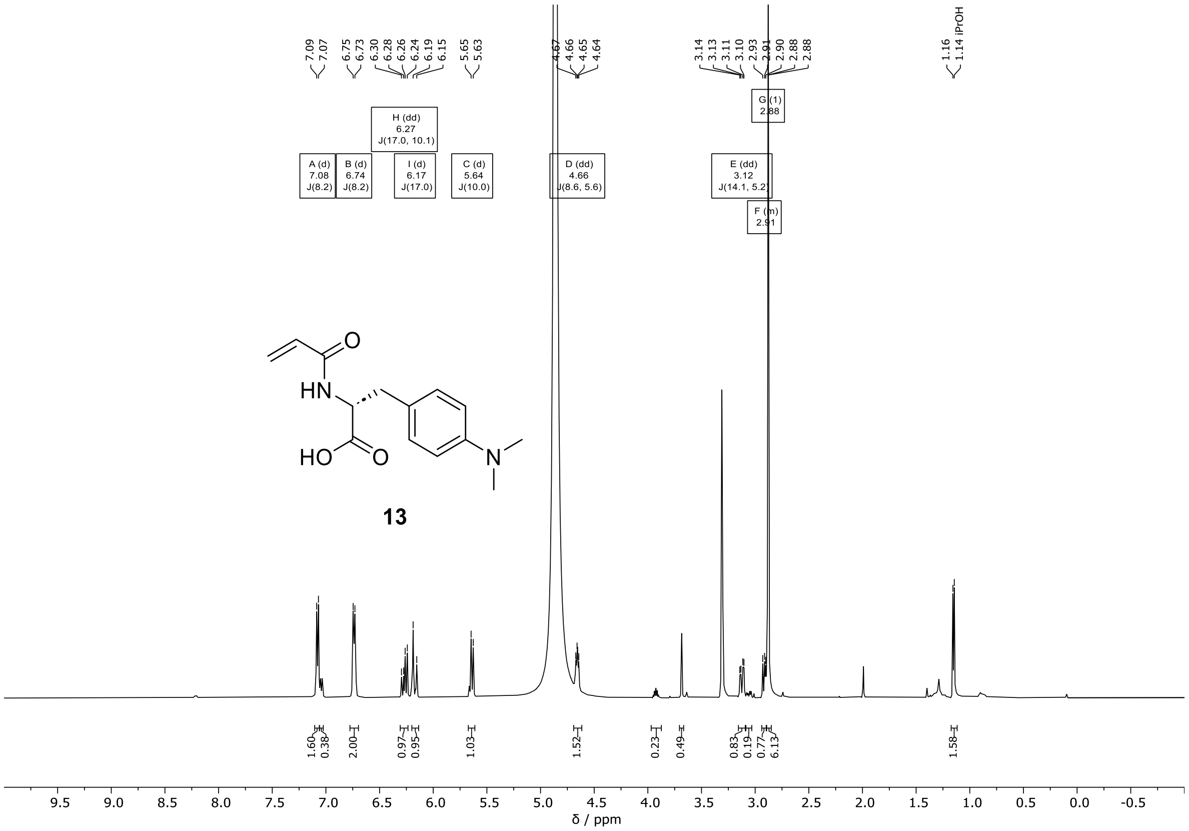

NMR spectrum 15: ^{1}H NMR spectrum (500 MHz, CD_3OD) of acryloyl-D-Phe(4-NMe_2)-OH **13** with residual acryloyl-D-Phe(4-NMe_2)-OMe (**12**).

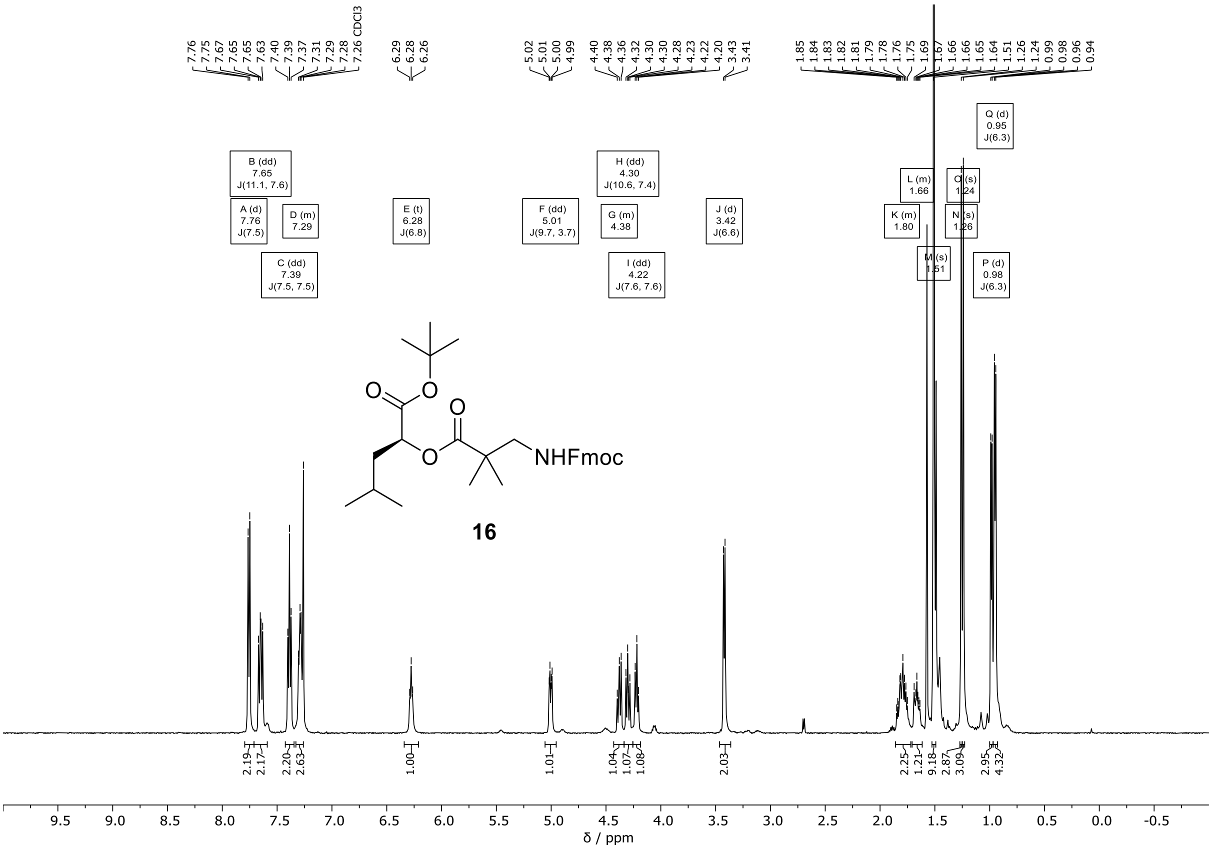

NMR spectrum 16: ^{1}H NMR spectrum (500 MHz, $CDCl_3$) of Fmoc-uC-uD-O*t*-Bu **16**.

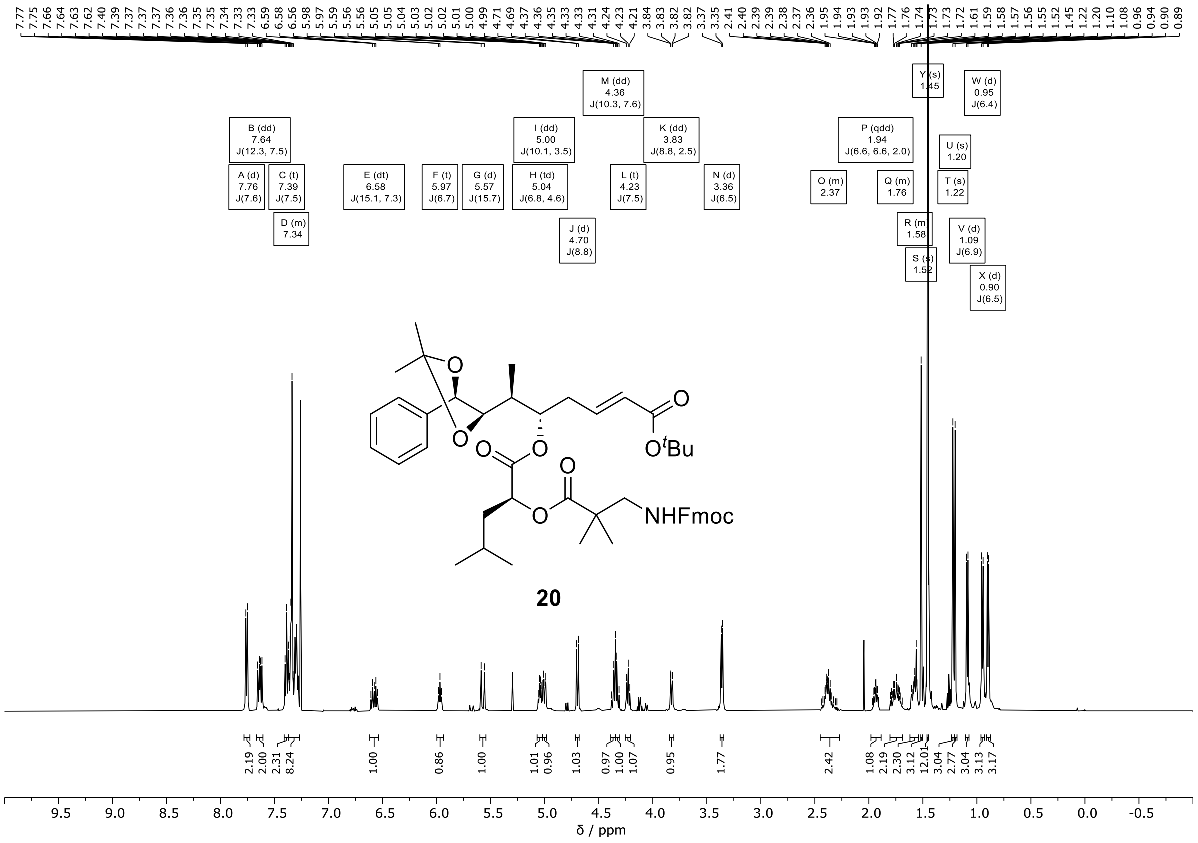

NMR spectrum 17: ^{1}H NMR spectrum (500 MHz, $CDCl_3$) of Fmoc-uC-uD-uA-CO_2t-Bu **20**.

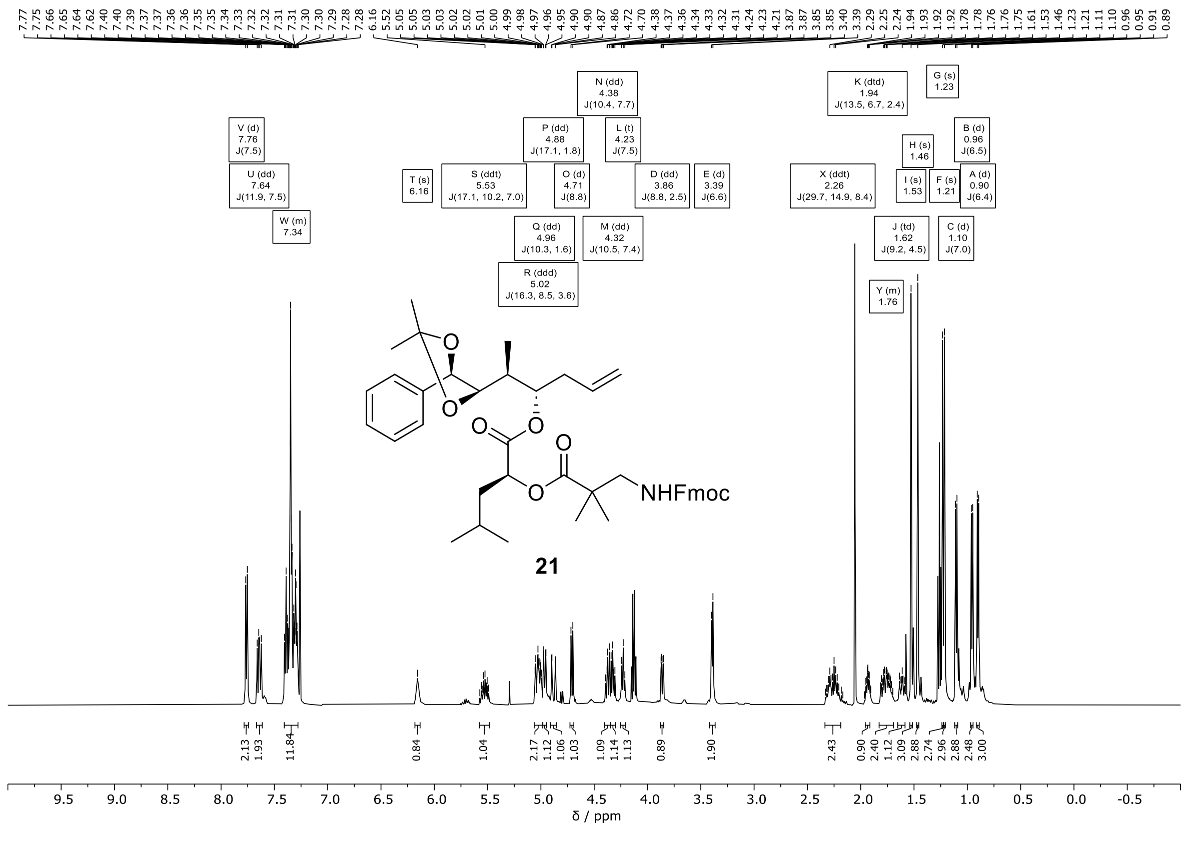

NMR spectrum 18: ^{1}H NMR spectrum (500 MHz, $CDCl_3$) of Fmoc-uC-uD-uA-H **21**.

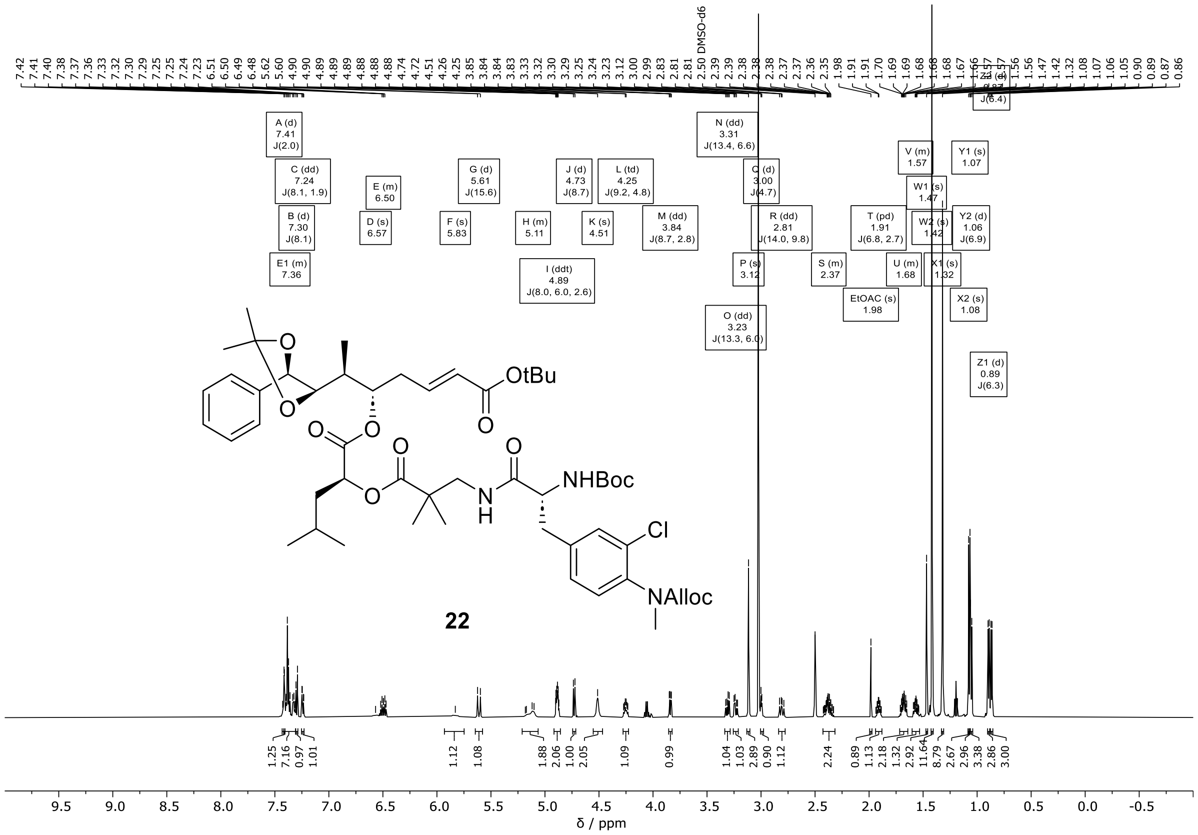

NMR spectrum 19: ^{1}H NMR spectrum (500 MHz, DMSO-d_6, 90 °C) of uB[D-Phe(3-Cl)(4-NMeAlloc)]-modified *seco*-cryptophycin **22**.

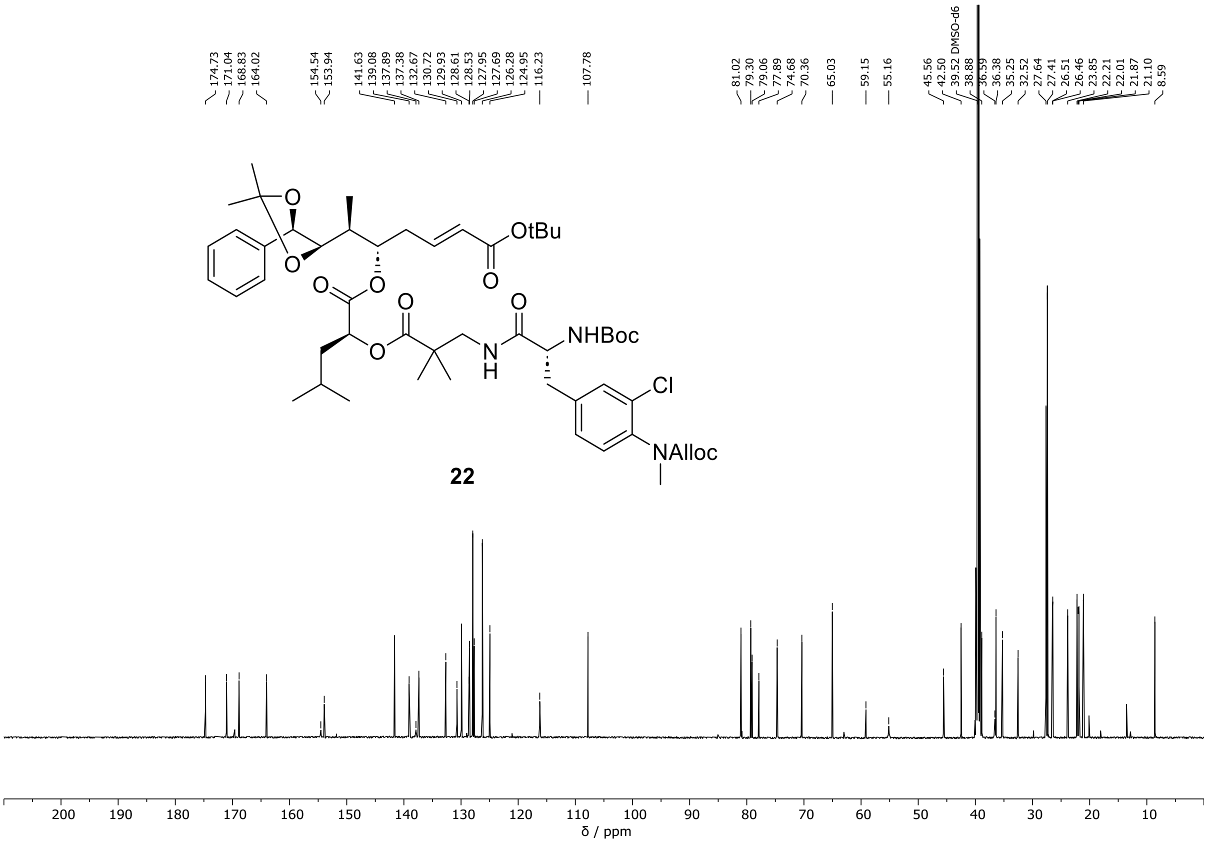

NMR spectrum 20: ^{13}C{^{1}H} NMR spectrum (125 MHz {500 MHz}, DMSO-d_6, 90 °C) of uB[D-Phe(3-Cl)(4-NMeAlloc)]-modified *seco*-cryptophycin **22**.

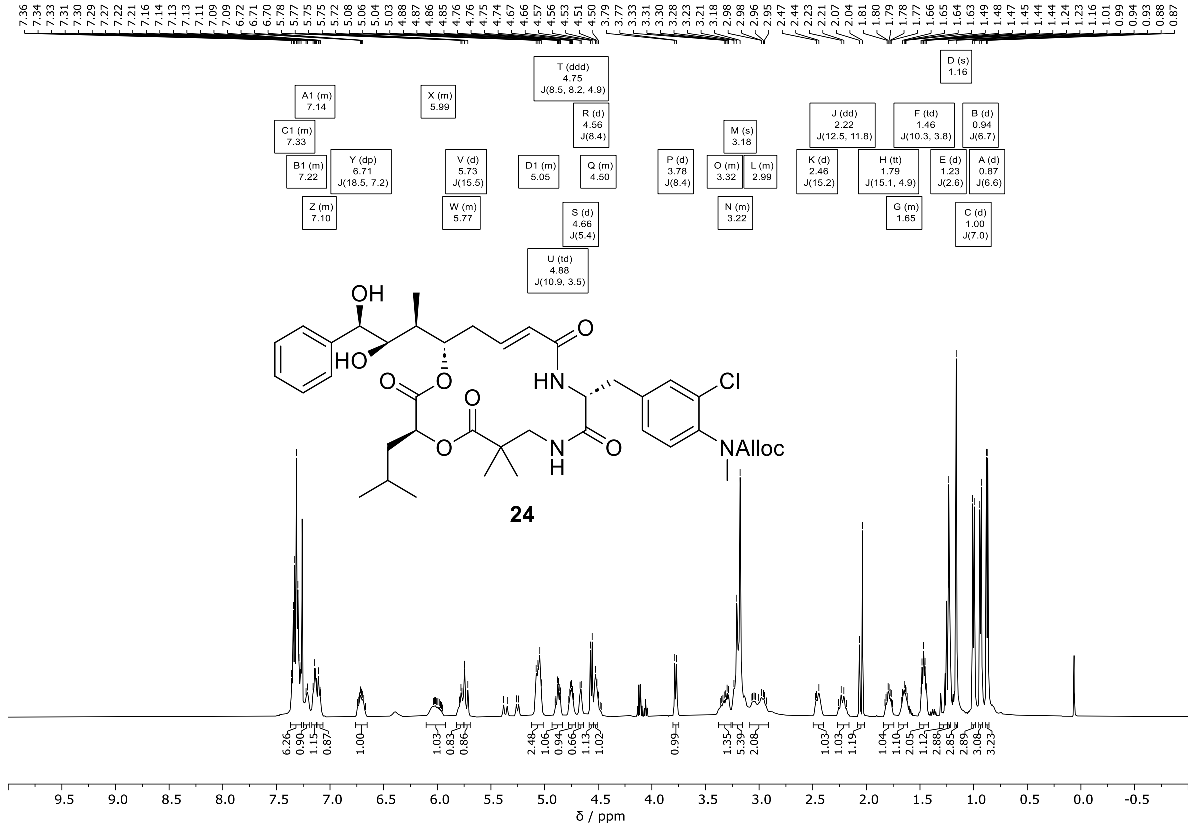

NMR spectrum 21: ^{1}H NMR spectrum (500 MHz, $CDCl_3$) of modified cryptophycin-uA[diol]-uB[D-Phe(3-Cl)(4-NMeAlloc)] **24**.

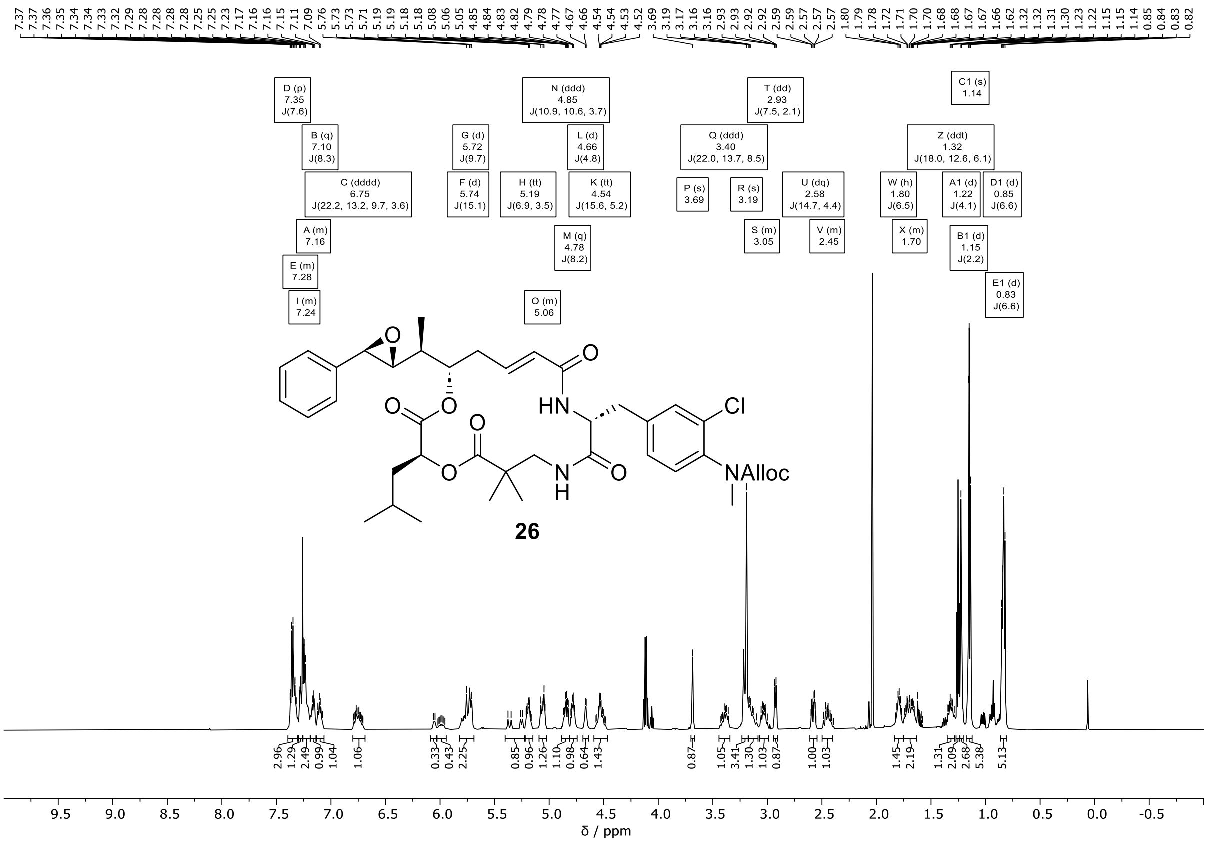

NMR spectrum 22: ^{1}H NMR spectrum (600 MHz, $CDCl_3$) of modified cryptophycin-uB[D-Phe(3-Cl)(4 NMeAlloc)] **26**.

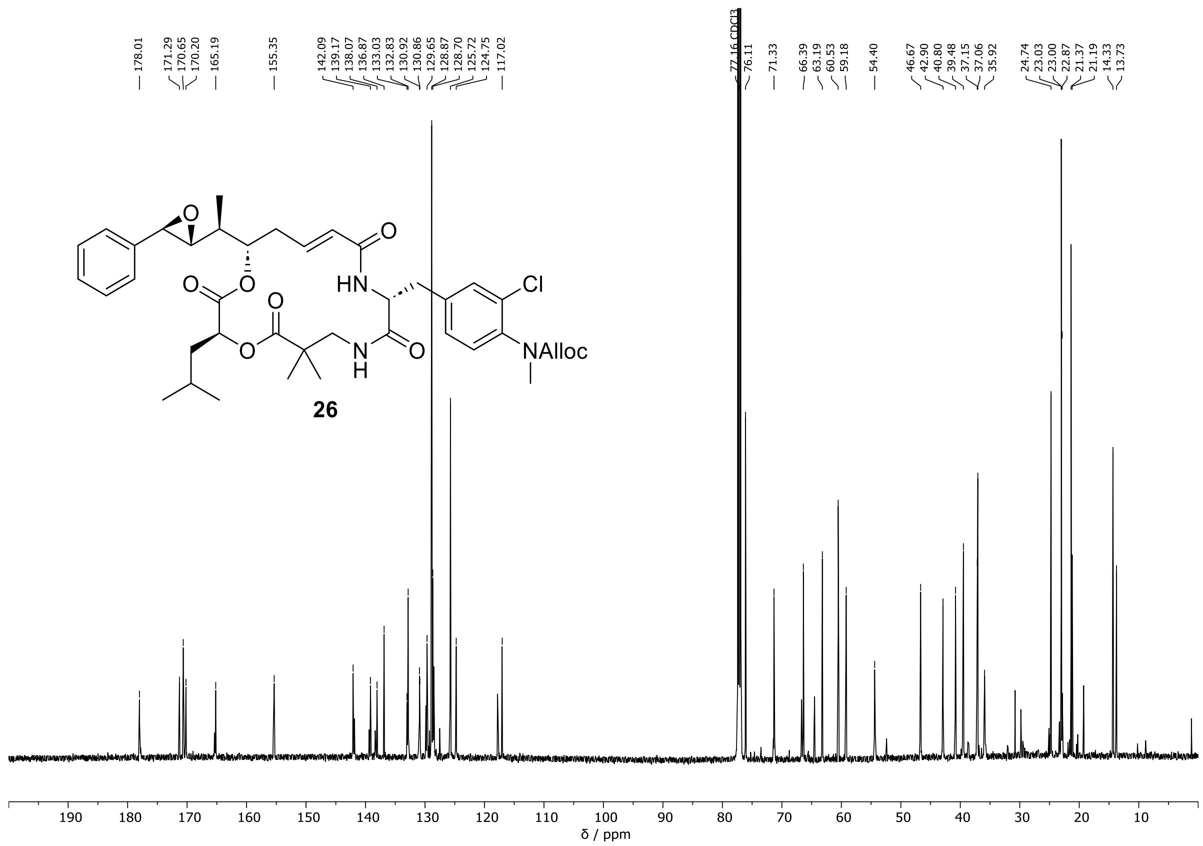

NMR spectrum 23: $^{13}C\{^1H\}$ NMR spectrum (151 MHz {600 MHz}, $CDCl_3$) of modified cryptophycin-uB[D-Phe(3-Cl)(4-NMeAlloc)] **26**.

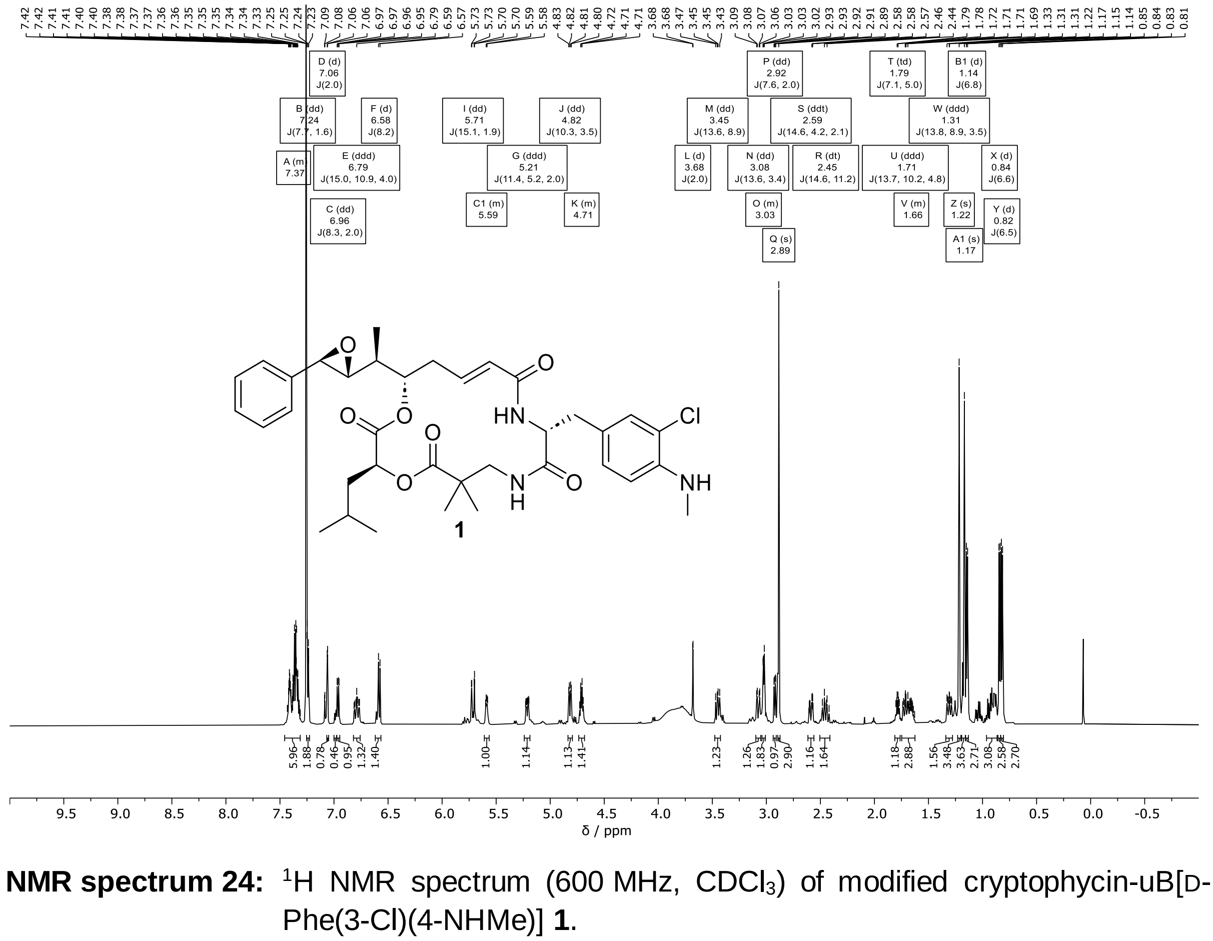

NMR spectrum 24: ^1H NMR spectrum (600 MHz, $CDCl_3$) of modified cryptophycin-uB[D-Phe(3-Cl)(4-NHMe)] **1**.

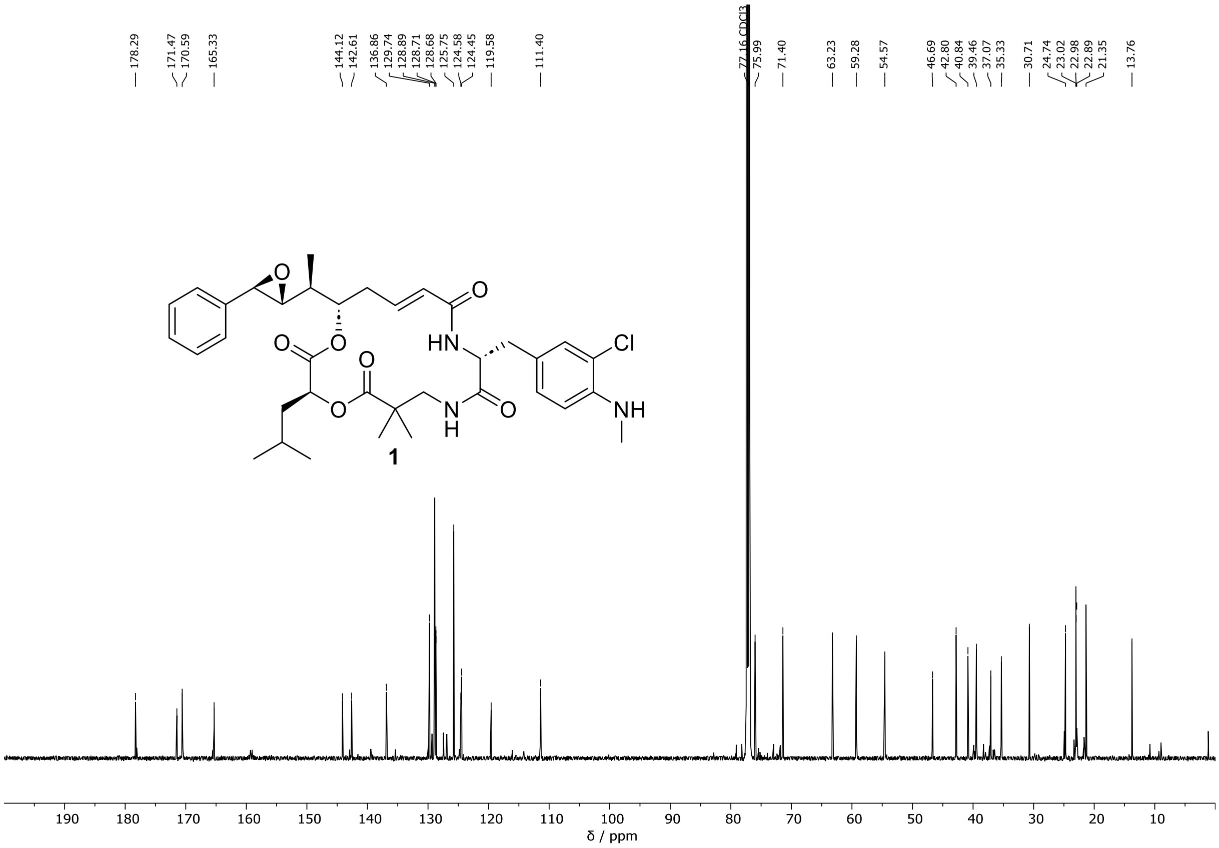

NMR spectrum 25: $^{13}C\{^{1}H\}$ NMR spectrum (151 MHz {600 MHz}, $CDCl_3$) of modified cryptophycin-uB[D-Phe(3-Cl)(4-NHMe)] **1**.

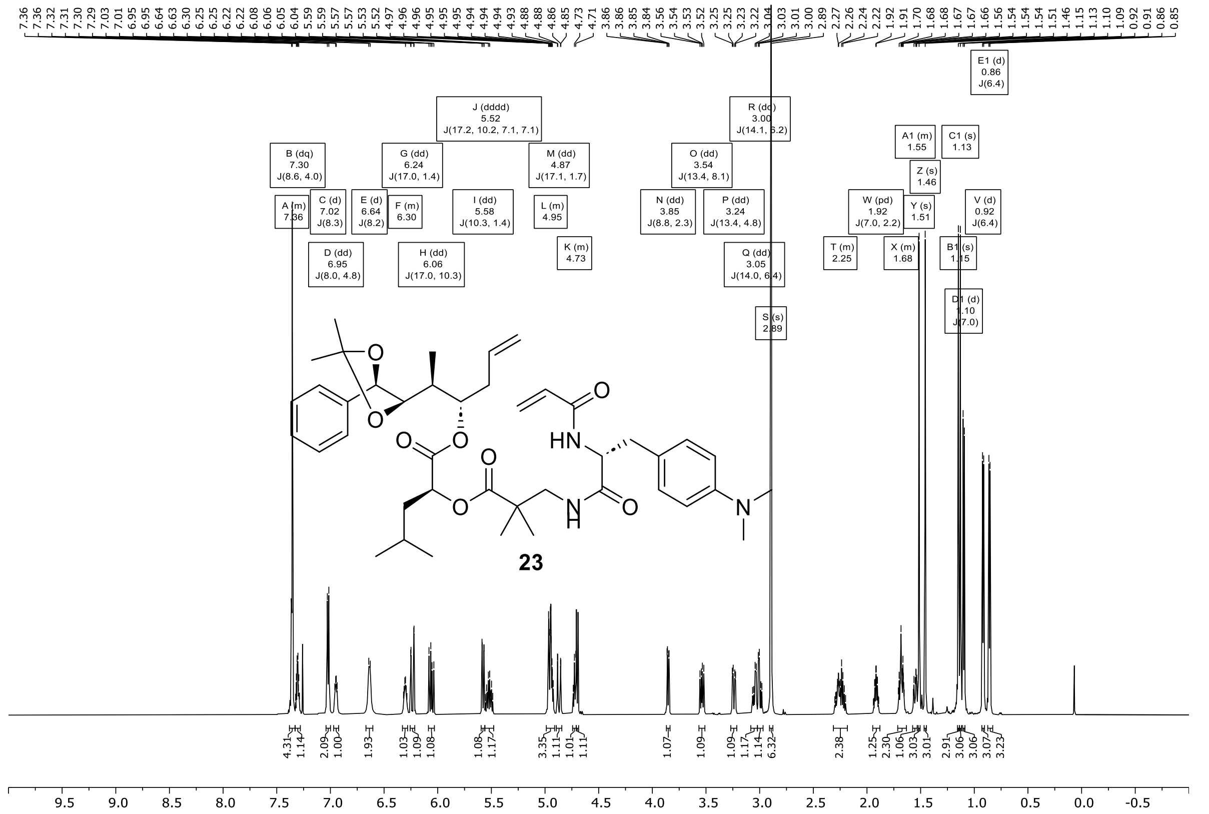

NMR spectrum 26: ^{1}H NMR spectrum (600 MHz, $CDCl_3$) of modified *seco*-cryptophycin-uB[D-Phe(4-NMe$_2$)] **23**.

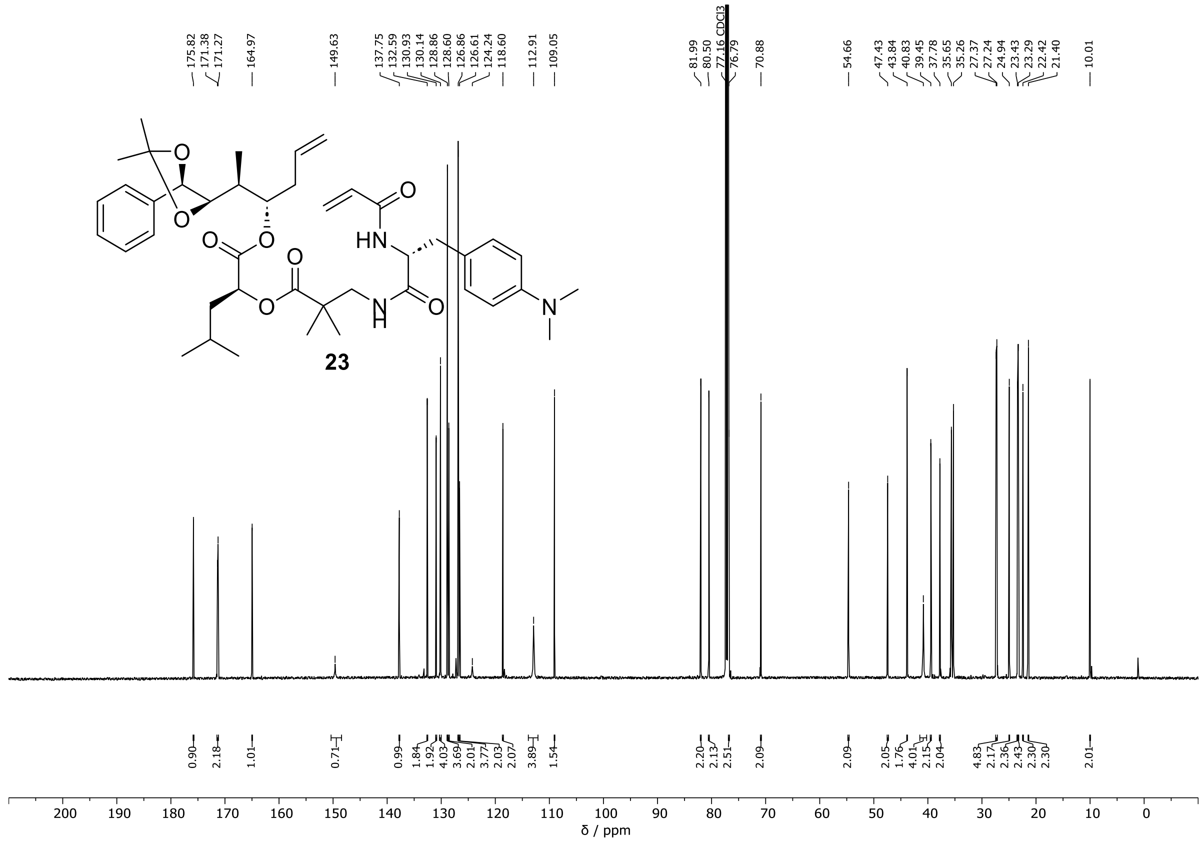

NMR spectrum 27: $^{13}C\{^{1}H\}$ NMR spectrum (151 MHz {600 MHz}, $CDCl_3$) of modified *seco*-cryptophycin-uB[D-Phe(4-NMe$_2$)] **23**.

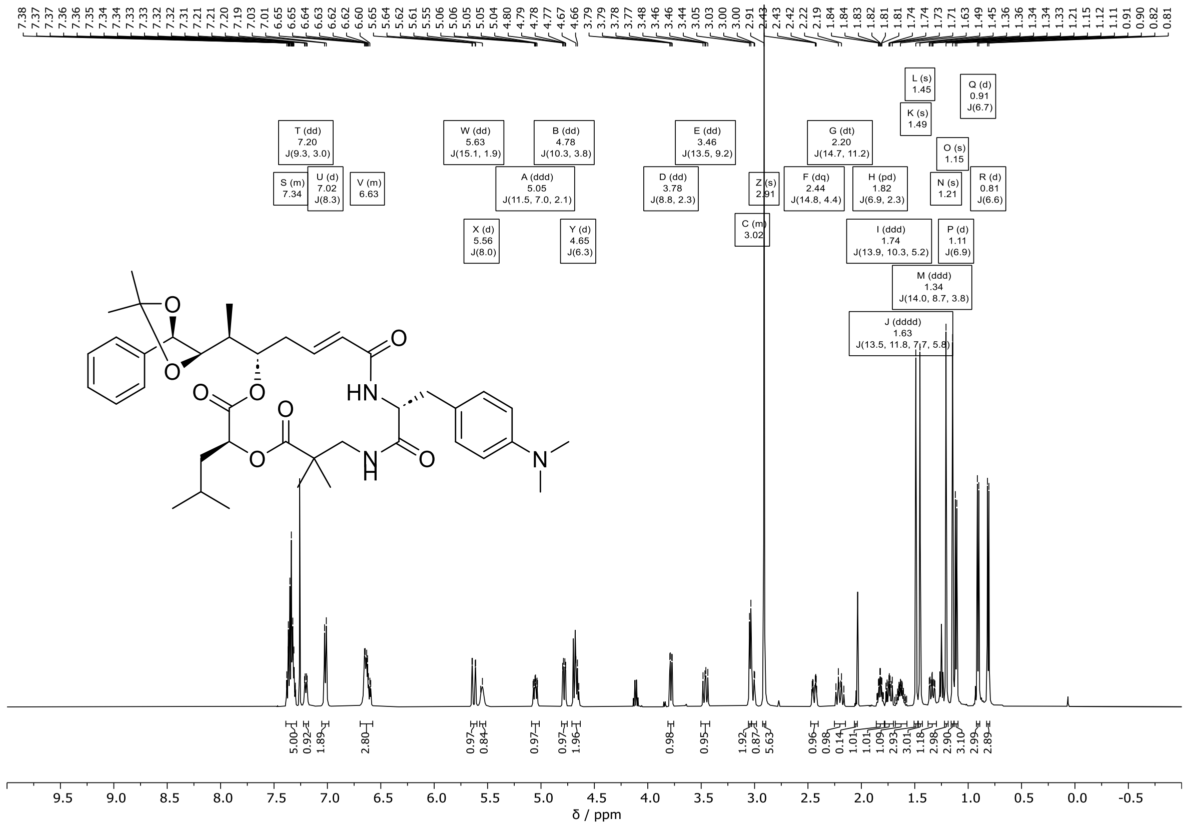

NMR spectrum 28: ^{1}H NMR spectrum (500 MHz, $CDCl_3$) of modified cryptophycin-uA[acetonide]-uB[D-Phe(4-NMe$_2$)].

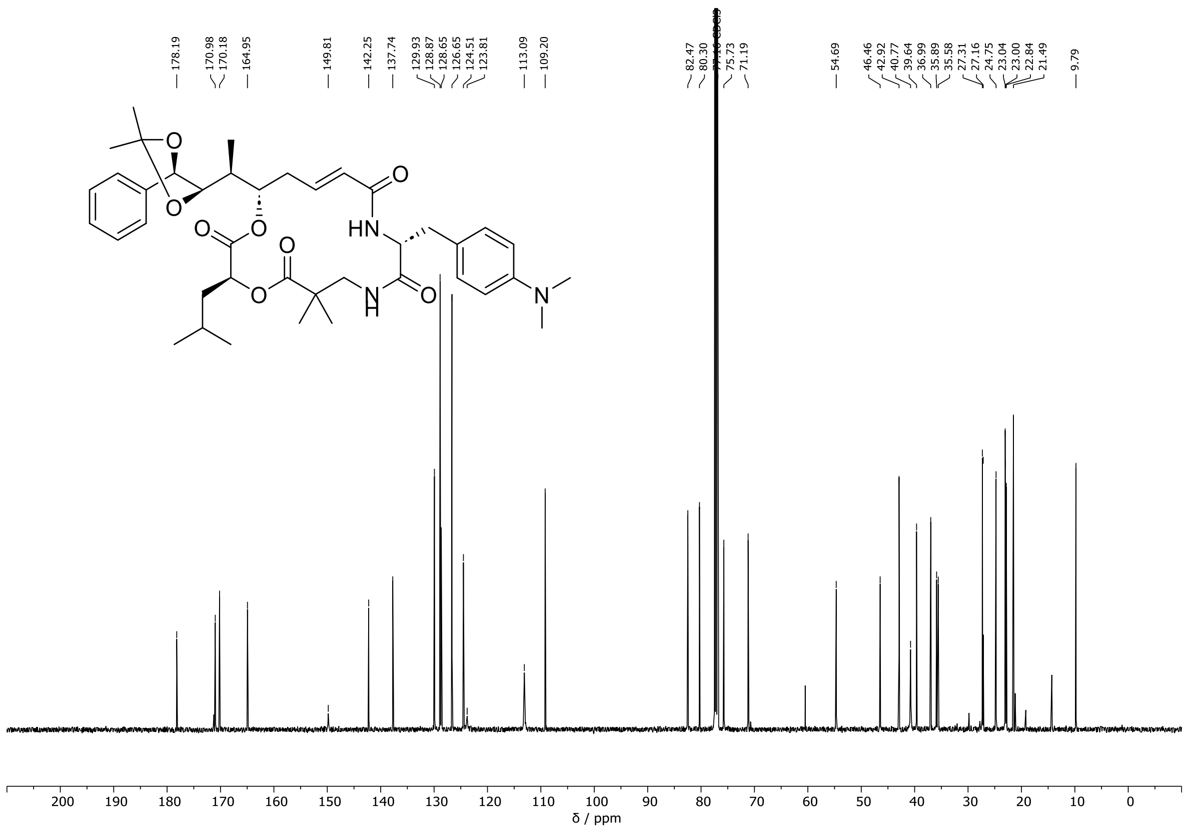

NMR spectrum 29: $^{13}C\{^{1}H\}$ NMR spectrum (125 MHz {500 MHz}, $CDCl_3$) of modified cryptophycin-uA[acetonide]-uB[D-Phe(4-NMe$_2$)].

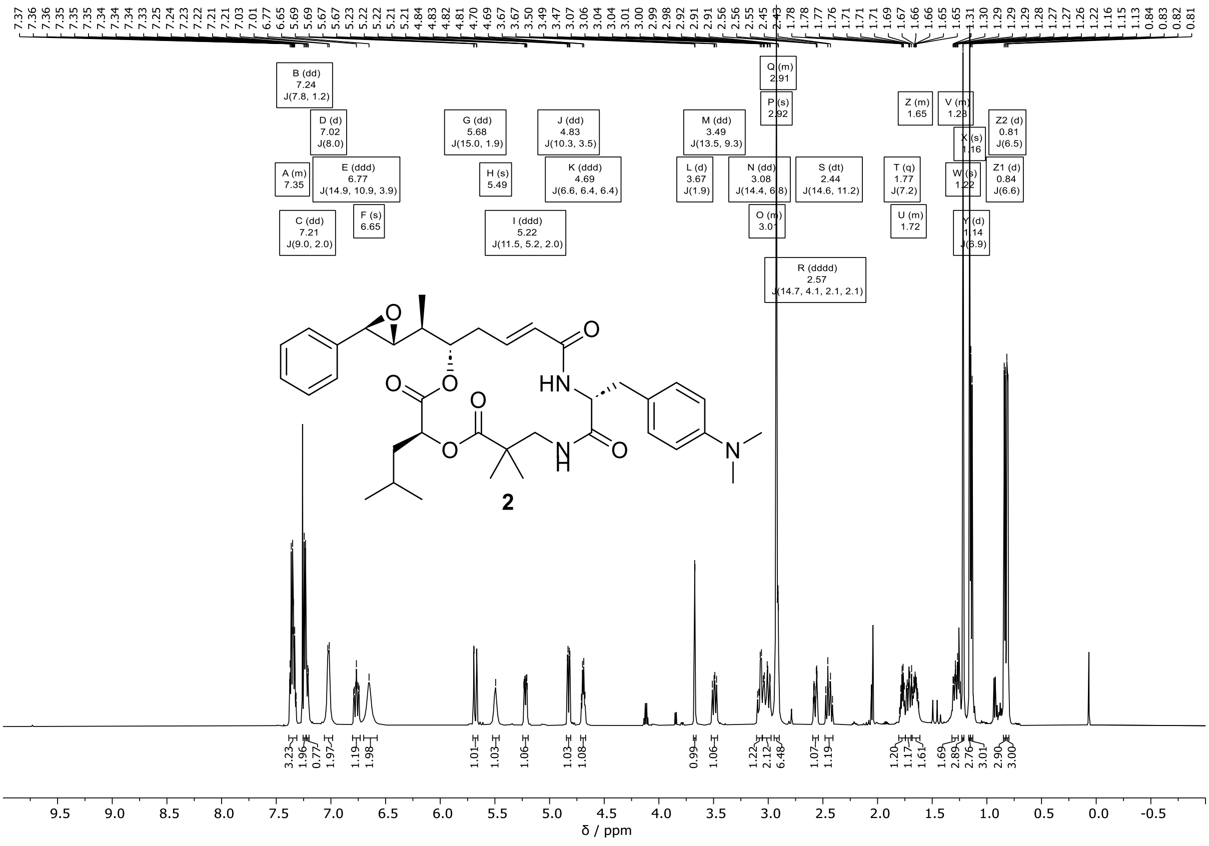

NMR spectrum 30: ^{1}H NMR spectrum (600 MHz, $CDCl_3$) of modified cryptophycin-uB[D-Phe(4-NMe$_2$)] **2**.

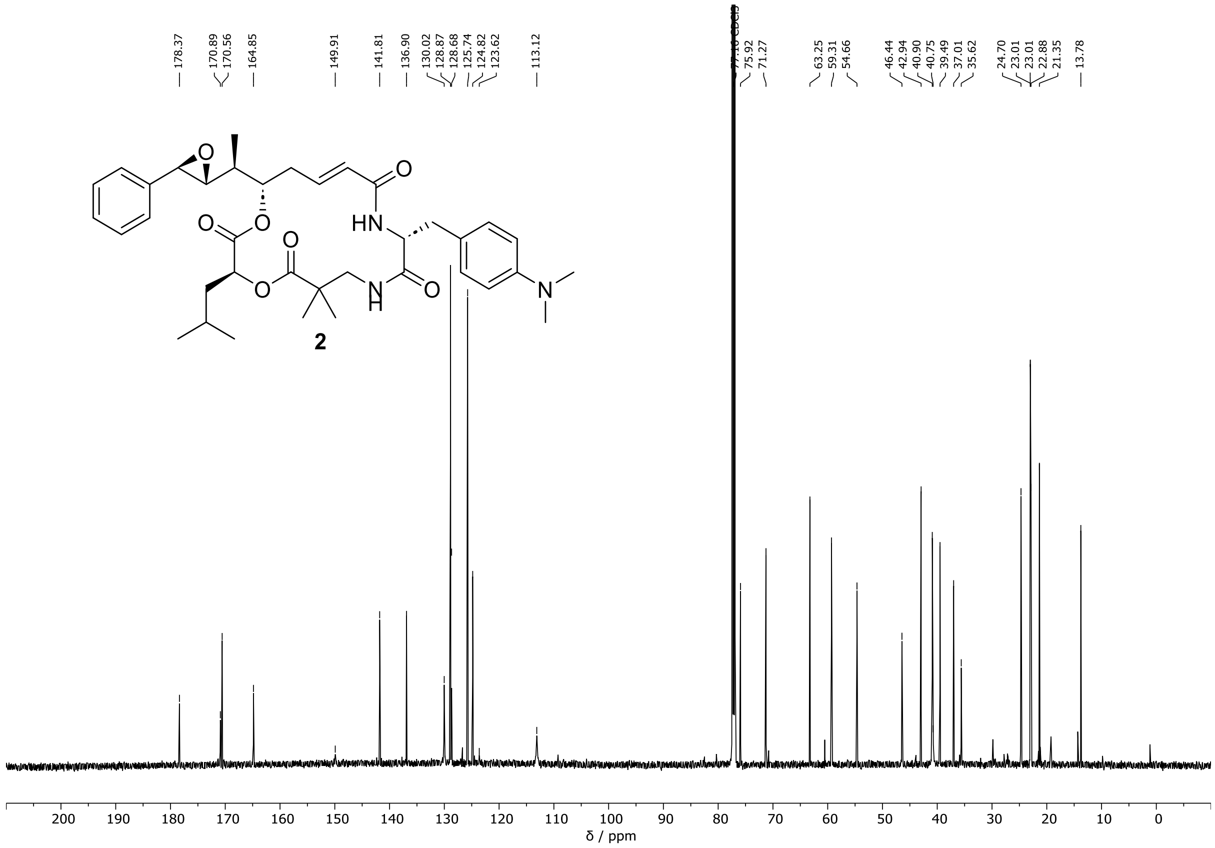

NMR spectrum 31: $^{13}C\{^{1}H\}$ NMR spectrum (151 MHz {600 MHz}, $CDCl_3$) of modified cryptophycin-uB[D-Phe(4-NMe$_2$)] **2**.

6.2 Cellular proliferation assays

Cellular proliferation assay with cryptophycin-uB[D-Phe(3-Cl)(4-NMeAlloc)] 26 against KB-3-1 Cells

Sigmoidal dose-response	
Best-fit values	
BOTTOM	-2.999
TOP	95.40
LOGEC50	-6.427
EC50	3.742e-007
Std. Error	
BOTTOM	1.076
TOP	0.6759
LOGEC50	0.02723
95% Confidence Intervals	
BOTTOM	-5.108 to -0.8894
TOP	94.07 to 96.72
LOGEC50	-6.480 to -6.374
EC50	3.309e-007 to 4.231e-007
Goodness of Fit	
Degrees of Freedom	747
R^2	0.9049
Absolute Sum of Squares	126968
Sy.x	13.04
Data	
Number of X values	16
Number of Y replicates	52
Total number of values	750
Number of missing values	82

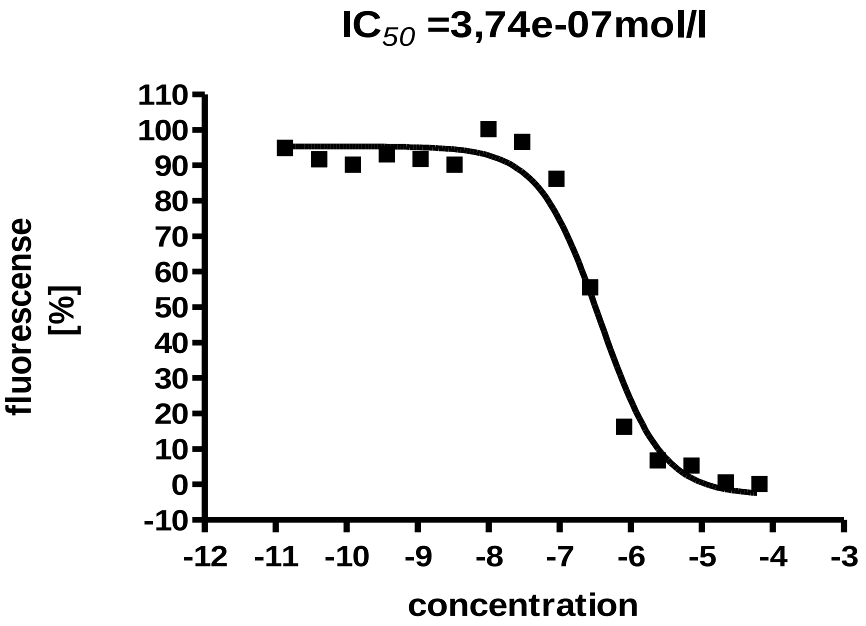

Figure S2: Cryptophycin-uB[D-Phe(3-Cl)(4-NMeAlloc)] **26** against KB-3-1 cells.

Cellular proliferation assay with cryptophycin-uB[D-Phe(3-Cl)(4-NMeAlloc)] 26 against KB-V1 Cells

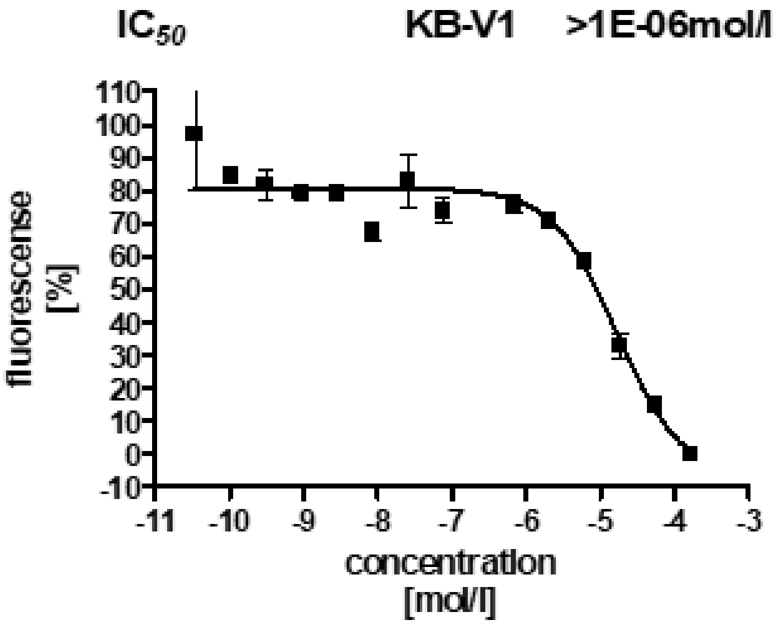

Figure S3: Cryptophycin-uB[D-Phe(3-Cl)(4-NMeAlloc)] **26** against KB-V1 cells. No proper fitting was possible due to the low toxicity of **26** ($>1\cdot10^{-6}$ M) against KB-V1 cells.

Cellular proliferaation assay with cryptophycin-uB[D-Phe(3-Cl)(4-NHMe)] 1 against KB-3-1 Cells

Sigmoidal dose-response	
Best-fit values	
BOTTOM	-0.1387
TOP	98.18
LOGEC50	-9.505
EC50	3.126e-010
Std. Error	
BOTTOM	1.478
TOP	1.309
LOGEC50	0.04500
95% Confidence Intervals	
BOTTOM	-3.035 to 2.757
TOP	95.61 to 100.7
LOGEC50	-9.593 to -9.417
EC50	2.552e-010 to 3.830e-010
Goodness of Fit	
Degrees of Freedom	522
R^2	0.8395
Absolute Sum of Squares	182010
Sy.x	18.67
Data	
Number of X values	16
Number of Y replicates	52
Total number of values	525
Number of missing values	307

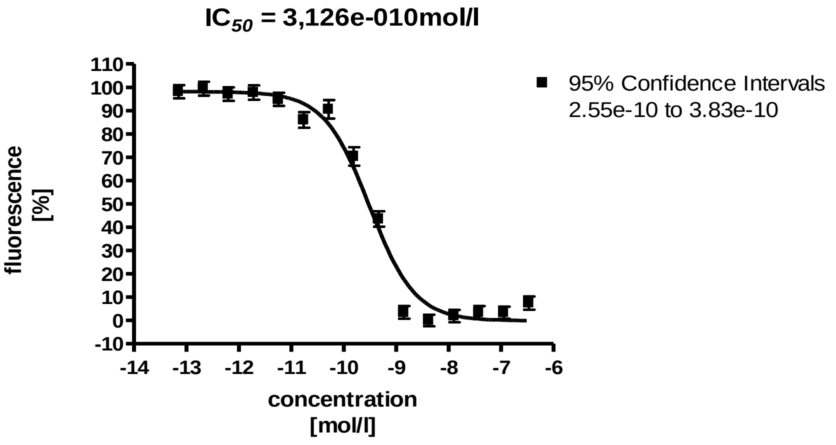

Figure S4: Cryptophycin-uB[D-Phe(3-Cl)(4-NHMe)] **1** against KB-3-1 cells.

Cellular proliferation assay with cryptophycin-uB[D-Phe(3-Cl)(4-NHMe)] 1 against KB-V1 Cells

Sigmoidal dose-response (variable slope)	
Best-fit values	
BOTTOM	0.8438
TOP	90.29
LOGEC50	-8.110
HILLSLOPE	-1.210
EC50	7.763e-009
Std. Error	
BOTTOM	0.7206
TOP	0.8737
LOGEC50	0.01915
HILLSLOPE	0.05758
95% Confidence Intervals	
BOTTOM	-0.5686 to 2.256
TOP	88.58 to 92.00
LOGEC50	-8.148 to -8.072
HILLSLOPE	-1.323 to -1.097
EC50	7.120e-009 to 8.464e-009
Goodness of Fit	
Degrees of Freedom	568
R^2	0.9436
Absolute Sum of Squares	49044
Sy.x	9.292
Data	
Number of X values	12
Number of Y replicates	52
Total number of values	572
Number of missing values	52

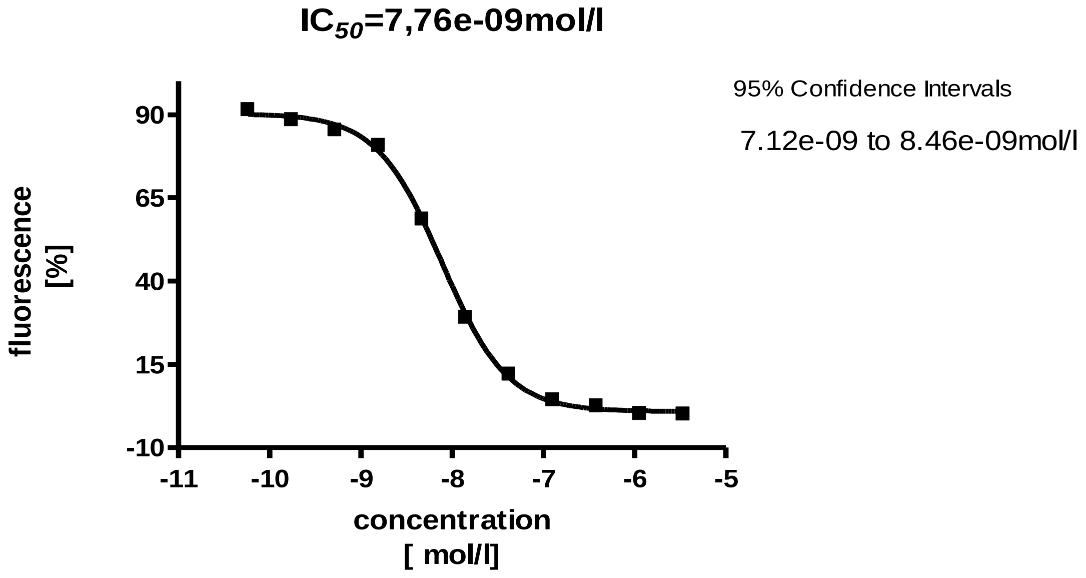

Figure S5: Cryptophycin-uB[D-Phe(3-Cl)(4-NHMe)] **1** against KB-V1 cells.

Cellular proliferation assay with cryptophycin-uB[D-Phe(4-NMe$_2$)] 2 against KB-3-1 Cells

Sig	
Bes	
TOP	93.25
LOGEC50	-8.197
EC50	6.360e-009
Std. Error	
BOTTOM	1.183
TOP	0.7045
LOGEC50	0.02881
95% Confidence Intervals	
BOTTOM	-5.534 to -0.8950
TOP	91.87 to 94.63
LOGEC50	-8.253 to -8.140
EC50	5.585e-009 to 7.244e-009
Goodness of Fit	
Degrees of Freedom	707
R^2	0.8944
Absolute Sum of Squares	127897
Sy.x	13.45
Data	
Number of X values	16
Number of Y replicates	52
Total number of values	710
Number of missing values	122

IC_{50}=6,36e-09mol/l

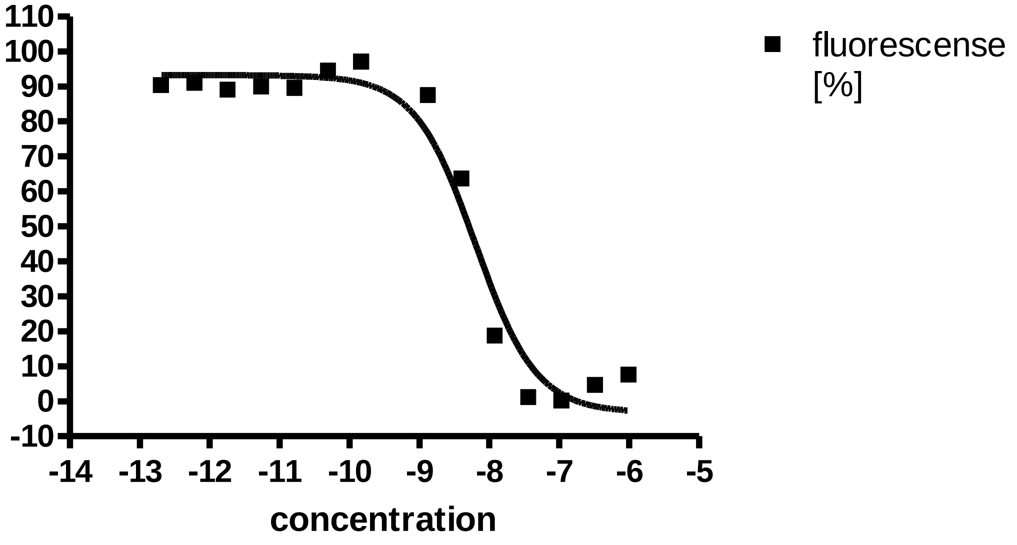

Figure S6: Cryptophycin-uB[D-Phe(4-NMe$_2$)] **2** against KB-3-1 cells.

Cellular proliferation assay with cryptophycin-uB[D-Phe(4-NMe2)] 2 against KB-V1 Cells

Sigm[illegible]	
Best[illegible]	
B[illegible]	
T[illegible]	[illegible]
LOGEC50	-6.661
EC50	2.184e-007
Std. Error	
BOTTOM	1.376
TOP	0.7742
LOGEC50	0.03354
95% Confidence Intervals	
BOTTOM	-5.808 to -0.4123
TOP	92.35 to 95.39
LOGEC50	-6.727 to -6.595
EC50	1.877e-007 to 2.541e-007
Goodness of Fit	
Degrees of Freedom	594
R^2	0.8865
Absolute Sum of Squares	112848
Sy.x	13.78
Data	
Number of X values	17
Number of Y replicates	52
Total number of values	597
Number of missing values	287

IC_{50}=2,18e-07mol/l

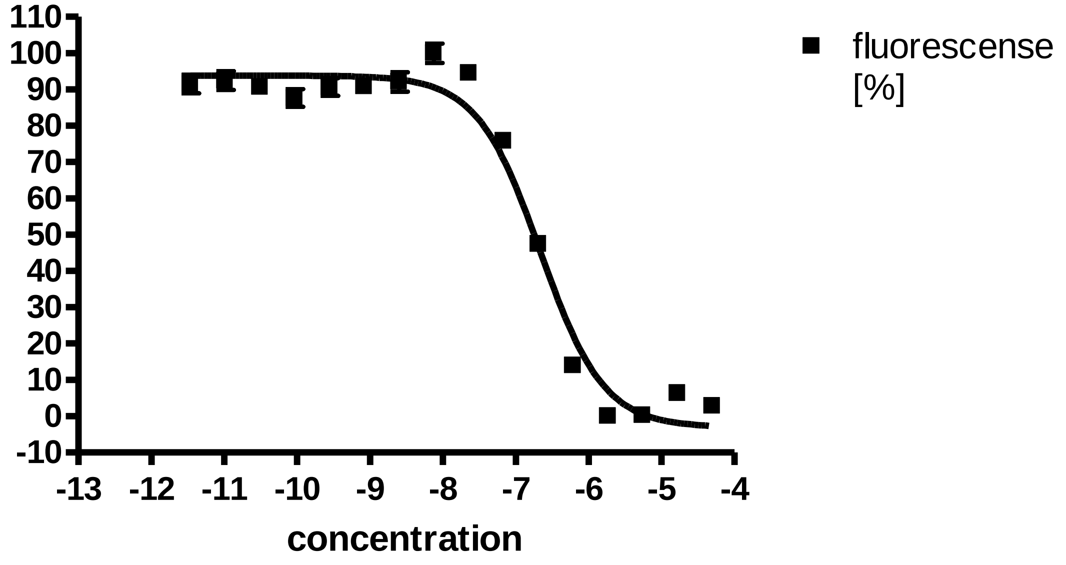

Figure S7: Cryptophycin-uB[D-Phe(4-NMe2)] **2** against KB-V1 cells.
